# Uncovering the Networks of Topological Neighborhoods in β-Strand and Amyloid β-Sheet Structures

**DOI:** 10.1038/s41598-019-47151-2

**Published:** 2019-07-24

**Authors:** Luhan Zhai, Yuko Otani, Tomohiko Ohwada

**Affiliations:** Laboratory of Organic and Medicinal Chemistry, Graduate School of Pharmaceutical Sciences, University of Tokyo, 7-3-1 Hongo, Bunkyo-ku, Tokyo, 113-0033 Japan

**Keywords:** Computational models, Biophysical chemistry

## Abstract

Although multiple hydrophobic, aromatic π–π, and electrostatic interactions are proposed to be involved in amyloid fibril formation, the precise interactions within amyloid structures remain poorly understood. Here, we carried out detailed quantum theory of atoms-in-molecules (QTAIM) analysis to examine the hydrophobic core of amyloid parallel and antiparallel β-sheet structures, and found the presence of multiple inter-strand and intra-strand topological neighborhoods, represented by networks of through-space bond paths. Similar bond paths from side chain to side chain and from side chain to main chain were found in a single β-strand and in di- and tripeptides. Some of these bond-path networks were enhanced upon β-sheet formation. Overall, our results indicate that the cumulative network of weak interactions, including various types of hydrogen bonding (X-H—Y; X, Y = H, C, O, N, S), as well as *non*-H-*non*-H bond paths, is characteristic of amyloid β-sheet structure. The present study postulated that the presence of multiple through-space bond-paths, which are local and directional, can coincide with the attractive proximity effect in forming peptide assemblies. This is consistent with a new view of the van der Waals (vdW) interactions, one of the origins of hydrophobic interaction, which is updating to be a directional intermolecular force.

## Introduction

In addition to amide hydrogen bonding, multiple interactions such as hydrophobic, aromatic π–π, and electrostatic interactions are proposed to be involved in forming peptide assemblies^1^. In Alzheimer’s disease (AD) and related diseases, amyloid-β peptide (Aβ) forms oligomers and amyloid fibrils consisting of β-sheet structures^2^, which are the main component of the characteristic neuritic plaques^3^. The amyloid fibers are heterogeneous aggregates of highly ordered and stable proteins, which share common structural and staining characteristics, but appear to have little sequence homology^1^. Much attention has been focused on understanding what drives particular peptide sequences to aggregate and how the amyloid peptide self-assembles. While hydrogen bonding is crucial, uncovering specific interactions within the amyloid structure is expected throw light on the mechanisms of fibril formation and fibril stability. Among the Aβ isoforms, Aβ40 and Aβ42 are the most abundant^4^. Aβ40 contains many hydrophobic (Val, Ile, etc.) and aromatic (Phe and Tyr) amino acids, and it has been suggested that multiple hydrophobic, aromatic, and electrostatic interactions are involved in amyloid fibril formation^3^, and that the self-assembly of amyloid peptides is mainly governed by non-covalent interactions, including hydrogen bonds, coulombic interactions and hydrophobic effects^5–9^. Aromatic π–π interactions were also proposed to contribute significantly to amyloid aggregation^10–15^, but other studies have failed to identify specific interactions involving π-electrons or aromatic character as forces that stabilize the whole fibril^16,17^. Therefore, the actual mechanism of amyloid aggregation is still controversial. However, it has been established that the hydrophobic aromatic core of Aβ40 (17–20) (Leu^17^-Val^18^-Phe^19^-Phe^20^) is an important target for pharmaceutical inhibition of Aβ neurotoxicity in Alzheimer’s disease^18^.

Amino acids bearing a branched side chain, such as Val, Ile, Thr, and Cys, and aromatic amino acids, such as Tyr, Trp and Phe, have high propensities to form β-strand structure, whereas Ala, Gly and Pro have poor propensities^19–22^. The former amino acids account for nearly 50% of the amino acids in Aβ40 and Aβ42. However, these propensities are statistical in nature, and more rational explanations for the differences in intrinsic β-strand-forming propensities of these amino acids remain to be explored^23^.

Bader’s quantum theory of atoms in molecules (QTAIM)^24^ is the topological analysis of the molecular electron density based on zero-flux surfaces. This partitioning of electron density by means of the QTAIM is well defined and can be applied to electron-density distributions obtained from both experiment and theory. A bond path^25^ is a single line linking the two electron attracters (usually nucleus), minimum electron density linking the nuclei of two chemically bonded atoms^24,26^. There is a minimum electron-density along the bond path, a bond critical point. The entire bond path runs from the first nucleus over the bond critical point to the second one. A bond path of strong interaction such as in a covalent bond almost always corresponds to a chemical bond. On the other hands, it has been repeatedly defined that bond paths are not chemical bonds, particularly in the cases of weak interactions (see also Methods: What is the bond path?)^27–31^. While QTAIM has been applied to many types of compounds^32^, including peptides, detecting covalent bonding^33–35^, and more recently to α-helix structures of peptides, which reasonably identified amide hydrogen-amide carbonyl oxygen hydrogen bonds^36^, there have been few studies of β-strand and β-sheet structures relevant to amyloid-β peptide (Aβ). In this study, we employed QTAIM analysis to examine the hydrophobic and aromatic amino acids of Aβ-peptides, and found multiple inter-strand and intra-strand bond-path networks between specified atom pairs in the hydrophobic and aromatic amino acids of Aβ-peptides. The former inter-strand bond-path networks, representing topological neighborhoods, are characteristic of β-strand/sheet structures, and are never found in α-helical structure^36^. We propose that intrinsic ability to form multiple through-space bond paths is relevant to β-strand/sheet structures that may promote inter-strand association of Aβ peptides. Indeed, some of these bond-path networks were found to be enhanced upon β-sheet formation.

## Methods

### Conformations of peptides

The initial conformations of various dipeptides (Ac-X-NHMe, X = amino acid), tripeptides, a single β-strand, parallel and antiparallel β-sheets of Aβ40 were obtained from conformation search or a protein crystallographic database.

**Figure 1:**
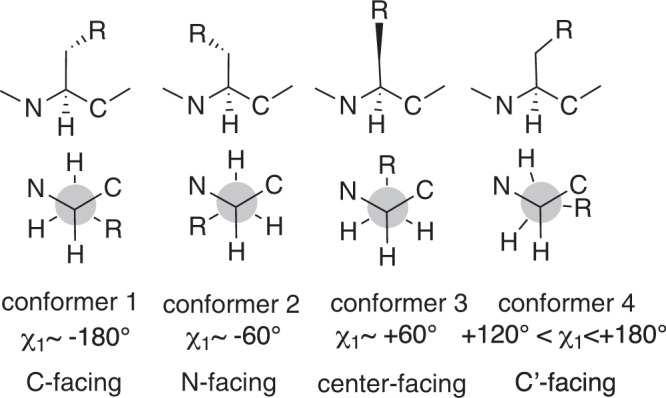
Configurations of the side chain.

For dipeptides, the initial PPII (polyproline II helix) and β structures of the dipeptide were obtained by means of Monte Carlo torsional sampling conformational search with OPLS3 force field in the Maestro program (Schrödinger, Inc., USA.)^37^, with a 10 kcal/mol energy window in water and in chloroform. Ten amino acid dipeptides (Ac-X-NHMe: X = Ala, Pro, Leu, Val, Ile, Thr, Cys, Phe, Tyr, and Trp) were calculated. The initial conformers (10~20 conformers for PPII and β structures, respectively), obtained by the conformation search were subjected to geometry full-optimizations and frequency calculations at the M06-2X/6-31 + G(d) level, using the Gaussian 09 and Gaussian 16 suites of programs^38^. The M06-2X/6-31 + G(d) level method was used previously and this calculation method was demonstrated to correctly predict the backbone conformational populations of proline dipeptide and its derivatives in water and in chloroform^39^. Therefore we adopted this method in this work. Solvent effects were evaluated by optimizing each conformer using an implicit solvent model, SMD solvation model^40^, in water and in chloroform. The lack of negative frequencies confirmed that all conformers truly represent energy minima. The structures obtained by the conformation search and those obtained by the following DFT optimization were essentially consistent. The DFT-optimized structures were compared and could be converged into 2–4 conformers. These conformers are based on the different rotations of the side chain, distinguished by the dihedral angle χ_1_(°)(∠N-Cα-Cβ-Xγ; X = heteroatom (not H)) (see Fig. 1).

The initial structures of tri-peptides were taken from the crystallographic data in the PDB database. Capping of the N-terminal (with acetyl) and C-terminal (with NHMe) was automatically executed in the Maestro software, which can mimic the peptide main chain. The tri-peptides examined in this work were Ac-Val-Val-Val-NH_2_ (**VVV** from the β-strand sequence V^66^-V^68^ of PDB entry 1EST)^41^ Ac-Leu-Val-Phe-NH_2_ (**LVF** from the β-strand sequence L^101^-F^103^ of PDB entry 1AXC)^42^, Ac-Ile-Thr-Tyr-NH_2_ (**ITY** from the β-strand sequence I^205^-Y^207^ of PDB entry 2PEC)^43^, and Ac-Leu-Val-Ile-NH_2_ (**LVI** from the β-strand sequence L^52^-I^54^ of PDB entry 2PEC)^43^, which were compared with the PPII structures found in Ac-Gly-Pro-Ala-NH_2_ (**GPA** from the PPII sequence G^24^-A^26^ of PDB entry 451C)^44^ and Ac-Gly-Pro-Ser-NH_2_ (**GPS** from the PPII sequence G^123^-S^125^ of PDB entry 3FZU)^45^. The selection of the β-strand sequences, **VVV**, **LVF**, **ITY**, **LVI**, was based on their availability in the X-ray crystal structure database. PDB entry 1EST (porcine pancreatic elastase)^41^ and 2PEC (tri β-strand coiled folds of pectate lyases) are classified as “all beta proteins”^43^. PDB entry 1AXC (C-terminal region of p21^WAF1/CIP1^ complexed with Human PCNA) is classified as “alpha and beta proteins”^42^. The PPII sequences, **GPA**, and **GPS** were used for comparison with the β-strand sequences. PDB entry 451C (Cytochrome C_551_ from *Pseudomonas aeruginosa*) is classified as “all alpha proteins”^44^ PDB entry 3FZU (lgG1) is classified as “mainly beta”^45^. All the crystal structures were relaxed by energy minimization with force field OPLS3 in Maestro, followed by DFT structure optimization calculations at the M06-2X/6-31 + G(d) level with the SMD solvation model in water and in chloroform^40^. These solvents were selected on the basis of the different dielectric constants, which would influence the folding properties of peptides. The structures obtained by energy minimization with force field or those obtained by the DFT optimization were almost identical with the initial crystallographic structures (except **ITY** in water; the structure ITY changed).

The single strand structure was taken from F^64^-V^68^ of PDB entry 1EST^41^. Parallel β-sheet and anti-parallel β-sheet structures were extracted from Aβ40 (PDB 2LMP: K^16^-A^21^)^46^ and Aβ40 (PDB 2LNQ_A: K^16^-N^22^, 2LNQ_B: K^16^-Q^22^)^47^, respectively. For the single strand and β-sheet structures, we directly used the structures extracted from the protein crystal structures for the QTAIM analysis.

### QTAIM calculations

Bond-path analysis was performed at the Slater-type triple-zeta-polarization (TZP) level with ADF (SCM, Netherlands)^48^. A polarization function is added for H through Ar and for Ga through Kr. The detail of optimization of Slater-type basis sets was reported previously^49^. The QTAIM analysis was applied on the basis of the DFT-optimized energy-minimum structures and PDB database structures.

### What is the bond path?

**Figure 2:**
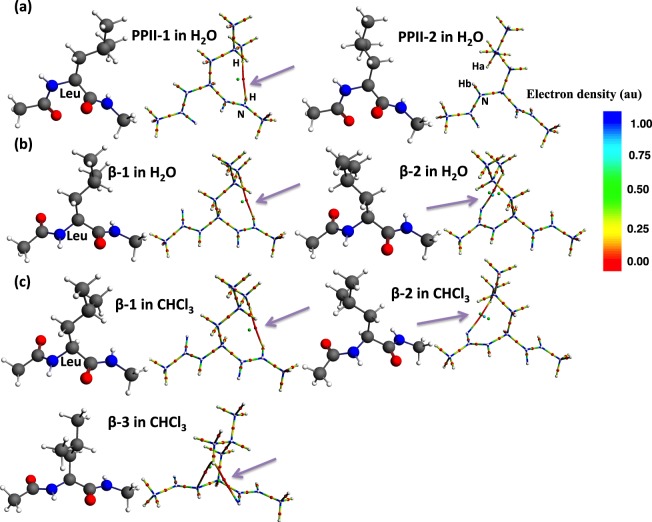
Leu dipeptide (Ac-Leu-NHMe) in water and in chloroform.

The concepts of bond paths and bond critical points have been criticized^50–54^ and a rebuttal published^27^. There have been also arguments of the presence/absence and the interpretation of H—H bond path in different systems (see Results and Discussion)^28,55–57^. A bond path is often misidentified with a chemical bond, but bond paths have been regarded as indicative of *bonded interactions*, not chemical bonds, which are claimed to encompass all kinds of interactions^28^. Usually covalent bonding corresponds well to the bond path. One of the different interpretations of a bond path of *weak interactions* is that “simply allowing two atoms to approach each other should often cause electron density to flow to the interatomic space, depending on the balance between nucleus-electron attraction and electron-electron repulsion, both coulomb and exchange”^54,55^. QTAIM atoms are not simple spheres thus their neighborhood can be influenced by their complex topology. This can lead to a bond path with a bond critical point, even in the purely classical case in which exchange is not considered^29–31^. This analysis suggests that the occurrence of a bond critical point should depend on the interatomic distance^53,54^. However, in the present cases, the H-H bond path we detected in the Leu dipeptides did not always show the closest set of the two atoms: for example, the distance between the two atoms (H-H(N)) connected by a bond path in the PPII-2 structure in water is 2.347 Å while the distance between the corresponding same atom pair, H_a_-H_b_(N) in the PPII-2 structure in water is 2.233 Å, much shorter than the former (see Fig. 2(a)). However this is no bond path between the relevant H_a_-H_b_(N) atom pair. This indicated that *geometrical proximity* is not only a factor for the presence of a bond path. Therefore, bond paths, including the present H—H interactions observed in the Leu dipeptide and other dipeptides studied (vide infra) are consistent with the *topological proximity* of these relevant atoms and also indicative of weak interactions at least in the present peptide system.

## Results and Discussion

### Conformational preferences and bond paths of dipeptide

It has been reported that amino acids that favor β-strands in proteins are side-chain branched such as in Leu, Val, Ile, Thr, and Cys and aromatic such as in Phe, Tyr and Trp^22^. Thus, we focused on amino acids having a branched side chain or aromatic side chain, such as Leu, Val, Ile, Thr, Cys, Phe, Tyr and Trp. We showed the data of Lue, Phe and Trp dipeptides in the main text and showed the other data of Val, Ile, Thr, Cys and Tyr in Supplementary Data (see Supplementary Figs 3–7). The results of other dipeptides of Ala and Pro were also shown in Supplementary Data (see Supplementary Figs 1 and 2).

First, we studied through-space weak interactions in the β-strand conformations of the ten short amino acid dipeptides (Ac-X-NHMe, X = Ala, Pro, Leu, Val, Ile, Thr, Cys, Phe, Tyr, and Trp) in terms of bond paths. In order to examine whether the interaction is β-strand-specific or not, we compared the β-strand structure with the PPII structure of the same molecule. Several accessible conformers are based on the different rotations of the side chain, distinguished by the dihedral angle χ_1_(°) (Fig. 1, see also Supplementary Table 1): conformer 1: C-*terminal*-facing, dihedral angle χ_1_ ~ −180°; conformer 2: N-*terminal*-facing, dihedral angle χ_1_ ~ −60°; conformer 3: center-facing, dihedral angle χ_1_ ~ 60°; and conformer 4: C’-*terminal*-facing, dihedral angle 120° < χ_1_ < 180°). Both of conformer 1 (C-*terminal*-facing) and conformer 4 (C’-*terminal*-facing) are classified into C-terminal facing.

Supplementary Table 1 shows calculated main chain torsion angles (Φ and Ψ) of the local energy minimum structure of ten dipeptides obtained in water and in chloroform solvent environments after optimization by the DFT method. Energy differences of each conformer were also compiled in Supplementary Table 1. The energy difference is insignificant (in most cases, within 1.0–0.5 kcal/mol) and these energies are essentially based on enthalpy not Gibes free energy. These conformers are in equilibrating.

### Leu dipeptide

In the case of Leu, two PPII structures (conformer 1, PPII-1(C-facing) and conformer 2, PPII-2(N-facing)) with different directions of the side chain are identified in water (Fig. 2(a)). While the conformer 2 of PPII-2(N-terminal-facing) structure contained no through-space bond path, in the conformer 1 of PPII-1 structure (C-facing), there is a through-space bond path between methyl-H of the side chain and the main chain N-H (Fig. 2(a)). Consequently the cyclic structure was formed, which make a ring critical point (green small ball) (Fig. 2(a)). Two β-strand structures (conformer 1, β-1(C-facing) and conformer 2, β-2(N-facing)) with different directions of the side chain are also obtained in water, and three β-strand structures (conformer 1, β-1(C-facing); conformer 2, β-2(N-facing); and conformer 3, β-3(center-facing)) are obtained in chloroform (Fig. 2(b,c)). In C-facing β-1 structures detected in water and in chloroform (Fig. 2(b,c)), the side chain is rotated to the C-terminal side of Leu and there is a bond path between methyl-H of the side chain and the main chain (N-)H in both solvents. As for the N-facing β-2 structures obtained in water and in chloroform (Fig. 2(b,c)), when the side chain is rotated to the N-terminal side, a bond path between methyl-H of the side chain and the acetyl carbonyl oxygen (O) is found. Furthermore, in center-facing β-3 in chloroform (Fig. 2(c)), when the side chain is rotated to the middle position, two bond paths are generated: the methyl-H of the side-chain and the carbonyl oxygen atom (O) of the C-terminal, and the same methyl-H and N atom, forming a cyclic bonding.

In the ball-and-stick model, the colors have the following meaning: red: oxygen; blue: nitrogen; dark grey: carbon; white: hydrogen. In the molecular graph (QTAIM): the colors have the following meaning: a color-gradation line: accumulation of electron density (bond path for covalent bonding; red line: through-space weak bond path (with a purple arrow); red small ball: bond critical point, green small ball: ring critical point or cage critical point.

### Phe dipeptide

**Figure 3:**
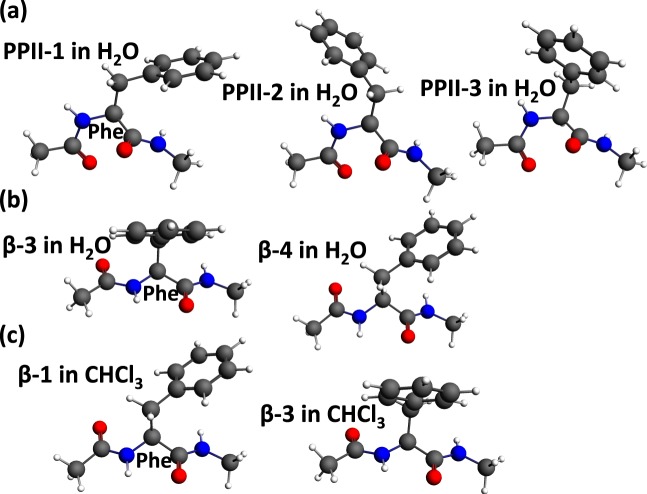
Aromatic dipeptides, Phe dipeptide in water and in chloroform.

For the aromatic amino acid Phe dipeptide, three PPII structures with the side chain (PPII-1, PPII-2 and PPII-3) directed to the C-terminal, N-terminal and middle are obtained in water (Fig. 3(a)). Two β-strand structures (β-3 and β-4 in water, β-1 and β-3 in chloroform) with the different side chain rotation are obtained in water and chloroform, respectively (Fig. 3(b,c)). No through-space bond path is detected in any of these structures.

### Trp dipeptide

**Figure 4:**
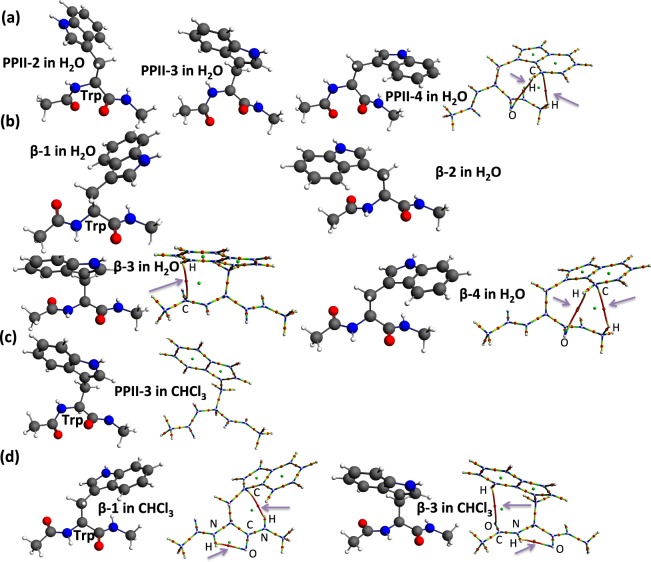
Aromatic dipeptides, Trp dipeptide in water and in chloroform.

On the other hands, for another aromatic amino acid dipeptide, Trp, three PPII structures (PPII-2, PPII-3 and PPII-4) with different side chain directions are obtained in water (Fig. 4(a)). Only the C’-facing PPII-4 structure in water contains two bond paths. One is between indole ring-H of the side chain and the main chain carbonyl oxygen (O), and the other is a C-H—π bond path between the indole ring and H of C-terminal methyl (Fig. 4(a)). Four kinds of β-strand structures (β-1, β-2, β-3 and β-4) with different side chain directions are obtained in water (Fig. 4(b)). In center-facing β-3 structure in water with the side chain rotated to the middle (Fig. 4(b)), there is a bond path between the indole ring-H and the N-terminal acetyl carbonyl carbon (C). In C’-facing β-4 structure in water, there are two bond paths between the indole ring-H with the N-terminal acetyl carbonyl oxygen and C-H—π bond path between the indole ring and H of C-terminal methyl. However, no bond path is found in C-facing β-1 structure or N-facing β-2 structure in water. One center-facing PPII-3 structure is obtained in chloroform and no bond path is found (Fig. 4(c)). Two β-strand structures (β-1 and β-3) appear in chloroform (Fig. 4(d)). In C-facing β-1 structure in chloroform, when the side chain is rotated to the C-terminal side, there are two bond paths. One is an intramolecular hydrogen bonding. The other is N-H—π interaction between the indole ring and the C-terminal H(-N) (Fig. 4(d)). In center-facing β-3 structure in chloroform, the side chain is rotated to the middle and there are two bond paths. One is an intramolecular hydrogen bond between carbonyl-O and H(-N). The other is between indole ring-H and acetyl carbonyl oxygen (O).

The data on atom pairs, bond lengths, and electron density of through-space bond paths found in the β-strand structures of the dipeptides (Leu, Val, Ile, Thr, Cys, Trp) are summarized in Table 1. We observed multi-center bonds whcih can be formed by small electron sharing between a single atom and many other atoms, or electron sharing between groups of atoms. It was proposed that multi-center bonding is not termed as bonded/non-bonded, rather neighboring/non-neighboring^29^.

**Table 1:** Atom pair, bond length, and electron density of the bond paths in the β-strand structures of the selected dipeptides.

Entry	β-Strand of dipeptide	Atom pair	Distance (Å)	Electron density at the bcp (a.u.)^a^
1	**Leu** β-1 in H_2_O	C-H···H-N	2.365	0.0056
2	**Leu** β-2 in H_2_O	C-H···O=C	2.747	0.0065
3	**Leu** β-1 in CHCl_3_	C-H···H-N	2.329	0.0059
4	**Leu** β-2 in CHCl_3_	C-H···O=C	2.595	0.0083
5	**Val** β-3 in H_2_O	C-H···N-C	2.761	0.0089
6	**Val** β-2 in CHCl_3_	C-C···O=C	3.279	0.0064
7	**Val** β-3 in CHCl_3_	C-H···N-C	2.713	0.0096
8	**Ile** β-1 in H_2_O	C-H···H-N	2.347	0.0054
9	**Ile** β-1 in H_2_O	C-H···N	2.769	0.0085
10	**Ile** β-3 in H_2_O	C-H···O=C	2.827	0.0067
11	**Ile** β-1 in CHCl_3_	C-H···N	2.698	0.0096
12	**Ile** β-2 in CHCl_3_	C-H···O=C	2.778	0.0071
13	**Thr** β-1 in H_2_O	N-H···O-C	2.432	0.0124
14	**Thr** β-2 in H_2_O	O-H···O=C	1.845	0.0338
15	**Thr** β-1 in CHCl_3_	N-H···O-C	2.120	0.0206
16	**Thr** β-2 in CHCl_3_	O-H···O=C	1.837	0.0344
17	**Thr** β-3 in CHCl_3_	C-C···O=C	3.344	0.0076
18	**Cys** β-1 in CHCl_3_	N-H···S-C	2.709	0.0129
19	**Trp** β-3 in H_2_O	C_sp2_-H···C-C	2.732	0.0066
20	**Trp** β-4 in H_2_O	C_sp2_-H···C=O	2.956	0.0046
21	**Trp** β-4 in H_2_O	C-H···C (sp_2_, π)	2.850	0.0059
22	**Trp** β-1 in CHCl_3_	N-H···C (sp_2_, π)	2.826	0.0106
23	**Trp** β-1 in CHCl_3_	N-H···O=C	2.245	0.0266
24	**Trp** β-3 in CHCl_3_	C_sp2_-H···C=O	3.037	0.0045
25	**Trp** β-3 in CHCl_3_	N-H···O=C	2.203	0.0234
26	**Hydrogen bond** ^b^	N-H···O (=C)	2.243	0.014
27	**Hydrogen bond** ^b^	C-H···O (=C)	2.729	0.006

^a^bcp = bond critical point. ^b^In α-helix of peptides^36^.

There are eight types of atom pairs involved in the bond paths observed in the dipeptides, that is, H···H, H···N, H···C, H···O, H···S, C···O and C-H···π-C and N-H···π-C pairs. The values of the electron densities at the bond critical points fall in the range of 0.0045–0.0344 (au). Among these bond paths, the C_sp2_-H···O=C bond path (**Trp** β-3 in CHCl_3_, entry 24 in Table 1) is the smallest and O-H···O=C bond path (**Thr** β-2 in CHCl_3_, entry 16 in Table 1) is the largest in terms of accumulation of electrons at the bond critical points. In the case of an X-H bond path, for a similar distance of the atom pair, atoms (X) with strong negativity always form a relatively stronger bond path based on electron density. For example, compared with H···H and C···H bond paths, H···O, H···S and H···N bond paths are always of greater strength in terms of the electron density at the bond critical point.

Based on the bond path study of amino acid dipeptides (Leu, Val, Ile, Thr, Ala, Pro, Phe, Tyr, Trp), bond paths can be observed in both PPII and β-strand structures. But, compared with PPII structures, bond paths are more extensively found in β-strand structures of the dipeptides. Because the bond path is located between the side chain and the main chain, we assume that the presence of this kind of side chain-main chain linking, that is, topological neighborhood, is a characteristic feature of β-strand structure, not of PPII structure. Amino acids bearing branched side chains often favor β-strand structure, which is consistent with the present observation that the side chain can form bond paths on both the N- and C-terminal of the main chain. In the present conformational search study, it proved very difficult to find PPII structure in chloroform, but we could obtain PPII structure in water. Therefore, in a water environment, both PPII and β-strand structure can be stabilized, while only β-strand structure can be stabilized in chloroform.

### Conformational preferences and bond paths of tri-peptides

We next applied bond-path calculation to six selected tri-peptide sequences. All the initial structures are crystal structures isolated from protein crystals. Two are β-strand-disfavoring sequences (**GPA** from the PPII sequence G^24^-A^26^ of PDB entry 451C^44^, **GPS** from the PPII sequence G^123^-S^125^ of PDB entry 3FZU)^45^ and the other four sequences (**LVF** from the β-strand sequence L^101^-F^103^ of PDB entry 1AXC^42^, **ITY** from the β-strand sequence I^205^-Y^207^ of PDB entry 2PEC^43^, **VVV** from the β-strand sequence V^66^-V^68^ of PDB entry 1EST^41^, **LVI** from the β-strand sequence L^52^-I^54^ of PDB entry 2PEC)^43^ are taken from the crystal structures of β-strand parts or β-hairpins of proteins (see Methods: Conformation of peptides). All structures were optimized using the M06-2X/6-31 + G(d) method with the SMD solvation model in water and in chloroform^40^. DFT-optimized structures and original crystal structures showed no significant changes of the structures in terms of main chain and side chain angles (See Supplementary Information, except **ITY** in water: the structure was changed). Selected main chain torsion angles are shown in Supplementary Table 2.

**Figure 5:**
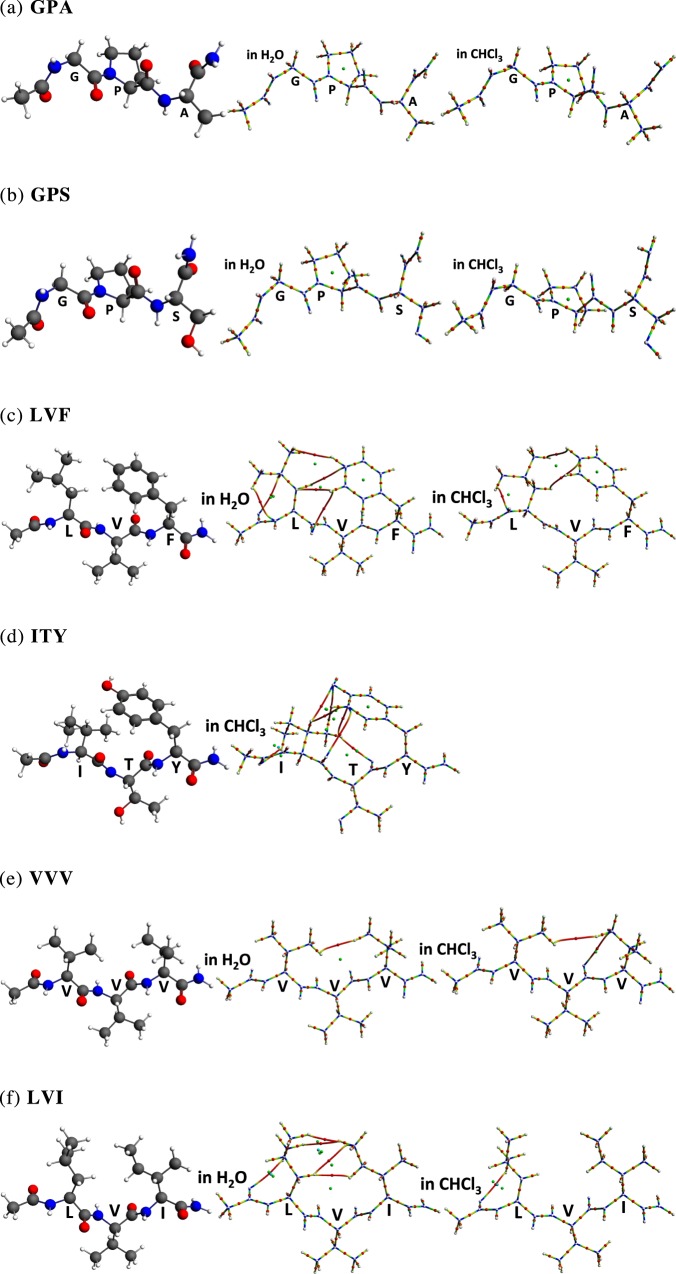
Bond paths in tri-peptides.

Tri-peptides **GPA** and **GPS** take typical PPII structures (Fig. 5a,b). In the case of **GPA** and **GPS**, no bond path was seen in water or chloroform. On the other hand, for the other β-strand-favoring sequences (**LVF**, **ITY**, **VVV**, **LVI**) (Fig. 5c–f), multiple bond paths between side chain and side chain, and between side chain and main chain are found (Fig. 5c–f). In the case of **LVF** (Fig. 5c), bond paths between side chain and main chain are detected in Leu, and the same kind of bond path is also seen in Leu dipeptide (Fig. 1). It is noteworthy that multiple bond paths including the H to H bond path, H to C bond path and C-H—π, between the benzene ring of side chain of Phe and the hydrogen or carbon atoms of the side chain of Leu were found, while the isolated Phe dipeptide did not have any bond bath (Fig. 3). Similarly, no through-space bond path is observed in the Tyr dipeptide (Supplementary Fig. 7), while in the case of **ITY** (Fig. 5d), there are multiple bond paths including the H to H bond path, H to C bond path, H to O bond path and C-H—π, between the phenol ring of side chain of Tyr (**Y**) and the hydrogen or carbon atoms of the side chain of Ile (**I**). In the case of **VVV** and **LVI** (Fig. 5e,f), we found bond paths between side chain and main chain, and also there are many H to H bond paths between the side chain and side chain. To conclude, in tripeptides, we can detect not only the side chain to main chain bond paths, which are similar to those in the dipeptide, but also multiple bond paths from side chain to side chain. This kind of side chain to side chain and side chain to main chain network of bond paths are characteristic to β-strand structures. We hypothesize that such network of bond paths may accompany with the β-strand structure in solution. Amino acids that do not show bond paths in dipeptides can form bond paths in tripeptides. Intriguingly, in tripeptides, the side chains of *i* and *i* + *2* amino acid residues tend to rotate in the same direction, i.e., N-facing (see Fig. 1). From the results of the present study of the dipeptides (e.g. Fig. 1), the N-facing of the side chain induced the formation of through-space bond path frequently in β-strand structures. Therefore, the N-facing of the side chains at the *i* and *i* + *2* amino acid residues in the β-strand structure of the tripeptide is consistent with the observation in the dipeptides.

### Conformational preferences and bond paths of amyloid β-sheet

**Figure 6:**
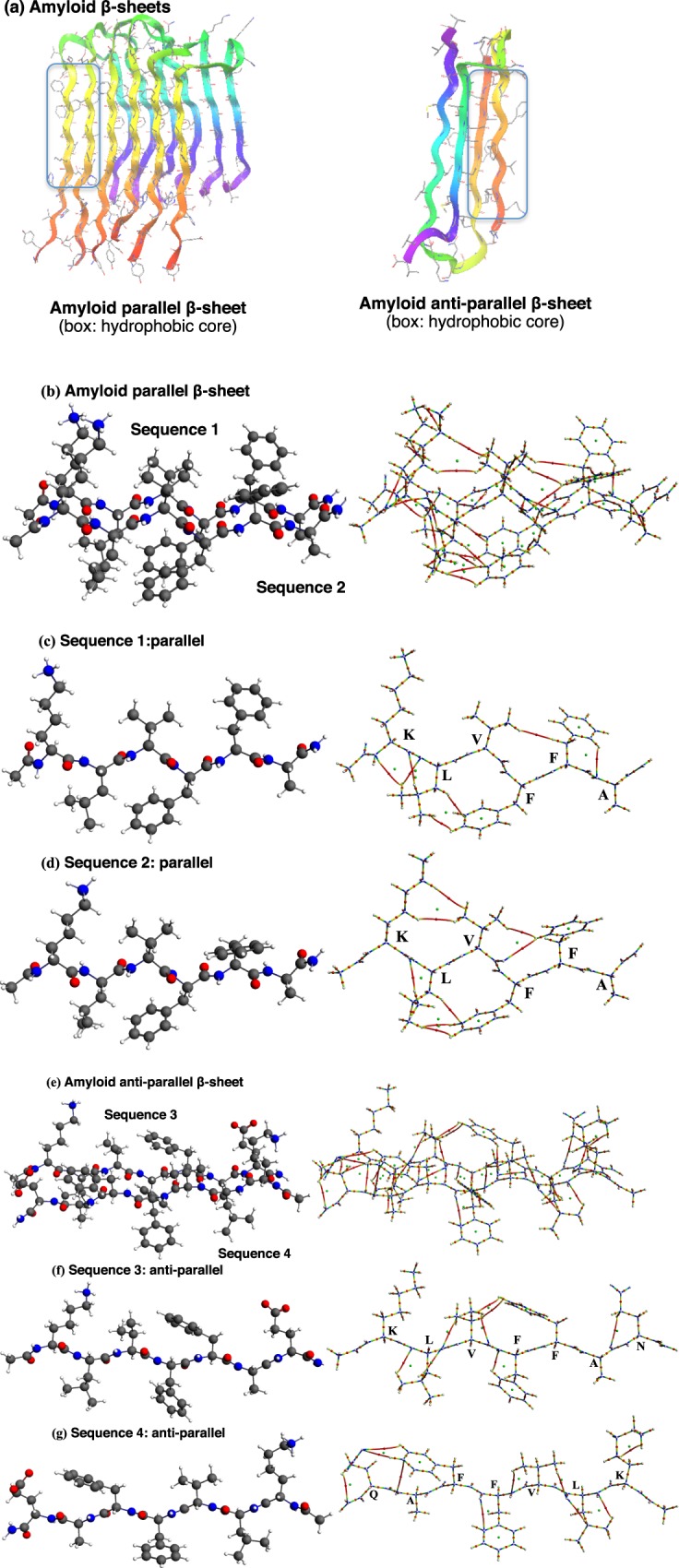
Molecular structures and molecular graphs. (**a**) Ribbon representations of amyloid parallel β-sheets and anti-parallel β-sheets. (**b**) Fragment of amyloid parallel β-sheets (**c**,**d**) a composition of parallel β-sheets, identical amino acid sequences of sequence 1 (**c**) and sequence 2 (**d**): Ac-Lys^16^-Leu^17^-Val^18^-Phe^19^-Phe^20^-Ala^21^-NH_2_. (**e**) Fragment of amyloid anti-parallel β-sheet. (**f**) Sequence 3: Ac-Lys^16^-Leu^17^-Val^18^-Phe^19^-Phe^20^-Ala^21^-Asn^22^-NH_2_; (**g**) Sequence 4: NH_2_-Glu^22^-Ala^21^-Phe^20^-Phe^19^-Val^18^-Leu^17^-Lys^16^- Ac.

β-Sheet, consisting of β-strands, is one of the most common secondary structures in proteins (Fig. 6(a)). We clipped out a parallel β-sheet sequence containing the hydrophobic aromatic core of Aβ40 (PDB 2LMP: K^16^-L^17^V^18^F^19^F^20^-A^21^)^46^ and applied QTAIM calculation directly without structure optimization. Figure 6(a) (left) shows the structure of the amyloid parallel β-sheet and Fig. 6(b) (right, molecular graph) delineates multiple inter-strand and intra-strand bond paths in the β-sheet. This complicated network contains side chain to side chain and side chain to main chain bond paths within one strand or within another strand, and also main chain to main chain bond paths between two strands. To analyze the amyloid parallel β-sheet structure further, **sequence 1** and **sequence 2** were calculated individually (Fig. 6c,d). The amino acid sequence of **sequence 1** and **sequence 2** are the same but the conformation is slightly different. The inter-strand and intra-strand interaction data are summarized in Table 2. **Sequence 1** is stabilized by one hydrogen-bonding bond path (C-H···O=C) and one hydrogen-benzene ring (C-H···π(C)) bond path^28^, and there are several peripheral weak interactions such as three hydrogen(H)-nitrogen(N) bond paths and two hydrogen(H)-hydrogen(H) bond paths, among which the hydrogen-nitrogen bond path makes the greatest contribution based on number of bond paths and accumulation of average electron density at the bond critical point. **Sequence 2** is stabilized by two hydrogen-bonding bond paths (C=O···H-C) and one hydrogen-benzene ring (C-H···π(C)) bond path, also contains four hydrogen(H)-hydrogen(H) bond paths, and one hydrogen(H)-carbon(C) bond path. Among them, the hydrogen(H)-hydrogen(H) bond path is the most abundant, based on number of bond paths. When **sequence 1** and **sequence 2** were assembled, the intra-strand interactions were conserved. As regards these inter-strand interactions, the β-sheet is stabilized by six amide hydrogen-bonding bond paths, four hydrogen-bonding bond paths (C-H···O=C), and one hydrogen-benzene ring (C-H···π(C)) bond path, also coexisting through-space bond paths such as two oxygen(O)-oxygen(O) bond paths, and ten hydrogen-hydrogen bond paths (C-H···H-C and C-H···H-N). The six amide hydrogen-bonding bond paths may contribute most to stabilization of the β-sheet based on the number of bond paths and total electron density. However, other inter-strand bond-path networks, apart from hydrogen-bonding, are characteristic of β-strand/sheet structures, and are never examined in α-helical structure^36^. The average and range of the electron density in each kind of atom pairs (Table 2) may indicate the magnitude of inter-strand interactions. In this context, the major contribution in forming the peptide assemblies comes from the conventional N-H—O=C hydrogen bonding, but the sum of various other weak interactions may be also contributed to or accompanied with overall β-strand/sheet structures.

**Table 2:** Bond type, number of bond critical points (n(BCP)), and total electron density at the bond critical points ((Σρ(r_c_)) (a.u.)) of amyloid parallel β-sheet and sequence 1 and sequence 2.

Bond type	n(BCP)	Σρ(r_c_)	Average ρ(r_c_)	Range of ρ(r_c_)
**Inter-strand interaction of Amyloid parallel β-sheet**				
N-H···O=C	6	0.1314	0.0219	0.0170–0.0284
C-H···O=C	4	0.0454	0.0114	0.0041–0.0154
C=O···O=C	2	0.0227	0.0114	0.0027–0.0200
C-H···H-C	9	0.0444	0.0049	0.0028–0.0063
C-H···π	1	0.0112	0.0112	0.0112
C-H···H-N	1	0.0080	0.0080	0.0080
**Intra-strand interaction of Sequence 1**				
C-H···O=C	1	0.0119	0.0119	0.0119
C-H···N	3	0.0369	0.0123	0.0063–0.0161
C-H···H-C	2	0.0114	0.0057	0.0023–0.0091
C-H···π	1	0.0109	0.0109	0.0109
**Intra-strand interaction of Sequence 2**				
C-H···O=C	2	0.0159	0.0080	0.0033–0.0126
C-H···H-C	4	0.0198	0.0050	0.0033–0.0060
C-H···π	1	0.0118	0.0118	0.0118
C-H···C	1	0.0039	0.0039	0.0039

Anti-parallel β-sheet was isolated from Aβ40 (PDB 2LNQ_A:^47^ K^16^-N^22^ (K^16^L^17^V^18^F^19^F^20^A^21^N^22^), 2LNQ_B: ^47^ K^16^-Q^22^ (K^16^L^17^V^18^F^19^F^20^A^21^Q^22^)) and QTAIM calculation was applied directly without structure optimization. Figure 6(e) shows the structure and bond paths (molecular graph). Various inter-strand and intra-strand bond paths were found. To analyze this complicated bond-path network, we separated the anti-parallel β-sheet, and calculated **Sequence 3** and **Sequence 4** individually (Fig. 6f,g). The inter-strand and intra-strand interaction data are collected in Supplementary Table 4. The results indicate that **Sequence 3** is stabilized by three hydrogen-bonding (C=O···H-C) bond paths and one C-H···π(C) bond path, accompanying topologically neighboring interactions such as two hydrogen(H)-nitrogen(N) bond paths, one hydrogen(H)-hydrogen(H) bond path, and one oxygen(O)-carbon(C) bond path, among which the two hydrogen-nitrogen bond paths are most characteristic in terms of the electron density. In **Sequence 4**, four hydrogen-bonding (C-H ···O=C) bond paths and two C=O···π(C) bond paths stabilize the whole structure, accompanying topologically neighboring interactions such as three hydrogen(H)-nitrogen(N) bond paths, and one hydrogen(H)-hydrogen(H) bond path, among which the three hydrogen-nitrogen bond paths are most characteristic in terms of the electron density. As for inter-strand interactions, the anti-parallel β-sheet is stabilized by seven amide hydrogen-bonding bond paths, two hydrogen-bonding bond paths (C-H···O=C), together with topologically neighboring interactions of three oxygen-oxygen bond paths, eight hydrogen-hydrogen bond paths (C-H···H-C and C-H···H-N) and one oxygen(O)-carbon(C) bond path, while the intra-strand interactions in **Sequence 3** and **Sequence 4** are conserved. Based on bond-path strength (in terms of number and average electron density at the bond critical point), the amide hydrogen-bonding bond paths are the most important (Table 3). However, other topologically neighboring inter-strand bond-path networks, apart from amide hydrogen-bonding, are also characteristic of β-strand/sheet structures. Neither amyloid parallel β-sheet nor anti-parallel β-sheet showed π-π interaction although the hydrophobic aromatic core of Aβ40 (17–20) (Leu^17^-Val^18^-Phe^19^-Phe^20^) was proposed to be important in β-sheet formation^10–15^. The QTAIM can detect through-space π-π interaction successfully in other systems^58^. Therefore the present results are consistent with the previous reports^16,17^, which excluded specific interactions involving π-electrons or aromatic character as forces that stabilize the whole fibril^10–15^.

**Table 3:** Bond type, number of bond critical points (n(BCP)), and total electron density at the bond critical points ((Σρ(r_c_)) (a.u.)) of *ant*i parallel β-sheet and sequence 3 and sequence 4.

Bond type	n(BCP)	Σρ(r_c_)	Average ρ(r_c_)	Range of ρ(r_c_)
**Inter-strand interaction of Amyloid anti-parallel β-sheet**				
N-H···O=C	7	0.2097	0.0300	0.0066–0.0505
C-H···O=C	2	0.0210	0.0105	0.0072–0.0138
C=O···O=C	3	0.0550	0.0183	0.0129–0.0286
C-H···H-C	7	0.0467	0.0067	0.0046–0.0101
C=O···C	1	0.0062	0.0062	0.0062
C-H···H-N	1	0.0043	0.0043	0.0043
**Intra-strand interaction of Sequence 3**				
C-H···O=C	3	0.0352	0.0117	0.0088–0.0140
C-H···N	2	0.0442	0.0221	0.0170–0.0272
C-H···H-C	1	0.0089	0.0089	0.0089
C-H···π	1	0.0085	0.0085	0.0085
C=O···C	1	0.0120	0.0120	0.0120
**Intra-strand interaction of Sequence 4**				
C-H···O=C	4	0.0315	0.0079	0.0027–0.0149
C=O···π	2	0.0170	0.0085	0.0030–0.0140
C-H···N	3	0.0403	0.0134	0.0117–0.0161
C-H···H-N (sp3)	1	0.0201	0.0201	0.0201

These results clearly suggest the importance of the weak bond-path network for the β-sheet structure. The van der Waals (vdW) interactions, one of the origins of hydrophobic interaction, have been regarded as dipole-induced non-directional intermolecular force, but the experiments in the crystals were inconsistent with this view. Instead, there are several reports to suggest that vdW interaction is directional, which can be described by bond path^59,60^. This notion is consistent with our present results.

In conclusion, we carried out the QTAIM analysis to examine the postulate that hydrophobic interactions in peptide assembly can be represented in more visible manner in terms of through-space bond-paths, which are weak, but local and directional. We confirmed first that amino acids bearing a branched side chain, such as Val, Ile, Thr, and Cys, and aromatic amino acids, such as Tyr, Trp and Phe, have high propensities to form β-strand structure, whereas Ala, Gly and Pro have poor propensities^19–21^, by means of the presence or absence of through-space bond path. Intriguingly, in tripeptides, the side chains of *i* and *i* + *2* amino acid residues tend to rotate in the same direction, i.e., N-facing (Fig. 1). From the results of the present study of the dipeptides, the N-facing of the side chain induced the formation of through-space bond path frequently in β-strand structures, but never induce in the PPII structures. Apart from amide hydrogen-bonding, other inter-strand bond-path networks arising from various types of weak bond paths (X-H—Y; X, Y = H, C, O, N, S), as well as *non*-H-*non*-H bond paths, are characteristic of β-strand/sheet structures. Similar bond paths from side chain to side chain and from side chain to main chain were found in a single β-strand and in di- and tripeptides. Therefore, weak interaction networks are based on a bottom-up approach from dipeptides, tripeptides to longer peptides. However, some of these through-space bond-path networks, particularly of aromatic amino acids such as Phe and Tyr were enhanced upon β-sheet formation: while there were no through-space bond paths in the Phe dipeptide and Tyr dipeptide. Multiple through-space bond paths were generated in a parallel β-sheet sequence containing the hydrophobic aromatic core of Aβ40 (K^16^-L^17^V^18^F^19^F^20^-A^21^) and anti-parallel β-sheet sequence of Aβ40 (K^16^-N^22^ (K^16^L^17^V^18^F^19^F^20^A^21^N^22^) and K^16^-Q^22^ (K^16^L^17^V^18^F^19^F^20^A^21^Q^22^)). Intriguingly, neither amyloid parallel β-sheet nor anti-parallel β-sheet showed π-π interaction between aromatic amino acids in the sequences, which was previously claimed to be significant for amyloid β-sheet formation^10–15^. Therefore the present bond-path analysis supported the previous experimental results^16,17^, which excluded specific π-π interactions as forces that stabilize the whole Aβ fibril. Thus, the present bond-path analysis may be helpful in guiding *de novo* design of bioactive Aβ mimics and binding epitopes for protein aggregation and protein-protein interaction. We already demonstrated the validity of this kind of bond bath analysis in the experimental generation of β-strand stabilizer^23^: the experimental observations were completely consistent with the through-space bond path analysis.

## Supplementary information

Supplimentary information
