# Supplementary material for: Uncovering the Networks of Topological Neighborhoods in β-Strand and Amyloid β-Sheet Structures
Source: Sci Rep. 2019 Jul 24;9:10737. doi: 10.1038/s41598-019-47151-2 (PMC6656768; doi:10.1038/s41598-019-47151-2)
Supplement: Supplementary file 1 — Supplimentary information [file 41598_2019_47151_MOESM1_ESM.pdf]

Supplementary Data

Uncovering the Networks of Topological Neighborhoods in β-Strand and Amyloid β-Sheet Structures

Luhan Zhai,[1] Yuko Otani[1] and Tomohiko Ohwada [1,*]

[1] *Laboratory of Organic and Medicinal Chemistry, Graduate School of Pharmaceutical Sciences, University of Tokyo,*
7-3-1 Hongo, Bunkyo-ku, Tokyo, 113-0033, Japan

E-mail: ohwada@mol.f.u-tokyo.ac.jp

Supplementary Table 1. Calculated torsion angles (°) of dipeptide in water and in chloroform

**Dipeptide**	Solvent	Φ (°)	Ψ (°)	χ_1(°)[a]	Free energy (au)	ΔG[b] (kcal/mol)
Ala PPII	H_2O	-58.8	143.3	-	-495.528534	-
Ala β	H_2O	-160.6	157.3	-	-495.526249	-
Ala β	$CHCl_3$	-159.9	162.4	-	-495.519306	-
Pro PPII	H_2O	-63.0	150.7	-	-572.878186	-
Leu PPII-1	H_2O	-58.7	135.9	-179.7	-613.327478	1.31
Leu PPII-2	H_2O	-59.5	144.3	-60.1	-613.329560	0.00
Leu β-1	H_2O	-156.7	131.4	-179.9	-613,326608	1.61
Leu β-2	H_2O	-141.7	141.4	-65.6	-613.329171	0.00
Leu β-1	$CHCl_3$	-155.0	138.5	-179.5	-613.322957	0.81
Leu β-2	$CHCl_3$	-138.3	151.9	-63.9	-613.324249	0.00
Leu β-3	$CHCl_3$	-161.0	165.8	75.8	-613.322027	1.39
Val PPII-2	H_2O	-61.8	155.4	-58.0	-574.061071	0.17
Val PPII-3	H_2O	-59.3	146.4	68.6	-574.061338	0.00
Val β-2	H_2O	-139.5	161.5	-59.7	-574.060633	0.00
Val β-3	H_2O	-157.3	151.2	68.8	-574.060413	0.14
Val β-2	$CHCl_3$	-136.4	166.3	-60.5	-574.056242	0.55
Val β-3	$CHCl_3$	-157.7	156.2	67.9	-574.056402	0.45
Val β-4	$CHCl_3$	-134.0	133.7	174.9	-574.057120	0.00
Ile PPII-1	H_2O	-58.5	145.5	-165.8	-613.325733	1.02
Ile PPII-2	H_2O	-59.6	136.2	-61.0	-613.326852	0.32
Ile PPII-3	H_2O	-61.8	157.6	69.5	-613.327363	0.00
Ile β-1	H_2O	-157.9	152.1	-163.7	-613.325351	0.72
Ile β-3	H_2O	-138.9	163.9	66.1	-613.326499	0.00

Ile β-1	$CHCl_3$	-156.0	154.2	-166.6	-613.323519	0.00
Ile β-2	$CHCl_3$	-135.6	133.9	-61.8	-613.323364	0.10
Ile β-3	$CHCl_3$	-136.2	166.6	66.3	-613.323099	0.26
Thr PPII-1	H_2O	-60.6	155.1	-165.6	-609.991058	1.32
Thr PPII-2	H_2O	-57.0	137.7	-59.0	-609.991951	0.76
Thr PPII-3	H_2O	-60.4	137.1	53.9	-609.993162	0.00
Thr β-1	H_2O	-158.2	164.1	-166.1	-609.990720	0.45
Thr β-2	H_2O	-160.1	136.2	-75.4	-609.991440	0.00
Thr β-1	$CHCl_3$	-157.3	178.6	-166.2	-609.982638	0.00
Thr β-2	$CHCl_3$	-161.8	143.6	-78.5	-609.980968	1.05
Thr β-3	$CHCl_3$	-139.9	168.5	64.7	-609.980668	1.24
Cys PPII-1	H_2O	-58.2	136.2	-177.7	-893.682158	0.00
Cys PPII-2	H_2O	-58.1	141.6	-62.0	-893.681263	0.56
Cys β-3	H_2O	-163.1	170.6	63.5	-893.682252	0.00
Cys β-4	H_2O	-154.7	117.0	179.5	-893.680881	0.86
Cys β-1	$CHCl_3$	-162.5	165.6	-162.3	-893.674279	0.26
Cys β-2	$CHCl_3$	-118.1	145.7	-64.8	-893.674687	4.74
Phe PPII-1	H_2O	-59.4	141.1	-179.9	-726.418594	0.00
Phe PPII-2	H_2O	-58.8	142.1	-62.6	-726.417264	0.83
Phe PPII-3	H_2O	-61.2	155.8	64.9	-726.416991	1.01
Phe β-3	H_2O	-167.8	162.0	59.7	-726.416245	0.38
Phe β-4	H_2O	-158.3	139.5	179.4	-726.416857	0.00
Phe β-1	$CHCl_3$	-159.6	147.5	-176.7	-726.416969	0.00
Phe β-3	$CHCl_3$	-162.9	168.1	61.3	-726.415144	1.14
Tyr PPII-2	H_2O	-58.2	142.8	-62.9	-801.619718	0.76
Tyr PPII-4	H_2O	-58.5	141.4	175.5	-801.620925	0.00
Tyr β-1	H_2O	-159.4	143.5	-177.6	-801.620569	0.00
Tyr β-3	H_2O	-167.5	160.8	59.6	-801.620141-	0.27
Tyr β-1	$CHCl_3$	-158.9	147.3	-175.3	801.612059	1.05
Tyr β-2	$CHCl_3$	-141.6	148.3	-57.8	-801.610413	2.08
Tyr β-3	$CHCl_3$	-163.4	167.8	58.9	-801.613730	0.00
Trp PPII-2	H_2O	-57.3	142.1	-61.3	-857.926156	0.90
Trp PPII-3	H_2O	-61.3	156.2	61.9	-857.924689	1.82
Trp PPII-4	H_2O	-57.9	144.2	176.9	-857.927596	0.00
Trp β-1	H_2O	-158.5	138.1	-179.6	-857.926898	0.00
Trp β-2	H_2O	-148.9	140.5	-54.5	-857.925387	0.95
Trp β-3	H_2O	-164.3	164.1	62.5	-857.925635	0.79
Trp β-4	H_2O	-160.0	144.9	179.0	-857.926674	0.14
Trp PPII-3	$CHCl_3$	-73.6	167.2	60.4	-857.919693	-
Trp β-1	$CHCl_3$	-160.3	152.3	-174.2	-857.923477	0.00
Trp β-3	$CHCl_3$	-161.8	170.8	62.4	-857.920905	1.61

a) χ_1: dihedral angle of the side chain, N-Cα-Cβ-Xγ (X=heavy atom at the γ position).

b) Relative energy (in kcal/mol) among the conformations of similar secondary structure in the same solvent.

Additional data of dipeptides

Ala dipeptide Ala has been regarded as a β-strand-disfavoring amino acid.[1] After conformational search and DFT optimization, one PPII structure is obtained in Ala dipeptide (Ac-Ala-NHMe) in water and no structure corresponding to the PPII structure is obtained in chloroform. One β-strand structure is obtained in water and in chloroform respectively. Among these three conformations, no bond path was found (Supplementary Figure 1).

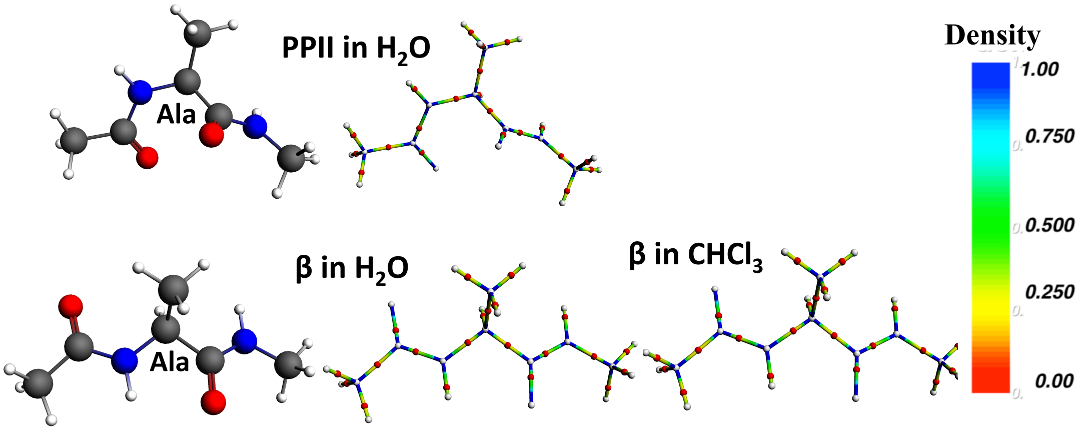

Supplementary Figure 1. Ala dipeptide (Ac-Ala-NHMe) in water and in chloroform. In the ball-and-stick model, the colors have the following meaning: red ball, oxygen; blue ball, nitrogen; dark grey ball, carbon; white ball, hydrogen.

Pro dipeptide In the case of Pro dipeptide, we obtained only a PPII structure in water, and no bond path was detected (Supplementary Figure 2).

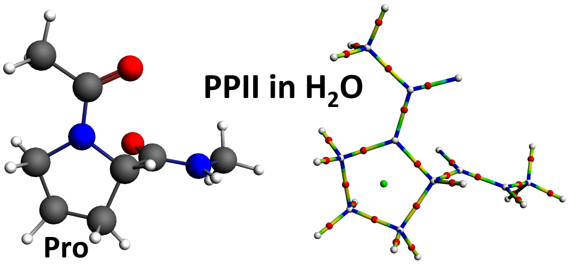

Supplementary Figure 2. Pro dipeptide (Ac-Pro-NHMe) in water.

Val dipeptide In the case of Val, two PPII structures with different side chain directions (PPII-2 and PPII-3) are obtained in water (Supplementary Figure 3(a)). In center-facing PPII-3, one bond path between methyl-H of the side chain and the main chain N is detected (Supplementary Figure 3(a)), while in the N-facing PPII-2, there was no bond path. Two β-strand structures with different side chain directions (β-2

and β-3) are obtained in water (Supplementary Figure 3(b)). In the center-facing β-3 structure in water, the side chain is rotated to the center of Val and there is a bond path between methyl-H of the side chain and the main chain N. In the N-facing β-2 structure in water, there was no bond path. Three β-strand structures with different side chain directions (β-2, β-3 and β-4) are obtained in chloroform (Supplementary Figure 3(c)).

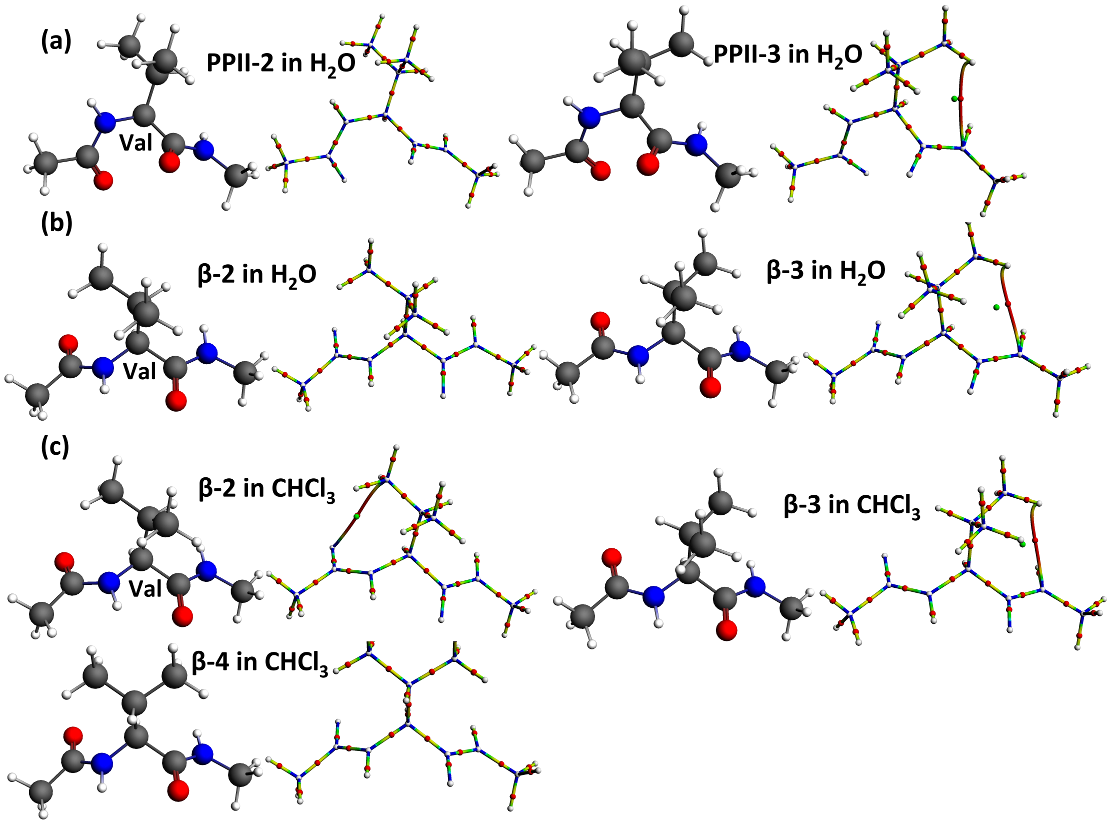

Supplementary Figure 3. Val dipeptide (Ac-Val-NHMe) in water and in chloroform.

In the N-facing β-2 structure in chloroform with the side chain rotating to the N-terminal side, a bond path between carbon of the side chain and main chain carbonyl oxygen is detected. In the center facing β-3 in chloroform, when the side chain is rotated to the middle, there is a bond path between methyl-H of the side chain and the main chain N. In the C'-facing β-4 in chloroform, when the side chain is rotated to the C-terminal side, there is no bond path.

Ile dipeptide In the case of Ile, three PPII structures with different side chain directions (PPII-1, PPII-2 and PPII-3) are obtained in water (Supplementary Figure

4(a)). In C-facing PPII-1, one bond path between methyl-H of the side chain and the main chain N-H is detected (Supplementary Figure 4(a)). In the N-facing PPII-2 and center-facing PPII-3, there is no bond path. Two β-strand structures with different side chain directions (β-1 and β-3) are obtained in water (Supplementary Figure 4(b). In C-facing β-1 in water (Supplementary Figure 4(b)), when the side chain is rotated to the C-terminal side, there are two bond paths.

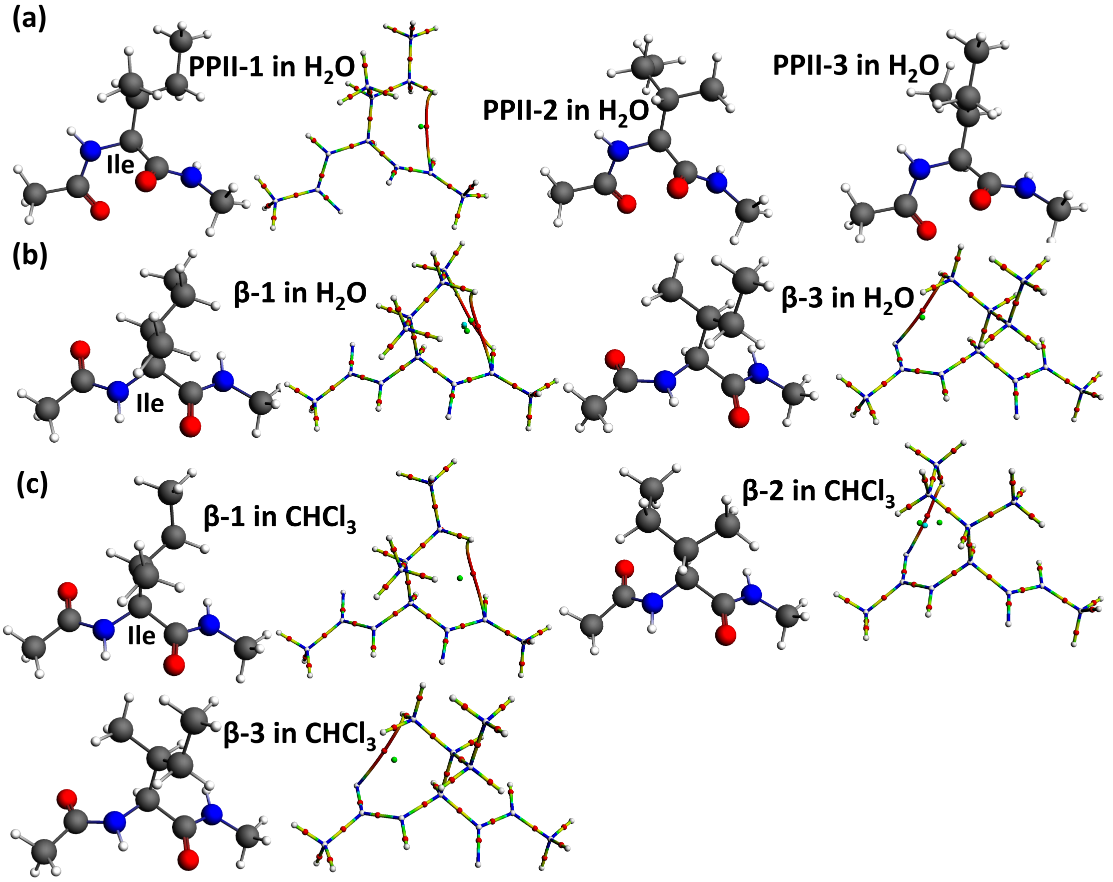

Supplementary Figure 4. Ile dipeptide (Ac-Ile-NHMe) in water and in chloroform

One bond path lies between methyl-H of the side chain and the main chain N-H and the other lies between methyl-H of the side chain and the main chain N. In center-facing β-3 in water (Supplementary Figure 4(b)), the side chain is rotated to the center and there is a bond path between methyl-H of the side chain and the acetyl carbonyl oxygen. Three β-strand structures (β-1, β-2 and β-3) appear in chloroform (Supplementary Figure 4 (c)). In N-facing β-2 and center-facing β-3 structures in chloroform, bond path between methyl-H of the side chain and the acetyl carbonyl oxygen is found. In C-facing β-1 structure in chloroform (Supplementary Figure 4(c)),

when the side chain is rotated to the C-terminal, there is a bond path between methyl-H of the side chain and the main chain N.

Thr dipeptide In the case of Thr, three PPII structures with different side chain directions (PPII-1, PPII-2 and PPII-3) are obtained in water (Supplementary Figure 5 (a)). In the center-facing PPII-3 structure in water, one bond path between hydroxyl-H of the side chain and the main chain carbonyl oxygen is detected, while no bond paths are found in the C-facing PPII-1 and N-facing PPII-2 structures (Supplementary Figure 5(a)).

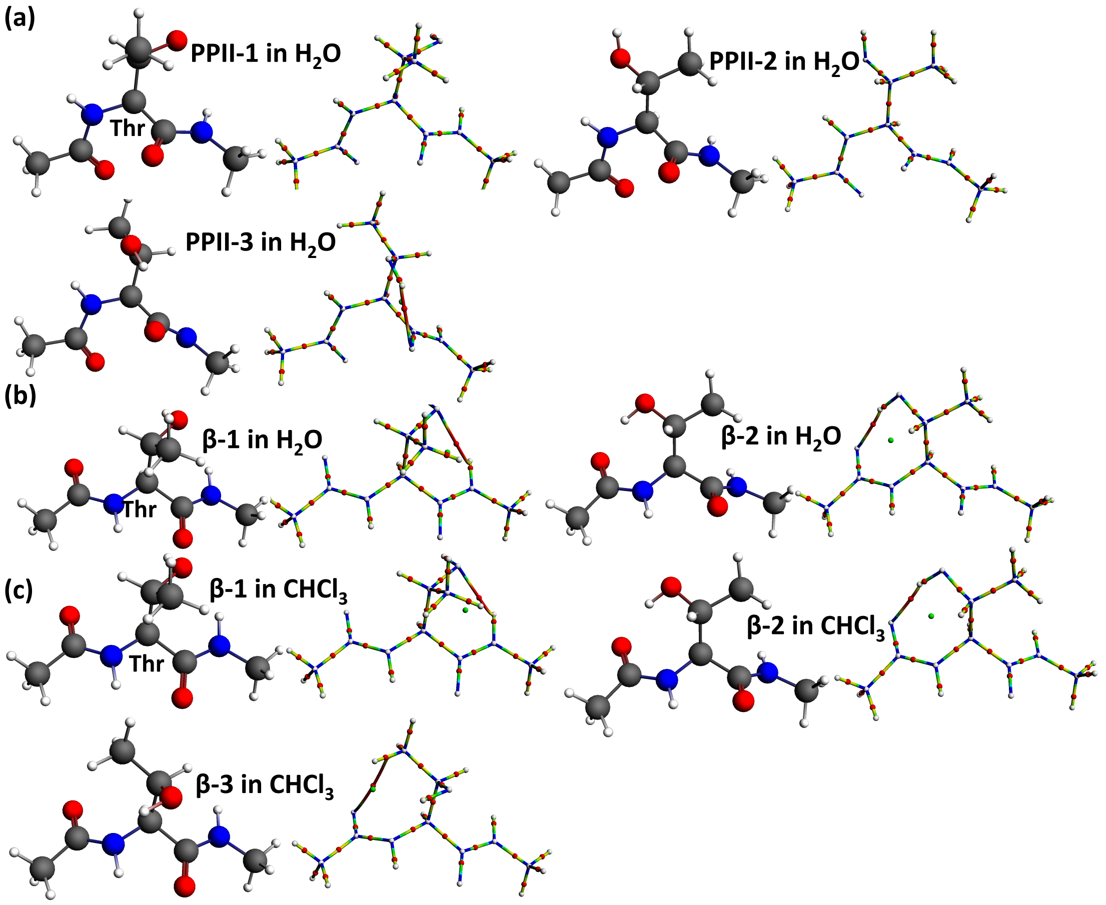

Supplementary Figure 5. Thr dipeptide (Ac-Thr-NHMe) in water and in chloroform

Two β-strand structures with different side chain directions (β-1 and β-2) are obtained in water and three β-strand structures (β-1, β-2 and β-3) appear in chloroform (Supplementary Figure 5 (b) and (c)). In C-facing β-1 structures in water and chloroform (Supplementary Figure 5(b) and (c)), when the hydroxyl group of side chain is rotated to the C-terminal side, there is a bond path between hydroxy-O of the

side chain and the main chain (N-)H. In N-facing β-2 structures in water and chloroform (Supplementary Figure 5(b) and (c)), the hydroxyl group of the side chain is rotated to the N-terminal side of Thr and there is a bond path between the hydroxy-H of the side chain and the main chain acetyl carbonyl oxygen. In center-facing β-3 structure in chloroform (Supplementary Figure 5(c)), when the hydroxyl group of side chain is rotated to the middle, one bond path between the methyl-H of the side chain and main chain acetyl carbonyl oxygen (O) appears.

Cys dipeptide In the case of Cys, two PPII structures (PPII-1 and PPII-2) with the side chain thiol group directed to the C- and N-terminal are obtained in water (Supplementary Figure 6 (a)).

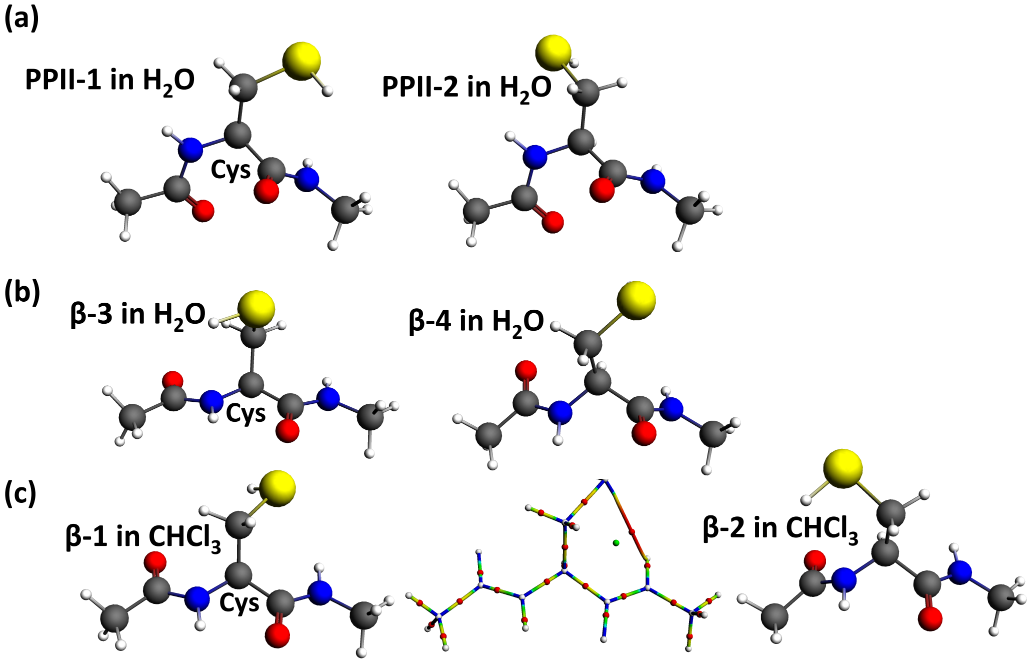

Supplementary Figure 6. Cys dipeptide (Ac-Cys-NHMe) in water and in chloroform

These contain no bond path. Two β-strand structures (β-3 and β-4) with the thiol group of the side chain rotated to the middle and C-terminal side are obtained in water (Supplementary Figure 6 (b)), and these also contain no bond path. Two β-strand structures (β-1 and β-2) with the thiol group of the side chain rotated to the C-terminal and N-terminal side are obtained in chloroform (Supplementary Figure 6 (c)). Only in the C-facing β-1 structure in chloroform do we find one bond path between thiol-S of the side chain and the main chain N-H (Supplementary Figure 6(c)).

Tyr dipeptide For the aromatic amino acid Tyr dipeptide, two PPII structures (PPII-2 and PPII-4) with the side chain directed to the N-terminal and C-terminal are obtained in water (Supplementary Figure 7 (a)). Two β-strand structures (β-1 and β-3) with the side chain rotated to the C-terminal side and middle are obtained in water (Supplementary Figure 7 (b)). Three β-strand structures (β-1, β-2 and β-3) with the direction of side chain pointed to the C-terminal, N-terminal and middle are formed in chloroform (Supplementary Figure 7 (c)). No bond path is detected in any of these structures.

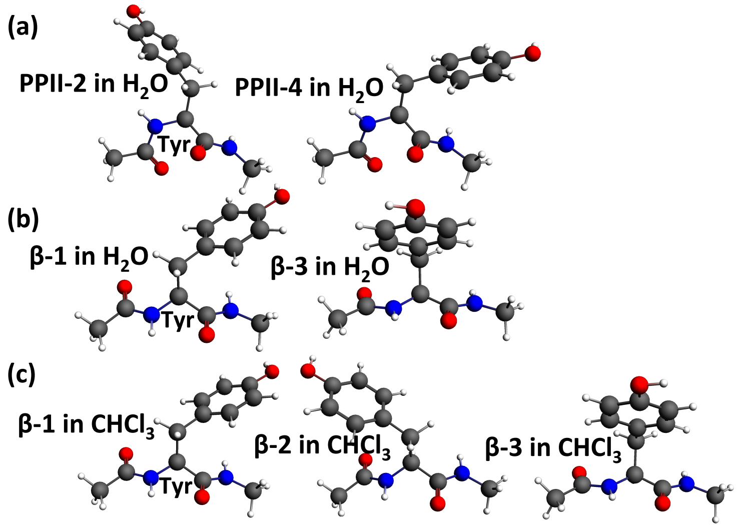

Supplementary Figure 7. Aromatic dipeptides, Tyr dipeptide in water and in chloroform.

Supplementary Figure 8.
Superposition of tripeptides structures obtained by PDB crystal structure database and by DFT geometry optimization

LVF

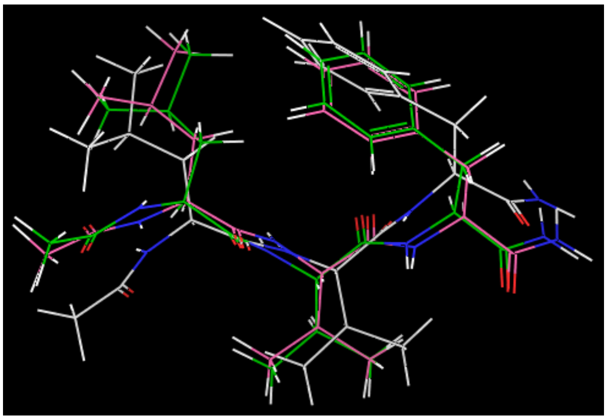

Crystal structure (grey)
M06-2X/6-31+G(d), scrf=(smd, solvent=chloroform) optimized structure (green)
M06-2X/6-31+G(d), scrf=(smd, solvent=water) optimized structure (pink)

ITY

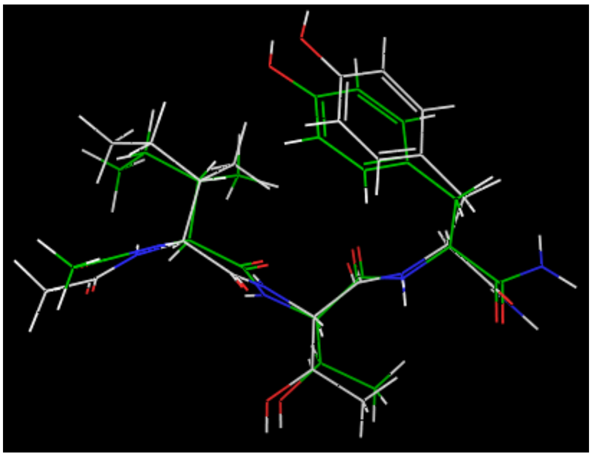

Crystal structure (grey)
M06-2X/6-31+G(d), scrf=(smd, solvent=chloroform) optimized structure (green)

VVV

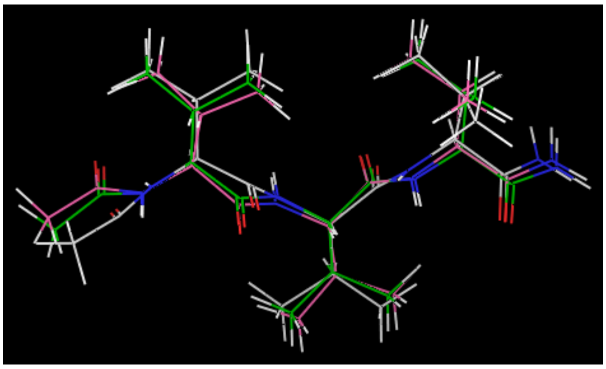

Crystal structure (grey)
M06-2X/6-31+G(d), scrf=(smd, solvent=chloroform) optimized structure (green)

M06-2X/6-31+G(d), scrf=(smd, solvent=water) optimized structure (pink)
LVI

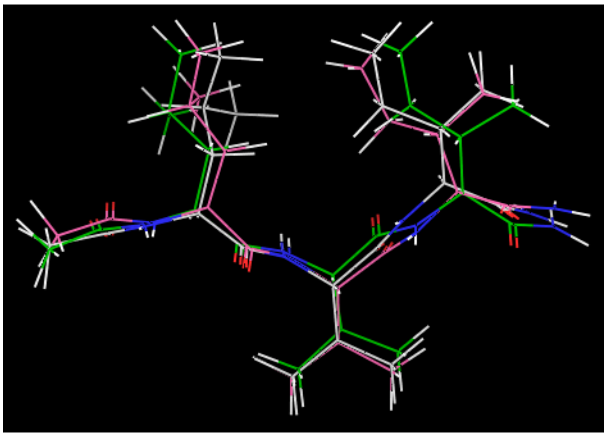

Crystal structure (grey)
M06-2X/6-31+G(d), scrf=(smd, solvent=chloroform) optimized structure (green)
M06-2X/6-31+G(d), scrf=(smd, solvent=water) optimized structure (pink)

Supplementary Table 2 Torsion angles (°) of GPA, GPS, LVF, ITY, VVV and LVI in water and chloroform

**(a)**	**Gly**		**Pro**		**Ala**	
	φ	ψ	φ	ψ	φ	ψ
Water<br>Chlorofom	-66.1<br>-69.6	166.8<br>164.4	-62.8<br>-69.0	156.3<br>147.9	-55.3<br>-62.0	148.5<br>144.9
**(b)**	**Gly**		**Pro**		**Ser**	
	φ	ψ	φ	ψ	φ	ψ
Water<br>Chlorofom	-67.0<br>-71.1	167.1<br>166.2	-62.3<br>-67.9	151.4<br>152.3	-56.4<br>-56.4	147.8<br>139.6
**(c)**	**Leu**		**Val**		**Phe**	
	φ	ψ	φ	ψ	φ	ψ
Water<br>Chloroform	-139.4<br>-110.5	143.0<br>154.2	-126.3<br>-114.3	141.9<br>138.5	-139.9<br>-130.0	153.7<br>150.0
**(d)**[a]	**Ile**		**Thr**		**Tyr**	
	φ	ψ	φ	ψ	φ	ψ
Chloroform	-136.6	140.2	-81.7	134.8	-131.1	155.0
**(e)**	**Val**		**Val**		**Val**	
	φ	ψ	φ	ψ	φ	ψ
Water<br>Chloroform	-139.1<br>-133.7	138.8<br>133.8	-127.0<br>-133.0	137.1<br>135.4	-133.7<br>-136.9	168.6<br>167.8
**(f)**	**Leu**		**Val**		**Ile**	
	φ	ψ	φ	ψ	φ	ψ
Water<br>Chloroform	-141.0<br>-139.0	147.1<br>156.1	-131.2<br>-127.7	111.1<br>133.8	-139.9<br>-131.1	129.4<br>134.2

a) The structure of **ITY** was changed in water.

Supplementary Table 3 Torsion angles (°) of a single β-strand

Phe		Arg		Val		Val		Val	
φ	ψ	φ	ψ	φ	ψ	φ	ψ	φ	ψ
-96.7	141.4	-131.1	142.9	-106.3	123.6	-110.0	109.2	-93.7	144.0

Additional data

Conformational preferences and bond paths of a single β-strand

In the previous section, all the structures were calculated in the solution state. Here, we clipped out the structure of a single β-strand from the crystal structure of the whole protein and directly submitted it to QTAIM calculation without further structure optimization. The single strand structure was taken from F^{64}-V^{68} of PDB entry 1EST.[2] Selected main chain torsion angles are shown in Supplementary Table 3. As shown in Supplementary Figure 9, we found multiple bond paths from side chain to side chain, and from side chain to main chain in this single β-strand structure.

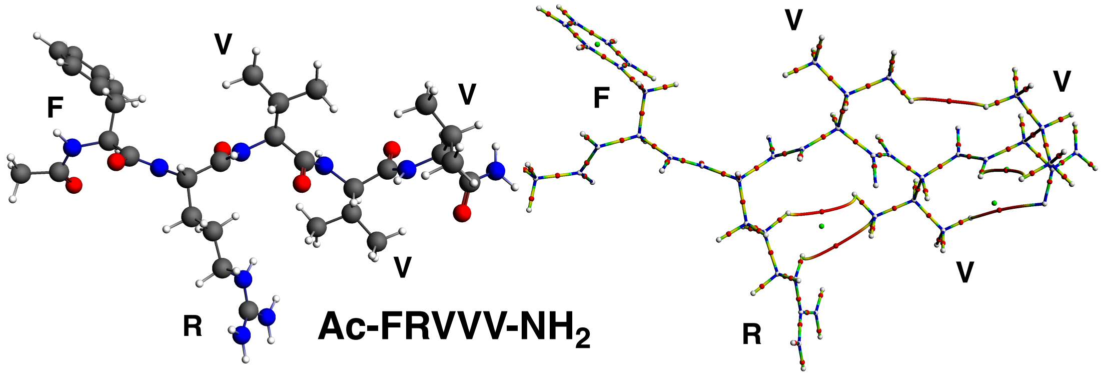

Supplementary Figure 9. Example of a single β-strand sequence, Ac-F-R-V-V-V-NH_2.

Calculation Coordinates, Frequency and Thermal Values.[3]

Ac-Ala-NHMe PPII: M06-2X/6-31+G(d), scrf=(smd, solvent=water)

NIMG=1 (37.7876 cm^{-1})

```
Zero-point correction=                           0.188262 (Hartree/Particle)
 Thermal correction to Energy=                     0.200296
 Thermal correction to Enthalpy=                   0.201240
 Thermal correction to Gibbs Free Energy=          0.149386
 Sum of electronic and zero-point Energies=           -495.489659
 Sum of electronic and thermal Energies=              -495.477625
 Sum of electronic and thermal Enthalpies=            -495.476681
 Sum of electronic and thermal Free Energies=         -495.528534
Standard orientation:
```

Center Number	Atomic Number	Atomic Type	Coordinates (Angstroms) X	Y	Z
1	6	0	-3.271855	-0.682882	0.335799
2	6	0	-1.877969	-0.505733	-0.185315
3	8	0	-1.268779	-1.420592	-0.752275
4	1	0	-3.283973	-1.525038	1.037125
5	1	0	-3.664834	0.207851	0.835918
6	1	0	-3.931599	-0.945610	-0.499412
7	7	0	-1.320487	0.711162	-0.015287
8	6	0	0.032789	0.988862	-0.450808
9	6	0	1.024987	0.053267	0.235282
10	8	0	0.920598	-0.242438	1.428921
11	6	0	0.390674	2.421226	-0.096015
12	1	0	-1.823051	1.428804	0.495159
13	1	0	0.095906	0.845056	-1.539532
14	1	0	-0.309427	3.122048	-0.565777
15	1	0	0.351192	2.562208	0.992184
16	1	0	1.402825	2.660652	-0.440224
17	7	0	2.051776	-0.354914	-0.528646
18	6	0	3.123531	-1.162266	0.015488
19	1	0	2.072525	-0.099172	-1.508441
20	1	0	3.617317	-0.649268	0.849440
21	1	0	2.746501	-2.125353	0.380210
22	1	0	3.860094	-1.346518	-0.768877

Ac-Ala-NHMe β: M06-2X/6-31+G(d), scrf=(smd, solvent=water)

NIMG=1 (39.6250 cm^{-1})

```
Zero-point correction=                           0.188175 (Hartree/Particle)
 Thermal correction to Energy=                     0.200070
 Thermal correction to Enthalpy=                   0.201014
 Thermal correction to Gibbs Free Energy=          0.149780
 Sum of electronic and zero-point Energies=           -495.487854
 Sum of electronic and thermal Energies=              -495.475959
 Sum of electronic and thermal Enthalpies=            -495.475015
 Sum of electronic and thermal Free Energies=         -495.526249
Standard orientation:
```

Center Number	Atomic Number	Atomic Type	Coordinates (Angstroms) X	Y	Z
1	6	0	-3.354723	-1.144710	0.057580

2	6	0	-2.319934	-0.030643	-0.234572
3	8	0	-2.519071	0.888730	-1.025486
4	1	0	-3.641892	-1.563652	-0.907011
5	1	0	-2.979021	-1.895814	0.752069
6	1	0	-4.294366	-0.776501	0.469786
7	7	0	-1.101583	-0.214826	0.276238
8	6	0	0.055775	0.592561	0.035087
9	6	0	1.303976	-0.302449	0.100712
10	8	0	1.282790	-1.377819	0.715813
11	6	0	0.208299	1.756730	1.007947
12	1	0	-0.884264	-0.939620	0.944035
13	1	0	0.037131	0.868509	-1.019233
14	1	0	-0.361941	2.624515	0.676884
15	1	0	-0.244827	1.468683	1.957526
16	1	0	1.224717	2.106479	1.186359
17	7	0	2.410014	0.226013	-0.506106
18	6	0	3.672277	-0.445111	-0.519438
19	1	0	2.273985	1.091829	-1.008257
20	1	0	4.356656	0.087900	0.141335
21	1	0	3.707850	-1.512499	-0.301603
22	1	0	4.143180	-0.283684	-1.489325

Ac-Ala-NHMe β: M06-2X/6-31+G(d), scrf=(smd, solvent=chloroform)

```
NIMG=1 (45.1074 cm-1)
Zero-point correction=                      0.188358 (Hartree/Particle)
 Thermal correction to Energy=                0.200282
 Thermal correction to Enthalpy=              0.201226
 Thermal correction to Gibbs Free Energy=     0.150004
 Sum of electronic and zero-point Energies=       -495.480953
 Sum of electronic and thermal Energies=          -495.469029
 Sum of electronic and thermal Enthalpies=        -495.468084
 Sum of electronic and thermal Free Energies=     -495.519306
Standard orientation:
```

Center Number	Atomic Number	Atomic Type	Coordinates (Angstroms) X	Y	Z
1	6	0	-3.354723	-1.144710	0.057580
2	6	0	-2.319934	-0.030643	-0.234572
3	8	0	-2.519071	0.888730	-1.025486
4	1	0	-3.641892	-1.563652	-0.907011
5	1	0	-2.979021	-1.895814	0.752069
6	1	0	-4.294366	-0.776501	0.469786
7	7	0	-1.101583	-0.214826	0.276238
8	6	0	0.055775	0.592561	0.035087
9	6	0	1.303976	-0.302449	0.100712
10	8	0	1.282790	-1.377819	0.715813
11	6	0	0.208299	1.756730	1.007947
12	1	0	-0.884264	-0.939620	0.944035
13	1	0	0.037131	0.868509	-1.019233
14	1	0	-0.361941	2.624515	0.676884
15	1	0	-0.244827	1.468683	1.957526
16	1	0	1.224717	2.106479	1.186359
17	7	0	2.410014	0.226013	-0.506106
18	6	0	3.672277	-0.445111	-0.519438
19	1	0	2.273985	1.091829	-1.008257
20	1	0	4.356656	0.087900	0.141335
21	1	0	3.707850	-1.512499	-0.301603

22	1	0	4.143180	-0.283684	-1.489325

Ac-Pro-NHMe PPII: M06-2X/6-31+G(d), scrf=(smd, solvent=water)

```
NIMG=1 (36.3316 cm-1)
Zero-point correction=                           0.225231 (Hartree/Particle)
 Thermal correction to Energy=                    0.237987
 Thermal correction to Enthalpy=                  0.238931
 Thermal correction to Gibbs Free Energy=         0.185376
 Sum of electronic and zero-point Energies=          -572.838331
 Sum of electronic and thermal Energies=             -572.825575
 Sum of electronic and thermal Enthalpies=           -572.824631
 Sum of electronic and thermal Free Energies=        -572.878186
Standard orientation:
```

Center Number	Atomic Number	Atomic Type	Coordinates (Angstroms) X	Y	Z
1	6	0	-2.696498	-1.807873	0.414064
2	6	0	-1.383419	-1.342590	-0.172789
3	8	0	-0.621888	-2.179520	-0.725552
4	1	0	-2.617393	-2.151747	1.445183
5	1	0	-3.546288	-1.129947	0.324995
6	1	0	-3.177342	-2.629220	-0.116065
7	7	0	-1.086271	0.010074	-0.148520
8	6	0	0.151397	0.556085	-0.730770
9	6	0	1.357763	0.182595	0.192108
10	8	0	1.314769	0.339342	1.414453
11	6	0	-1.897637	0.998476	0.447032
12	6	0	-0.049513	2.084022	-0.809596
13	6	0	-1.198985	2.380099	0.167612
14	1	0	-2.946898	0.994977	0.150170
15	1	0	-1.894812	0.770520	1.513280
16	1	0	0.372469	0.196594	-1.736093
17	1	0	0.805678	2.688583	-0.507198
18	1	0	-0.362735	2.413882	-1.800429
19	1	0	-1.844515	3.147207	-0.261664
20	1	0	-0.684163	2.782758	1.039682
21	7	0	2.394172	-0.398296	-0.430089
22	6	0	3.575561	-0.797608	0.266649
23	1	0	2.387453	-0.581013	-1.422699
24	1	0	3.292365	-1.422367	1.114148
25	1	0	4.293162	-1.294588	-0.385852
26	1	0	4.072648	0.134094	0.535735

Ac-Leu-NHMe PPII-2: M06-2X/6-31+G(d), scrf=(smd, solvent=water)

```
NIMG=1 (34.6487 cm-1)
Zero-point correction=                           0.274041 (Hartree/Particle)
 Thermal correction to Energy=                    0.290061
 Thermal correction to Enthalpy=                  0.291005
 Thermal correction to Gibbs Free Energy=         0.229490
 Sum of electronic and zero-point Energies=          -613.285009
 Sum of electronic and thermal Energies=             -613.268989
 Sum of electronic and thermal Enthalpies=           -613.268045
 Sum of electronic and thermal Free Energies=        -613.329560

Standard orientation:
 ---------------------------------------------------------------------
 Center     Atomic      Atomic             Coordinates (Angstroms)
```

Number	Number	Type	X	Y	Z
1	6	0	0.667911	3.596333	-0.457130
2	6	0	0.738942	2.227564	0.204018
3	8	0	1.411369	2.076784	1.225866
4	7	0	0.044405	1.235127	-0.367020
5	6	0	0.045647	-0.155968	0.093084
6	6	0	1.346205	-0.869635	-0.318773
7	8	0	1.920040	-0.552308	-1.363238
8	6	0	-1.181564	-0.895488	-0.493258
9	6	0	-2.554939	-0.342666	-0.042682
10	6	0	-3.705235	-1.030355	-0.809665
11	6	0	-2.748610	-0.419848	1.487296
12	7	0	1.763099	-1.846508	0.492035
13	6	0	2.958813	-2.657416	0.274421
14	1	0	0.028099	3.590112	-1.339523
15	1	0	0.271921	4.331610	0.242891
16	1	0	1.663460	3.917240	-0.762816
17	1	0	-0.457173	1.413345	-1.225080
18	1	0	-0.013457	-0.160063	1.183209
19	1	0	-1.116798	-0.870968	-1.583027
20	1	0	-1.125399	-1.952556	-0.225077
21	1	0	-2.595471	0.712583	-0.318779
22	1	0	-4.342326	-1.636572	-0.165224
23	1	0	-4.346350	-0.291126	-1.291696
24	1	0	-3.339208	-1.692405	-1.595461
25	1	0	-2.600721	0.560207	1.942884
26	1	0	-3.748075	-0.749318	1.771990
27	1	0	-2.043184	-1.105201	1.958029
28	1	0	1.221984	-2.048114	1.324067
29	1	0	2.686893	-3.709469	0.186417
30	1	0	3.511916	-2.381913	-0.624998
31	1	0	3.637075	-2.558662	1.122201

Ac-Leu-NHMe PPII-1: M06-2X/6-31+G(d), scrf=(smd, solvent=water)

```
NIMG=1 (50.7719 cm-1)
Zero-point correction=                           0.274709 (Hartree/Particle)
 Thermal correction to Energy=                    0.290353
 Thermal correction to Enthalpy=                  0.291297
 Thermal correction to Gibbs Free Energy=         0.231709
 Sum of electronic and zero-point Energies=          -613.284478
 Sum of electronic and thermal Energies=             -613.268835
 Sum of electronic and thermal Enthalpies=           -613.267890
 Sum of electronic and thermal Free Energies=        -613.327478
Standard orientation:
```

Center Number	Atomic Number	Atomic Type	Coordinates (Angstroms) X	Y	Z
1	6	0	-3.839882	-1.401375	-0.262151
2	6	0	-2.616342	-0.698684	0.307573
3	8	0	-2.745386	0.083955	1.250848
4	7	0	-1.431741	-0.978979	-0.252141
5	6	0	-0.162878	-0.345362	0.114420
6	6	0	-0.103951	1.093308	-0.434710
7	8	0	-0.531404	1.332662	-1.566162
8	6	0	0.998795	-1.225969	-0.414090
9	6	0	2.427474	-0.781082	-0.015207
10	6	0	2.631133	-0.750546	1.515506
11	6	0	3.491268	-1.645705	-0.726333
12	7	0	0.444632	2.013341	0.364229
13	6	0	0.609676	3.423690	0.022429
14	1	0	-3.580343	-2.072955	-1.080550

15	1	0	-4.331612	-1.987558	0.513727
16	1	0	-4.552188	-0.667779	-0.638914
17	1	0	-1.400102	-1.600463	-1.047899
18	1	0	-0.114945	-0.308926	1.204451
19	1	0	0.850867	-2.252685	-0.075004
20	1	0	0.935056	-1.260353	-1.503671
21	1	0	2.576703	0.235865	-0.382287
22	1	0	3.577925	-1.192927	1.826286
23	1	0	2.632290	0.275749	1.884318
24	1	0	1.844463	-1.286149	2.047679
25	1	0	4.200740	-1.019368	-1.268819
26	1	0	4.069114	-2.261858	-0.037060
27	1	0	3.049633	-2.326768	-1.454816
28	1	0	0.773761	1.724055	1.276783
29	1	0	0.069470	4.047412	0.734983
30	1	0	1.663477	3.698902	0.070624
31	1	0	0.248012	3.672683	-0.976557

Ac-Leu-NHMe β-2: M06-2X/6-31+G(d), scrf=(smd, solvent=water)

NIMG=1 (28.2303 cm^{-1})
Zero-point correction= 0.273470 (Hartree/Particle)
Thermal correction to Energy= 0.289648
Thermal correction to Enthalpy= 0.290592
Thermal correction to Gibbs Free Energy= 0.228398
Sum of electronic and zero-point Energies= -613.284099
Sum of electronic and thermal Energies= -613.267921
Sum of electronic and thermal Enthalpies= -613.266977
Sum of electronic and thermal Free Energies= -613.329171
Standard orientation:

Center Number	Atomic Number	Atomic Type	Coordinates (Angstroms) X	Y	Z
1	6	0	-1.384359	3.353401	-0.564507
2	6	0	-0.873425	2.153665	0.219891
3	8	0	-0.980833	2.138466	1.447311
4	7	0	-0.322267	1.157136	-0.482275
5	6	0	0.282062	-0.050016	0.084180
6	6	0	1.780034	-0.058071	-0.256243
7	8	0	2.184536	0.517107	-1.269731
8	6	0	-0.406544	-1.302658	-0.512504
9	6	0	-1.901384	-1.462314	-0.147224
10	6	0	-2.539035	-2.632873	-0.926450
11	6	0	-2.126305	-1.585918	1.375324
12	7	0	2.570963	-0.733896	0.581984
13	6	0	4.017151	-0.876653	0.430495
14	1	0	-1.230538	3.234787	-1.637061
15	1	0	-2.451113	3.489640	-0.388716
16	1	0	-0.867374	4.258213	-0.246007
17	1	0	-0.191275	1.265936	-1.479080
18	1	0	0.185406	-0.057891	1.171368
19	1	0	-0.297599	-1.278197	-1.598695
20	1	0	0.129401	-2.197044	-0.188172
21	1	0	-2.419253	-0.559935	-0.477294
22	1	0	-2.849274	-3.454190	-0.279998
23	1	0	-3.423991	-2.299986	-1.470316
24	1	0	-1.856347	-3.058447	-1.662901
25	1	0	-2.490612	-0.643398	1.786399
26	1	0	-2.862784	-2.346613	1.635330
27	1	0	-1.208823	-1.837110	1.908207
28	1	0	2.150978	-1.184980	1.385641
29	1	0	4.413422	-0.368682	-0.450283
30	1	0	4.527222	-0.469685	1.303895

```
   31        1         0       4.282881   -1.931059    0.351315
 ---------------------------------------------------------------------
```

Ac-Leu-NHMe β-1: M06-2X/6-31+G(d), scrf=(smd, solvent=water)

```
NIMG=1 (40.9869 cm-1)
Zero-point correction=                           0.274396 (Hartree/Particle)
 Thermal correction to Energy=                     0.290109
 Thermal correction to Enthalpy=                   0.291053
 Thermal correction to Gibbs Free Energy=          0.230970
 Sum of electronic and zero-point Energies=          -613.283182
 Sum of electronic and thermal Energies=             -613.267469
 Sum of electronic and thermal Enthalpies=           -613.266525
 Sum of electronic and thermal Free Energies=        -613.326608
Standard orientation:
 ---------------------------------------------------------------------
 Center     Atomic      Atomic             Coordinates (Angstroms)
 Number     Number       Type             X           Y           Z
 ---------------------------------------------------------------------
    1          6           0       -4.092453    0.113723   -0.304291
    2          6           0       -2.728778   -0.264678    0.254855
    3          8           0       -2.649828   -1.080870    1.174450
    4          7           0       -1.659015    0.322182   -0.292194
    5          6           0       -0.272028    0.128762    0.121244
    6          6           0        0.484941    1.421333   -0.217469
    7          8           0        0.141148    2.085950   -1.198385
    8          6           0        0.309375   -1.121970   -0.592204
    9          6           0        1.756927   -1.517715   -0.211600
   10          6           0        1.907755   -1.823443    1.294862
   11          6           0        2.258854   -2.680495   -1.095465
   12          7           0        1.494645    1.753207    0.591678
   13          6           0        2.337319    2.935496    0.431743
   14          1           0       -4.013618    0.840897   -1.112457
   15          1           0       -4.597339   -0.770510   -0.692528
   16          1           0       -4.713696    0.544902    0.480288
   17          1           0       -1.768419    1.024445   -1.015088
   18          1           0       -0.248242   -0.009387    1.203952
   19          1           0       -0.341376   -1.976721   -0.401276
   20          1           0        0.263485   -0.951339   -1.669692
   21          1           0        2.406130   -0.670615   -0.438621
   22          1           0        2.477883   -2.732334    1.488574
   23          1           0        2.432586   -1.013988    1.803315
   24          1           0        0.943664   -1.946640    1.789390
   25          1           0        3.200347   -2.420028   -1.580873
   26          1           0        2.430820   -3.598996   -0.533906
   27          1           0        1.552834   -2.929849   -1.888541
   28          1           0        1.706797    1.144398    1.372076
   29          1           0        2.272903    3.564347    1.320011
   30          1           0        3.379031    2.638144    0.309085
   31          1           0        2.064767    3.548835   -0.428680
 ---------------------------------------------------------------------
```

Ac-Leu-NHMe β-2: M06-2X/6-31+G(d), scrf=(smd, solvent=chloroform)

```
NIMG=1 (34.6573 cm-1)
Zero-point correction=                           0.274078 (Hartree/Particle)
 Thermal correction to Energy=                     0.290127
 Thermal correction to Enthalpy=                   0.291071
 Thermal correction to Gibbs Free Energy=          0.229764
 Sum of electronic and zero-point Energies=          -613.279935
```

Sum of electronic and thermal Energies= -613.263886
Sum of electronic and thermal Enthalpies= -613.262942
Sum of electronic and thermal Free Energies= -613.324249
Standard orientation:

Center Number	Atomic Number	Atomic Type	Coordinates (Angstroms) X	Y	Z
1	6	0	-1.617530	3.269304	-0.443160
2	6	0	-1.173666	1.983130	0.237179
3	8	0	-1.639585	1.687054	1.334488
4	7	0	-0.279803	1.225879	-0.408138
5	6	0	0.339158	0.011907	0.115009
6	6	0	1.835154	0.104999	-0.205372
7	8	0	2.205168	0.761080	-1.180366
8	6	0	-0.273907	-1.237849	-0.565299
9	6	0	-1.743269	-1.539792	-0.188907
10	6	0	-2.320046	-2.671136	-1.068299
11	6	0	-1.918894	-1.824916	1.319007
12	7	0	2.657689	-0.572000	0.600912
13	6	0	4.107974	-0.606437	0.440659
14	1	0	-2.694882	3.246412	-0.604957
15	1	0	-1.386469	4.121857	0.194895
16	1	0	-1.126535	3.414434	-1.405197
17	1	0	0.166593	1.554812	-1.254458
18	1	0	0.222267	-0.038038	1.199184
19	1	0	-0.189952	-1.117861	-1.647205
20	1	0	0.330454	-2.114229	-0.323844
21	1	0	-2.329423	-0.648228	-0.417165
22	1	0	-2.556997	-3.570908	-0.500364
23	1	0	-3.239520	-2.348712	-1.558387
24	1	0	-1.629587	-2.973527	-1.856300
25	1	0	-2.564859	-2.679702	1.518809
26	1	0	-0.968316	-2.020132	1.815233
27	1	0	-2.370954	-0.966339	1.818175
28	1	0	2.263021	-1.088464	1.372895
29	1	0	4.592704	-0.269990	1.357350
30	1	0	4.437620	-1.625684	0.237935
31	1	0	4.465124	0.026811	-0.373906

Ac-Leu-NHMe β-1: M06-2X/6-31+G(d), scrf=(smd, solvent=chloroform)

NIMG=1 (45.9909 cm^{-1})
Zero-point correction= 0.274482 (Hartree/Particle)
Thermal correction to Energy= 0.290325
Thermal correction to Enthalpy= 0.291269
Thermal correction to Gibbs Free Energy= 0.230852
Sum of electronic and zero-point Energies= -613.279326
Sum of electronic and thermal Energies= -613.263484
Sum of electronic and thermal Enthalpies= -613.262540
Sum of electronic and thermal Free Energies= -613.322957
Standard orientation:

Center Number	Atomic Number	Atomic Type	Coordinates (Angstroms) X	Y	Z
1	6	0	-4.085184	0.148158	-0.286780
2	6	0	-2.718738	-0.260968	0.242099
3	8	0	-2.636105	-1.133946	1.102477
4	7	0	-1.650712	0.362961	-0.264932
5	6	0	-0.269218	0.142942	0.143392
6	6	0	0.487918	1.436362	-0.184499
7	8	0	0.091163	2.147907	-1.109015
8	6	0	0.296835	-1.100196	-0.593631

9	6	0	1.736188	-1.528595	-0.215939
10	6	0	1.875599	-1.868405	1.285155
11	6	0	2.221630	-2.681632	-1.122204
12	7	0	1.552002	1.715777	0.573130
13	6	0	2.386129	2.900880	0.401783
14	1	0	-4.582895	-0.715285	-0.727306
15	1	0	-4.702177	0.516177	0.532473
16	1	0	-4.013853	0.929124	-1.043640
17	1	0	-1.736502	1.118663	-0.934684
18	1	0	-0.249500	-0.010458	1.223784
19	1	0	-0.368979	-1.947543	-0.420936
20	1	0	0.255931	-0.905595	-1.667110
21	1	0	2.400589	-0.689266	-0.425445
22	1	0	2.423384	-2.794398	1.460742
23	1	0	2.416033	-1.082396	1.812921
24	1	0	0.905959	-1.981869	1.771845
25	1	0	3.170366	-2.428782	-1.597060
26	1	0	2.372159	-3.614931	-0.579454
27	1	0	1.514780	-2.900116	-1.923560
28	1	0	1.806236	1.066672	1.302635
29	1	0	3.395212	2.608275	0.110927
30	1	0	2.006397	3.586611	-0.358190
31	1	0	2.450417	3.450996	1.340725

Ac-Leu-NHMe β-3: M06-2X/6-31+G(d), scrf=(smd, solvent=chloroform)

```
NIMG=1 (46.7869 cm-1)
Zero-point correction=                           0.274592 (Hartree/Particle)
 Thermal correction to Energy=                    0.290306
 Thermal correction to Enthalpy=                  0.291250
 Thermal correction to Gibbs Free Energy=         0.231237
 Sum of electronic and zero-point Energies=          -613.278672
 Sum of electronic and thermal Energies=             -613.262958
 Sum of electronic and thermal Enthalpies=           -613.262014
 Sum of electronic and thermal Free Energies=        -613.322027
Standard orientation:
```

Center Number	Atomic Number	Atomic Type	Coordinates (Angstroms) X	Y	Z
1	6	0	-3.548951	-1.101921	0.711459
2	6	0	-2.443948	-0.774140	-0.281359
3	8	0	-2.726104	-0.565573	-1.458775
4	7	0	-1.192135	-0.728768	0.185238
5	6	0	0.002146	-0.474680	-0.610567
6	6	0	1.195719	-1.077081	0.142157
7	8	0	1.072593	-1.387195	1.328636
8	6	0	0.157070	1.039523	-0.937790
9	6	0	0.627787	1.987183	0.199703
10	6	0	0.972005	3.385224	-0.360334
11	6	0	-0.377247	2.080641	1.366123
12	7	0	2.324282	-1.220528	-0.558337
13	6	0	3.560443	-1.765538	-0.006455
14	1	0	-4.268176	-0.284019	0.744020
15	1	0	-4.072014	-2.004273	0.396220
16	1	0	-3.160530	-1.260524	1.717267
17	1	0	-0.965480	-0.963134	1.144486
18	1	0	-0.105820	-1.025679	-1.547375
19	1	0	0.889115	1.105348	-1.743794

20	1	0	-0.769871	1.416125	-1.373405
21	1	0	1.559805	1.595585	0.610015
22	1	0	0.305282	4.163268	0.012299
23	1	0	1.987835	3.673619	-0.087551
24	1	0	0.912031	3.421870	-1.448648
25	1	0	-0.124143	1.363846	2.148419
26	1	0	-0.386760	3.065584	1.833359
27	1	0	-1.396374	1.869560	1.042125
28	1	0	2.333996	-0.933980	-1.525704
29	1	0	4.365960	-1.037684	-0.105790
30	1	0	3.480088	-2.028434	1.050181
31	1	0	3.847968	-2.665126	-0.550950

Ac-Val-NHMe PPII-2: M06-2X/6-31+G(d), scrf=(smd, solvent=water)

```
NIMG=1 (46.0819 cm-1)
Zero-point correction=                           0.245760 (Hartree/Particle)
 Thermal correction to Energy=                     0.260319
 Thermal correction to Enthalpy=                   0.261263
 Thermal correction to Gibbs Free Energy=          0.204124
 Sum of electronic and zero-point Energies=           -574.019435
 Sum of electronic and thermal Energies=              -574.004877
 Sum of electronic and thermal Enthalpies=            -574.003933
 Sum of electronic and thermal Free Energies=         -574.061071
Standard orientation:
```

Center Number	Atomic Number	Atomic Type	Coordinates (Angstroms) X	Y	Z
1	6	0	-3.366796	-1.109261	0.566164
2	6	0	-2.044446	-0.971725	-0.174285
3	8	0	-1.723589	-1.815034	-1.014022
4	7	0	-1.283583	0.088770	0.123730
5	6	0	0.044531	0.347527	-0.434622
6	6	0	1.120072	-0.572212	0.187828
7	8	0	0.953371	-1.054380	1.311055
8	6	0	0.457251	1.834247	-0.219345
9	6	0	-0.449114	2.787994	-1.019003
10	6	0	0.547857	2.281081	1.256551
11	7	0	2.212170	-0.783273	-0.553925
12	6	0	3.348992	-1.607217	-0.149674
13	1	0	-3.530188	-0.288469	1.264356
14	1	0	-4.194663	-1.120983	-0.142318
15	1	0	-3.385882	-2.041907	1.129402
16	1	0	-1.583466	0.730425	0.842709
17	1	0	0.007803	0.145297	-1.507879
18	1	0	1.459184	1.954462	-0.637653
19	1	0	-0.108999	3.820684	-0.934490
20	1	0	-0.450460	2.534256	-2.079783
21	1	0	-1.481869	2.753532	-0.670611
22	1	0	0.841464	3.328836	1.329311
23	1	0	-0.406453	2.176042	1.772729
24	1	0	1.290070	1.708711	1.813577
25	1	0	2.268564	-0.346763	-1.466068
26	1	0	3.243080	-2.032475	0.849647
27	1	0	3.479907	-2.433569	-0.848849
28	1	0	4.263468	-1.013851	-0.160663

Ac-Val-NHMe PPII-3: M06-2X/6-31+G(d), scrf=(smd, solvent=water)

```
NIMG=1 (42.9671 cm-1)
Zero-point correction=                           0.245369 (Hartree/Particle)
 Thermal correction to Energy=                     0.259990
```

Thermal correction to Enthalpy= 0.260934
Thermal correction to Gibbs Free Energy= 0.203405
Sum of electronic and zero-point Energies= -574.019374
Sum of electronic and thermal Energies= -574.004752
Sum of electronic and thermal Enthalpies= -574.003808
Sum of electronic and thermal Free Energies= -574.061338
Standard orientation:

Center Number	Atomic Number	Atomic Type	Coordinates (Angstroms) X	Y	Z
1	6	0	-3.720589	-0.009427	0.344382
2	6	0	-2.365732	-0.291708	-0.288427
3	8	0	-2.227044	-1.270830	-1.023950
4	7	0	-1.371773	0.563494	-0.015766
5	6	0	0.010747	0.411551	-0.471446
6	6	0	0.734684	-0.709903	0.312443
7	8	0	0.457760	-0.914021	1.497212
8	6	0	0.774721	1.768162	-0.347138
9	6	0	0.917910	2.297794	1.096225
10	6	0	2.151481	1.759359	-1.039632
11	7	0	1.644961	-1.416284	-0.365773
12	6	0	2.438167	-2.509294	0.190846
13	1	0	-3.704290	0.891035	0.958314
14	1	0	-4.476622	0.121821	-0.429332
15	1	0	-4.020408	-0.844812	0.976560
16	1	0	-1.538208	1.328123	0.622025
17	1	0	-0.018814	0.130519	-1.526963
18	1	0	0.176709	2.503799	-0.888525
19	1	0	1.373797	3.288448	1.104547
20	1	0	-0.048468	2.388346	1.592241
21	1	0	1.545154	1.650789	1.710221
22	1	0	2.590068	2.757915	-1.050702
23	1	0	2.861359	1.102384	-0.536526
24	1	0	2.070451	1.432777	-2.077007
25	1	0	1.807575	-1.184719	-1.337881
26	1	0	3.500706	-2.305477	0.056357
27	1	0	2.265457	-2.671511	1.255941
28	1	0	2.209161	-3.440314	-0.328106

Ac-Val-NHMe β-2: M06-2X/6-31+G(d), scrf=(smd, solvent=water)

NIMG=1 (56.6010 cm^{-1})
Zero-point correction= 0.245409 (Hartree/Particle)
Thermal correction to Energy= 0.259821
Thermal correction to Enthalpy= 0.260766
Thermal correction to Gibbs Free Energy= 0.204140
Sum of electronic and zero-point Energies= -574.019364
Sum of electronic and thermal Energies= -574.004951
Sum of electronic and thermal Enthalpies= -574.004007
Sum of electronic and thermal Free Energies= -574.060633
Standard orientation:

Center Number	Atomic Number	Atomic Type	Coordinates (Angstroms) X	Y	Z
1	6	0	-3.224610	-1.489311	0.701361
2	6	0	-2.193787	-0.849177	-0.216813
3	8	0	-2.441612	-0.722948	-1.417333
4	7	0	-1.046989	-0.448001	0.341467
5	6	0	0.092946	0.134946	-0.364020
6	6	0	1.382305	-0.562119	0.112919
7	8	0	1.393796	-1.168353	1.187909
8	6	0	0.209782	1.667608	-0.095253
9	6	0	-0.955970	2.446919	-0.729954
10	6	0	0.376107	2.059735	1.389719
11	7	0	2.442880	-0.461489	-0.694297

12	6	0	3.755488	-1.042331	-0.421918
13	1	0	-2.882694	-1.524418	1.735785
14	1	0	-4.155633	-0.923843	0.671978
15	1	0	-3.433565	-2.508791	0.378390
16	1	0	-0.862671	-0.646019	1.316427
17	1	0	0.001818	-0.042089	-1.438226
18	1	0	1.110359	2.014867	-0.606444
19	1	0	-0.814664	3.523513	-0.628961
20	1	0	-1.042432	2.228845	-1.795018
21	1	0	-1.909203	2.196337	-0.263676
22	1	0	0.447473	3.142157	1.502327
23	1	0	-0.470230	1.726445	1.991004
24	1	0	1.281217	1.638648	1.828271
25	1	0	2.344292	0.054251	-1.560322
26	1	0	4.515418	-0.260645	-0.412253
27	1	0	3.808225	-1.566082	0.533945
28	1	0	4.020017	-1.753965	-1.204263

Ac-Val-NHMe β-3: M06-2X/6-31+G(d), scrf=(smd, solvent=water)

```
NIMG=1 (46.1062 cm-1)
Zero-point correction=                       0.245266 (Hartree/Particle)
 Thermal correction to Energy=                 0.259805
 Thermal correction to Enthalpy=               0.260749
 Thermal correction to Gibbs Free Energy=      0.203430
 Sum of electronic and zero-point Energies=        -574.018577
 Sum of electronic and thermal Energies=           -574.004038
 Sum of electronic and thermal Enthalpies=         -574.003094
 Sum of electronic and thermal Free Energies=      -574.060413
Standard orientation:
```

Center Number	Atomic Number	Atomic Type	Coordinates (Angstroms) X	Y	Z
1	6	0	-3.583458	-1.166700	0.369449
2	6	0	-2.465661	-0.419723	-0.343028
3	8	0	-2.716346	0.237987	-1.354530
4	7	0	-1.237670	-0.517812	0.175081
5	6	0	-0.030284	0.098985	-0.363470
6	6	0	1.173994	-0.730933	0.125303
7	8	0	1.084982	-1.373104	1.175782
8	6	0	0.086167	1.596961	0.071698
9	6	0	0.190417	1.812870	1.596774
10	6	0	1.219404	2.356406	-0.644419
11	7	0	2.262475	-0.724873	-0.649863
12	6	0	3.498141	-1.447182	-0.358407
13	1	0	-3.220594	-1.700950	1.247460
14	1	0	-4.354746	-0.468211	0.693043
15	1	0	-4.039839	-1.891706	-0.304089
16	1	0	-1.058920	-1.101375	0.984349
17	1	0	-0.065826	0.036139	-1.453730
18	1	0	-0.838612	2.081778	-0.246207
19	1	0	0.176242	2.875128	1.842958
20	1	0	-0.645959	1.352094	2.123154
21	1	0	1.110710	1.398125	2.009091
22	1	0	1.173096	3.423764	-0.425131
23	1	0	2.206328	2.009092	-0.338066
24	1	0	1.145816	2.247358	-1.727104
25	1	0	2.245560	-0.175175	-1.500073
26	1	0	4.338211	-0.752984	-0.328874
27	1	0	3.475262	-1.978311	0.594551
28	1	0	3.698235	-2.179146	-1.141258

Ac-Val-NHMe β-2: M06-2X/6-31+G(d), scrf=(smd, solvent=chloroform)

```
NIMG=1 (50.1132 cm-1)
Zero-point correction=                           0.245395 (Hartree/Particle)
 Thermal correction to Energy=                     0.259973
 Thermal correction to Enthalpy=                   0.260917
 Thermal correction to Gibbs Free Energy=          0.203827
 Sum of electronic and zero-point Energies=           -574.014674
 Sum of electronic and thermal Energies=              -574.000096
 Sum of electronic and thermal Enthalpies=            -573.999152
 Sum of electronic and thermal Free Energies=         -574.056242
Standard orientation:
```

Center Number	Atomic Number	Atomic Type	Coordinates (Angstroms) X	Y	Z
1	6	0	-3.154261	-1.631880	0.637057
2	6	0	-2.179917	-0.843009	-0.224925
3	8	0	-2.504525	-0.518217	-1.364570
4	7	0	-0.994563	-0.537320	0.312226
5	6	0	0.102760	0.131383	-0.376422
6	6	0	1.410463	-0.535040	0.084464
7	8	0	1.435843	-1.142916	1.156490
8	6	0	0.160429	1.655456	-0.046152
9	6	0	-1.022073	2.421156	-0.666355
10	6	0	0.294206	1.991041	1.456223
11	7	0	2.464199	-0.402932	-0.727120
12	6	0	3.781647	-0.965515	-0.448255
13	1	0	-3.399305	-2.574080	0.147556
14	1	0	-2.743985	-1.850755	1.622619
15	1	0	-4.075261	-1.063770	0.764475
16	1	0	-0.714207	-0.899378	1.214591
17	1	0	0.007125	-0.016588	-1.454578
18	1	0	1.055486	2.052757	-0.528721
19	1	0	-0.913360	3.497671	-0.532536
20	1	0	-1.098198	2.229267	-1.737314
21	1	0	-1.970503	2.128249	-0.215231
22	1	0	0.329602	3.069278	1.614685
23	1	0	-0.550168	1.605793	2.028239
24	1	0	1.204518	1.577526	1.891337
25	1	0	2.351290	0.109650	-1.588932
26	1	0	3.823474	-1.515375	0.494099
27	1	0	4.070932	-1.651361	-1.244780
28	1	0	4.525030	-0.169597	-0.400425

Ac-Val-NHMe β-4: M06-2X/6-31+G(d), scrf=(smd, solvent=chloroform)

```
NIMG=1 (27.8585 cm-1)
Zero-point correction=                           0.245466 (Hartree/Particle)
 Thermal correction to Energy=                     0.260183
 Thermal correction to Enthalpy=                   0.261127
 Thermal correction to Gibbs Free Energy=          0.202720
 Sum of electronic and zero-point Energies=           -574.014374
 Sum of electronic and thermal Energies=              -573.999657
 Sum of electronic and thermal Enthalpies=            -573.998713
 Sum of electronic and thermal Free Energies=         -574.057120
Standard orientation:
```

Center Number	Atomic Number	Atomic Type	Coordinates (Angstroms) X	Y	Z

1	6	0	3.327253	-1.537771	-0.429686
2	6	0	2.269876	-0.705122	0.279570
3	8	0	2.493433	-0.275218	1.408506
4	7	0	1.127045	-0.476889	-0.376443
5	6	0	-0.036798	0.211542	0.168420
6	6	0	-1.249055	-0.646511	-0.231225
7	8	0	-1.254787	-1.197796	-1.333198
8	6	0	-0.197264	1.639510	-0.442824
9	6	0	-1.476381	2.383520	0.000690
10	6	0	1.028386	2.526345	-0.145658
11	7	0	-2.238104	-0.745978	0.662284
12	6	0	-3.456796	-1.518449	0.445024
13	1	0	3.001710	-1.856234	-1.419743
14	1	0	4.242117	-0.955781	-0.537515
15	1	0	3.554806	-2.424540	0.161162
16	1	0	0.932227	-0.922816	-1.263457
17	1	0	0.024156	0.269036	1.257472
18	1	0	-0.258417	1.535440	-1.528345
19	1	0	-1.500858	3.397528	-0.400011
20	1	0	-2.383176	1.891517	-0.352127
21	1	0	-1.535128	2.459035	1.086872
22	1	0	0.900662	3.533751	-0.542598
23	1	0	1.204975	2.610091	0.927377
24	1	0	1.935417	2.122255	-0.595131
25	1	0	-2.141961	-0.264635	1.543958
26	1	0	-3.511454	-2.339421	1.160301
27	1	0	-4.331183	-0.883942	0.590213
28	1	0	-3.520963	-1.945490	-0.557655

Ac-Val-NHMe β-3: M06-2X/6-31+G(d), scrf=(smd, solvent=chloroform)

NIMG=1 (44.8290 cm^{-1})
Zero-point correction= 0.245246 (Hartree/Particle)
Thermal correction to Energy= 0.259889
Thermal correction to Enthalpy= 0.260833
Thermal correction to Gibbs Free Energy= 0.203318
Sum of electronic and zero-point Energies= -574.014475
Sum of electronic and thermal Energies= -573.999832
Sum of electronic and thermal Enthalpies= -573.998887
Sum of electronic and thermal Free Energies= -574.056402
Standard orientation:

Center Number	Atomic Number	Atomic Type	Coordinates (Angstroms) X	Y	Z
1	6	0	-3.570356	-1.187182	0.349144
2	6	0	-2.459645	-0.405996	-0.336648
3	8	0	-2.721857	0.298869	-1.308052
4	7	0	-1.225300	-0.528287	0.160014
5	6	0	-0.030389	0.106859	-0.376597
6	6	0	1.166884	-0.736812	0.099164
7	8	0	1.055930	-1.417243	1.121349
8	6	0	0.085931	1.592882	0.094776
9	6	0	0.216990	1.769258	1.623344
10	6	0	1.200412	2.377989	-0.623749
11	7	0	2.270034	-0.696165	-0.654159
12	6	0	3.488981	-1.441101	-0.356157
13	1	0	-3.200058	-1.769309	1.192805
14	1	0	-4.333485	-0.499483	0.712530

15	1	0	-4.035700	-1.868102	-0.362917
16	1	0	-1.009945	-1.148127	0.932208
17	1	0	-0.075724	0.063982	-1.467163
18	1	0	-0.849083	2.077055	-0.193752
19	1	0	0.201838	2.824216	1.898511
20	1	0	-0.607574	1.288592	2.150390
21	1	0	1.145665	1.346596	2.008170
22	1	0	1.151512	3.438450	-0.373837
23	1	0	2.194702	2.028502	-0.345232
24	1	0	1.103507	2.298416	-1.707017
25	1	0	2.270625	-0.109509	-1.474937
26	1	0	3.757677	-2.071735	-1.203681
27	1	0	4.312703	-0.750862	-0.172999
28	1	0	3.394762	-2.085903	0.519891

Ac-Ile-NHMe PPII-2: M06-2X/6-31+G(d), scrf=(smd, solvent=water)

```
NIMG=1 (45.3129 cm-1)
Zero-point correction=                           0.274232 (Hartree/Particle)
 Thermal correction to Energy=                    0.290166
 Thermal correction to Enthalpy=                  0.291110
 Thermal correction to Gibbs Free Energy=         0.230407
 Sum of electronic and zero-point Energies=          -613.283027
 Sum of electronic and thermal Energies=             -613.267093
 Sum of electronic and thermal Enthalpies=           -613.266148
 Sum of electronic and thermal Free Energies=        -613.326852
Standard orientation:
```

Center Number	Atomic Number	Atomic Type	Coordinates (Angstroms) X	Y	Z
1	6	0	-2.253682	-2.895267	-0.353838
2	6	0	-1.605902	-1.654412	0.195462
3	8	0	-2.140375	-0.984744	1.096021
4	7	0	-0.416788	-1.302898	-0.325085
5	6	0	0.261804	-0.081200	0.066835
6	6	0	-0.617112	1.128055	-0.262739
7	8	0	-1.186873	1.225704	-1.362430
8	6	0	1.606241	0.036267	-0.677835
9	6	0	2.547009	-1.139875	-0.363713
10	6	0	2.273072	1.381288	-0.381454
11	6	0	2.938683	-1.275338	1.106435
12	7	0	-0.696159	2.080511	0.670105
13	6	0	-1.366229	3.342924	0.408756
14	1	0	-3.239349	-2.628172	-0.745280
15	1	0	-1.664093	-3.364527	-1.143789
16	1	0	-2.396848	-3.607287	0.464009
17	1	0	-0.058403	-1.827177	-1.115622
18	1	0	0.439087	-0.102162	1.149180
19	1	0	1.375824	-0.000756	-1.752092
20	1	0	2.101342	-2.080890	-0.707415
21	1	0	3.451340	-0.995622	-0.967772
22	1	0	3.286986	1.391878	-0.794439
23	1	0	1.721855	2.213474	-0.831912
24	1	0	2.343644	1.568538	0.696208
25	1	0	2.073587	-1.501312	1.739596
26	1	0	3.657479	-2.091432	1.232117
27	1	0	3.406592	-0.360971	1.486764
28	1	0	-0.183203	1.960720	1.535572
29	1	0	-1.297604	3.961625	1.302472
30	1	0	-0.893335	3.864174	-0.429317
31	1	0	-2.419587	3.174264	0.170400

Ac-Ile-NHMe PPII-1: M06-2X/6-31+G(d), scrf=(smd, solvent=water)

```
NIMG=1 (46.5549 cm-1)
Zero-point correction=                           0.274638 (Hartree/Particle)
 Thermal correction to Energy=                     0.290424
 Thermal correction to Enthalpy=                   0.291368
 Thermal correction to Gibbs Free Energy=          0.231372
 Sum of electronic and zero-point Energies=          -613.282467
 Sum of electronic and thermal Energies=             -613.266682
 Sum of electronic and thermal Enthalpies=           -613.265737
 Sum of electronic and thermal Free Energies=        -613.325733
Standard orientation:
```

Center Number	Atomic Number	Atomic Type	Coordinates (Angstroms) X	Y	Z
1	6	0	3.752967	-1.299325	0.201342
2	6	0	2.564559	-0.555239	-0.390079
3	8	0	2.752322	0.327542	-1.229372
4	7	0	1.345400	-0.912284	0.033735
5	6	0	0.096674	-0.260223	-0.364580
6	6	0	-0.048905	1.121715	0.317065
7	8	0	0.388279	1.297429	1.457170
8	6	0	-1.126649	-1.174699	-0.034473
9	6	0	-2.452667	-0.651638	-0.636662
10	6	0	-1.298049	-1.490628	1.469386
11	6	0	-3.579313	-1.695724	-0.658093
12	7	0	-0.658363	2.074928	-0.395550
13	6	0	-0.899786	3.438766	0.068716
14	1	0	4.413978	-0.601751	0.714954
15	1	0	3.442455	-2.060753	0.916703
16	1	0	4.320991	-1.789051	-0.589156
17	1	0	1.257899	-1.619473	0.749021
18	1	0	0.133728	-0.111035	-1.446424
19	1	0	-0.920341	-2.125697	-0.529090
20	1	0	-2.797087	0.223479	-0.083401
21	1	0	-2.282726	-0.311277	-1.659272
22	1	0	-2.066194	-2.247082	1.630647
23	1	0	-0.380269	-1.878836	1.910476
24	1	0	-1.589546	-0.608491	2.040161
25	1	0	-4.334021	-1.436479	-1.401351
26	1	0	-3.206878	-2.690804	-0.903845
27	1	0	-4.081323	-1.755513	0.308077
28	1	0	-0.983181	1.847893	-1.327044
29	1	0	-0.435332	4.153128	-0.611404
30	1	0	-1.970435	3.643404	0.086600
31	1	0	-0.508782	3.632042	1.068952

Ac-Ile-NHMe PPII-3: M06-2X/6-31+G(d), scrf=(smd, solvent=water)

```
NIMG=1 (42.1927 cm-1)
Zero-point correction=                           0.273875 (Hartree/Particle)
 Thermal correction to Energy=                     0.289920
 Thermal correction to Enthalpy=                   0.290864
 Thermal correction to Gibbs Free Energy=          0.229940
 Sum of electronic and zero-point Energies=          -613.283428
 Sum of electronic and thermal Energies=             -613.267383
 Sum of electronic and thermal Enthalpies=           -613.266438
 Sum of electronic and thermal Free Energies=        -613.327363
```

Standard orientation:

Center Number	Atomic Number	Atomic Type	Coordinates (Angstroms) X	Y	Z
1	6	0	-3.408441	-1.414230	-0.741578
2	6	0	-2.445697	-0.529869	0.037409
3	8	0	-2.886239	0.378900	0.744059
4	7	0	-1.139016	-0.796996	-0.079035
5	6	0	-0.067570	-0.014744	0.540223
6	6	0	0.159717	1.334151	-0.180254
7	8	0	-0.163054	1.470177	-1.363134
8	6	0	1.268494	-0.817601	0.565429
9	6	0	1.782718	-1.265705	-0.828008
10	6	0	1.162724	-2.013873	1.532337
11	6	0	3.199446	-1.859069	-0.800698
12	7	0	0.724797	2.301734	0.548936
13	6	0	1.034933	3.642622	0.058916
14	1	0	-3.986533	-0.812476	-1.442392
15	1	0	-2.886439	-2.185492	-1.307739
16	1	0	-4.102241	-1.905608	-0.059974
17	1	0	-0.835398	-1.535550	-0.696195
18	1	0	-0.362948	0.205175	1.569020
19	1	0	2.027927	-0.153055	0.983978
20	1	0	1.102116	-2.003344	-1.254813
21	1	0	1.785670	-0.429553	-1.527590
22	1	0	2.125245	-2.506043	1.671813
23	1	0	0.824337	-1.694857	2.518751
24	1	0	0.458977	-2.763943	1.170453
25	1	0	3.670219	-1.785246	-1.781563
26	1	0	3.842804	-1.339938	-0.089520
27	1	0	3.180890	-2.914433	-0.526999
28	1	0	0.959631	2.104928	1.514253
29	1	0	0.503879	4.389167	0.649850
30	1	0	2.102870	3.838874	0.156582
31	1	0	0.764912	3.795527	-0.987286

Ac-Ile-NHMe β-1: M06-2X/6-31+G(d), scrf=(smd, solvent=water)

NIMG=1 (35.8919 cm^{-1})
Zero-point correction= 0.274271 (Hartree/Particle)
Thermal correction to Energy= 0.289979
Thermal correction to Enthalpy= 0.290923
Thermal correction to Gibbs Free Energy= 0.231187
Sum of electronic and zero-point Energies= -613.282268
Sum of electronic and thermal Energies= -613.266560
Sum of electronic and thermal Enthalpies= -613.265616
Sum of electronic and thermal Free Energies= -613.325351
Standard orientation:

Center Number	Atomic Number	Atomic Type	Coordinates (Angstroms) X	Y	Z
1	6	0	-3.820284	-1.011575	-0.064407
2	6	0	-2.585993	-0.205774	-0.441634
3	8	0	-2.699070	0.783622	-1.167220
4	7	0	-1.412393	-0.622388	0.043056
5	6	0	-0.114127	-0.007604	-0.213535

6	6	0	0.959795	-1.091707	0.009057
7	8	0	0.723086	-2.037035	0.767018
8	6	0	0.122898	1.232413	0.714944
9	6	0	1.378899	2.065657	0.366212
10	6	0	0.130177	0.893478	2.221797
11	6	0	1.412562	2.576086	-1.082493
12	7	0	2.103685	-0.953723	-0.667232
13	6	0	3.234902	-1.874469	-0.591205
14	1	0	-4.532991	-0.380237	0.465542
15	1	0	-4.305712	-1.397021	-0.960588
16	1	0	-3.573701	-1.856895	0.578033
17	1	0	-1.348656	-1.464748	0.603527
18	1	0	-0.082609	0.293026	-1.262781
19	1	0	-0.728675	1.897843	0.562433
20	1	0	1.426972	2.931044	1.029160
21	1	0	2.284891	1.495743	0.575856
22	1	0	0.169160	1.800146	2.826465
23	1	0	-0.770429	0.354242	2.516189
24	1	0	0.988715	0.282091	2.501186
25	1	0	0.413551	2.815623	-1.449032
26	1	0	2.013718	3.482759	-1.159757
27	1	0	1.848753	1.839487	-1.757046
28	1	0	2.210878	-0.148734	-1.271090
29	1	0	3.431494	-2.308568	-1.571809
30	1	0	4.131545	-1.338985	-0.278063
31	1	0	3.078954	-2.695769	0.110211

Ac-Ile-NHMe β-3: M06-2X/6-31+G(d), scrf=(smd, solvent=water)

```
NIMG=1 (53.9039 cm-1)
Zero-point correction=                           0.274173 (Hartree/Particle)
 Thermal correction to Energy=                    0.290101
 Thermal correction to Enthalpy=                  0.291045
 Thermal correction to Gibbs Free Energy=         0.230316
 Sum of electronic and zero-point Energies=          -613.282642
 Sum of electronic and thermal Energies=             -613.266715
 Sum of electronic and thermal Enthalpies=           -613.265770
 Sum of electronic and thermal Free Energies=        -613.326499
Standard orientation:
```

Center Number	Atomic Number	Atomic Type	Coordinates (Angstroms) X	Y	Z
1	6	0	3.456321	-1.142127	-0.991405
2	6	0	2.390347	-0.852089	0.054896
3	8	0	2.676446	-0.919301	1.251640
4	7	0	1.170875	-0.532890	-0.390747
5	6	0	-0.005612	-0.272960	0.437435
6	6	0	-1.214182	-1.018507	-0.161481
7	8	0	-1.188924	-1.383728	-1.340059
8	6	0	-0.328846	1.252645	0.520882
9	6	0	-0.531127	1.947607	-0.851495
10	6	0	0.722693	1.993829	1.369793
11	6	0	-1.060697	3.385486	-0.743757
12	7	0	-2.247044	-1.230211	0.659807
13	6	0	-3.485010	-1.908524	0.283509
14	1	0	3.069645	-1.042409	-2.005553
15	1	0	4.290889	-0.450773	-0.877973
16	1	0	3.834673	-2.156927	-0.870999

17	1	0	0.968400	-0.569969	-1.381504
18	1	0	0.152429	-0.669080	1.443243
19	1	0	-1.271978	1.344160	1.064309
20	1	0	0.413026	1.959903	-1.397928
21	1	0	-1.224427	1.383267	-1.475286
22	1	0	0.427518	3.023996	1.569479
23	1	0	0.861290	1.508747	2.336591
24	1	0	1.693033	2.019560	0.873420
25	1	0	-1.553359	3.688207	-1.668300
26	1	0	-1.786627	3.490631	0.063218
27	1	0	-0.250688	4.092064	-0.559870
28	1	0	-2.181926	-0.898929	1.614631
29	1	0	-3.503117	-2.231731	-0.758550
30	1	0	-3.632558	-2.791233	0.906233
31	1	0	-4.336545	-1.245705	0.438506

Ac-Ile-NHMe β-2: M06-2X/6-31+G(d), scrf=(smd, solvent=chloroform)

```
NIMG=1 (34.7370 cm-1)
Zero-point correction=                           0.274296 (Hartree/Particle)
 Thermal correction to Energy=                    0.290379
 Thermal correction to Enthalpy=                  0.291324
 Thermal correction to Gibbs Free Energy=         0.229629
 Sum of electronic and zero-point Energies=          -613.278697
 Sum of electronic and thermal Energies=             -613.262613
 Sum of electronic and thermal Enthalpies=           -613.261669
 Sum of electronic and thermal Free Energies=        -613.323364
Standard orientation:
```

Center Number	Atomic Number	Atomic Type	Coordinates (Angstroms) X	Y	Z
1	6	0	2.839929	-2.325921	-0.375996
2	6	0	1.946101	-1.286037	0.262225
3	8	0	2.212362	-0.802936	1.365072
4	7	0	0.874853	-0.909354	-0.463922
5	6	0	-0.133745	0.014832	0.010663
6	6	0	-1.509233	-0.632453	-0.214673
7	8	0	-1.752548	-1.214690	-1.274026
8	6	0	-0.064587	1.344995	-0.758721
9	6	0	1.329742	1.975716	-0.653599
10	6	0	-1.151469	2.315599	-0.291512
11	6	0	1.719109	2.423328	0.756801
12	7	0	-2.407120	-0.461699	0.768813
13	6	0	-3.771874	-0.944014	0.704701
14	1	0	2.488843	-2.652807	-1.359205
15	1	0	3.844545	-1.906894	-0.476477
16	1	0	2.895305	-3.190937	0.289969
17	1	0	0.642050	-1.389970	-1.327875
18	1	0	0.042101	0.185799	1.076197
19	1	0	-0.246482	1.110059	-1.817271
20	1	0	2.078693	1.267154	-1.029408
21	1	0	1.356737	2.839456	-1.329608
22	1	0	-1.024084	3.279175	-0.795466
23	1	0	-2.156087	1.947574	-0.528276
24	1	0	-1.103205	2.491581	0.790482
25	1	0	2.760616	2.761205	0.773363
26	1	0	1.096170	3.255321	1.103875
27	1	0	1.631340	1.602479	1.477407
28	1	0	-2.121790	0.027566	1.608679
29	1	0	-3.937049	-1.738135	1.439794
30	1	0	-4.472936	-0.126126	0.895777
31	1	0	-3.951241	-1.340396	-0.293892

Ac-Ile-NHMe β-1: M06-2X/6-31+G(d), scrf=(smd, solvent=chloroform)

```
NIMG=1 (35.6360 cm-1)
  Zero-point correction=                          0.274047 (Hartree/Particle)
 Thermal correction to Energy=                    0.290153
 Thermal correction to Enthalpy=                  0.291097
 Thermal correction to Gibbs Free Energy=         0.229692
 Sum of electronic and zero-point Energies=           -613.279164
 Sum of electronic and thermal Energies=              -613.263058
 Sum of electronic and thermal Enthalpies=            -613.262114
 Sum of electronic and thermal Free Energies=         -613.323519
Standard orientation:
```

Center Number	Atomic Number	Atomic Type	X	Y	Z
1	6	0	-3.882113	-0.711776	0.262500
2	6	0	-2.616609	-0.171519	-0.360503
3	8	0	-2.646046	0.608920	-1.313846
4	7	0	-1.456505	-0.598528	0.185272
5	6	0	-0.163054	-0.172875	-0.308543
6	6	0	0.837382	-1.260514	0.077924
7	8	0	0.662145	-1.940976	1.067447
8	6	0	0.233586	1.218488	0.255400
9	6	0	1.434250	1.817320	-0.487263
10	6	0	0.453185	1.177958	1.767314
11	6	0	1.647690	3.299292	-0.182023
12	7	0	1.903671	-1.381171	-0.724568
13	6	0	2.999077	-2.298309	-0.470213
14	1	0	-4.461479	-1.223184	-0.511686
15	1	0	-3.692135	-1.403496	1.086555
16	1	0	-4.479872	0.128750	0.626987
17	1	0	-1.457731	-1.249798	0.963060
18	1	0	-0.228229	-0.092009	-1.399801
19	1	0	-0.639104	1.851671	0.042173
20	1	0	2.344229	1.259871	-0.226609
21	1	0	1.282279	1.695590	-1.569192
22	1	0	0.526549	2.190057	2.176674
23	1	0	-0.375186	0.675816	2.278669
24	1	0	1.378167	0.643992	2.018890
25	1	0	2.475650	3.703639	-0.773835
26	1	0	0.749150	3.880278	-0.420851
27	1	0	1.886197	3.464519	0.873640
28	1	0	1.952341	-0.792674	-1.547594
29	1	0	2.747233	-2.897473	0.404888
30	1	0	3.146795	-2.959854	-1.328188
31	1	0	3.925831	-1.749753	-0.275069

Ac-Ile-NHMe β-3: M06-2X/6-31+G(d), scrf=(smd, solvent=chloroform)

```
NIMG=1 (52.3380 cm-1)
  Zero-point correction=                          0.274084 (Hartree/Particle)
 Thermal correction to Energy=                    0.290123
 Thermal correction to Enthalpy=                  0.291067
 Thermal correction to Gibbs Free Energy=         0.230262
 Sum of electronic and zero-point Energies=           -613.279277
 Sum of electronic and thermal Energies=              -613.263238
 Sum of electronic and thermal Enthalpies=            -613.262294
 Sum of electronic and thermal Free Energies=         -613.323099
```

```
Standard orientation:
 ---------------------------------------------------------------------
 Center     Atomic      Atomic             Coordinates (Angstroms)
 Number     Number       Type             X           Y           Z
 ---------------------------------------------------------------------
      1          6           0        3.358295   -1.373640   -0.981982
      2          6           0        2.336518   -0.927282    0.052871
      3          8           0        2.667857   -0.837627    1.232568
      4          7           0        1.103500   -0.650132   -0.382822
      5          6           0       -0.032445   -0.287169    0.456106
      6          6           0       -1.282810   -0.958645   -0.137113
      7          8           0       -1.279671   -1.306791   -1.319502
      8          6           0       -0.253472    1.257564    0.513674
      9          6           0       -0.400818    1.939962   -0.872149
     10          6           0        0.837869    1.943047    1.359409
     11          6           0       -0.834757    3.411947   -0.795469
     12          7           0       -2.320320   -1.123264    0.689372
     13          6           0       -3.584572   -1.736091    0.294562
     14          1           0        2.935968   -1.407180   -1.986005
     15          1           0        4.202910   -0.685385   -0.985266
     16          1           0        3.728307   -2.366927   -0.729330
     17          1           0        0.829680   -0.825190   -1.341183
     18          1           0        0.114906   -0.683938    1.463162
     19          1           0       -1.192308    1.419538    1.046921
     20          1           0        0.546063    1.878936   -1.410079
     21          1           0       -1.124373    1.409727   -1.491962
     22          1           0        0.604664    2.990556    1.549384
     23          1           0        0.949252    1.456364    2.328840
     24          1           0        1.808624    1.905772    0.864750
     25          1           0       -1.561464    3.578567    0.000167
     26          1           0        0.018620    4.065620   -0.613038
     27          1           0       -1.295130    3.729509   -1.731614
     28          1           0       -2.231791   -0.816469    1.646555
     29          1           0       -4.413915   -1.073765    0.543625
     30          1           0       -3.642224   -1.949866   -0.774589
     31          1           0       -3.728395   -2.674919    0.829837
 ---------------------------------------------------------------------
```

Ac-Thr-NHMe PPII-2: M06-2X/6-31+G(d), scrf=(smd, solvent=water)

NIMG=1 (54.7696 cm^{-1})

```
 Zero-point correction=                           0.221486 (Hartree/Particle)
 Thermal correction to Energy=                    0.235750
 Thermal correction to Enthalpy=                  0.236694
 Thermal correction to Gibbs Free Energy=         0.180114
 Sum of electronic and zero-point Energies=           -609.950578
 Sum of electronic and thermal Energies=              -609.936314
 Sum of electronic and thermal Enthalpies=            -609.935370
 Sum of electronic and thermal Free Energies=         -609.991951
Standard orientation:
 ---------------------------------------------------------------------
 Center     Atomic      Atomic             Coordinates (Angstroms)
 Number     Number       Type             X           Y           Z
 ---------------------------------------------------------------------
      1          6           0       -3.637843   -0.465644    0.301950
      2          6           0       -2.216836   -0.571405   -0.232235
      3          8           0       -1.910937   -1.508208   -0.972293
      4          7           0       -1.355543    0.388224    0.125675
      5          6           0        0.055810    0.453039   -0.256151
      6          6           0        0.876674   -0.717650    0.335726
```

7	8	0	0.699456	-1.054143	1.509281
8	6	0	0.675114	1.792750	0.235510
9	8	0	-0.160397	2.870863	-0.167251
10	6	0	2.098427	2.078243	-0.276750
11	7	0	1.760912	-1.306323	-0.478049
12	6	0	2.629775	-2.418042	-0.098965
13	1	0	-3.882116	-1.346627	0.894980
14	1	0	-3.772000	0.414107	0.931338
15	1	0	-4.346109	-0.402687	-0.523859
16	1	0	-1.664620	1.156682	0.704188
17	1	0	0.108428	0.408466	-1.346108
18	1	0	0.705132	1.793232	1.328306
19	1	0	0.236126	3.698700	0.146287
20	1	0	2.457659	3.052239	0.057707
21	1	0	2.814259	1.341721	0.089843
22	1	0	2.143157	2.064251	-1.366398
23	1	0	1.841133	-0.974600	-1.431604
24	1	0	2.449752	-3.272626	-0.751626
25	1	0	3.675596	-2.130151	-0.208513
26	1	0	2.484312	-2.751862	0.929680

Ac-Thr-NHMe PPII-1: M06-2X/6-31+G(d), scrf=(smd, solvent=water)

NIMG=1 (46.3054 cm^{-1})

```
  Zero-point correction=                           0.221959 (Hartree/Particle)
 Thermal correction to Energy=                    0.236130
 Thermal correction to Enthalpy=                  0.237074
 Thermal correction to Gibbs Free Energy=          0.180925
 Sum of electronic and zero-point Energies=           -609.950024
 Sum of electronic and thermal Energies=              -609.935853
 Sum of electronic and thermal Enthalpies=            -609.934909
 Sum of electronic and thermal Free Energies=         -609.991058
Standard orientation:
```

Center Number	Atomic Number	Atomic Type	Coordinates (Angstroms) X	Y	Z
1	6	0	-3.688352	-0.127018	0.388644
2	6	0	-2.336642	-0.359752	-0.270497
3	8	0	-2.182417	-1.320798	-1.026407
4	7	0	-1.362244	0.516622	0.002020
5	6	0	0.014540	0.408916	-0.480760
6	6	0	0.785304	-0.685624	0.302512
7	8	0	0.516480	-0.899292	1.488413
8	6	0	0.722221	1.799787	-0.387053
9	8	0	2.028440	1.788901	-0.941670
10	6	0	0.859959	2.369222	1.038488
11	7	0	1.715632	-1.369953	-0.373466
12	6	0	2.534273	-2.442060	0.187939
13	1	0	-3.953522	-0.981022	1.011249
14	1	0	-3.687728	0.763258	1.017335
15	1	0	-4.460403	-0.005026	-0.370634
16	1	0	-1.536830	1.266241	0.655087
17	1	0	-0.026962	0.113808	-1.531959
18	1	0	0.137922	2.509863	-0.976347
19	1	0	1.949795	1.723940	-1.907047
20	1	0	1.313725	3.360818	1.023586
21	1	0	-0.110622	2.474140	1.523288
22	1	0	1.478663	1.738452	1.677489
23	1	0	1.888516	-1.125785	-1.340728

24	1	0	2.481718	-3.324877	-0.449585
25	1	0	3.577541	-2.129776	0.239096
26	1	0	2.226623	-2.742036	1.190970

Ac-Thr-NHMe PPII-3: M06-2X/6-31+G(d), scrf=(smd, solvent=water)

NIMG=1 (47.9349 cm^{-1})

```
 Zero-point correction=                          0.221685 (Hartree/Particle)
 Thermal correction to Energy=                   0.235949
 Thermal correction to Enthalpy=                 0.236893
 Thermal correction to Gibbs Free Energy=        0.180066
 Sum of electronic and zero-point Energies=         -609.951544
 Sum of electronic and thermal Energies=            -609.937279
 Sum of electronic and thermal Enthalpies=          -609.936335
 Sum of electronic and thermal Free Energies=       -609.993162
```

Standard orientation:

Center Number	Atomic Number	Atomic Type	Coordinates (Angstroms) X	Y	Z
1	6	0	-3.087476	-1.606747	0.552049
2	6	0	-1.798365	-1.254121	-0.175399
3	8	0	-1.315908	-2.046903	-0.986256
4	7	0	-1.243451	-0.068944	0.100388
5	6	0	0.022404	0.414272	-0.448217
6	6	0	1.217340	-0.308778	0.216087
7	8	0	1.201985	-0.532127	1.429598
8	6	0	0.147588	1.940502	-0.181113
9	8	0	-0.087275	2.250672	1.188019
10	6	0	-0.858809	2.764080	-1.000030
11	7	0	2.238489	-0.634944	-0.583678
12	6	0	3.457197	-1.310326	-0.143022
13	1	0	-2.951410	-2.516004	1.136939
14	1	0	-3.402975	-0.812498	1.228584
15	1	0	-3.888962	-1.778694	-0.165782
16	1	0	-1.654780	0.543400	0.791644
17	1	0	0.037338	0.228443	-1.524937
18	1	0	1.156494	2.272154	-0.439747
19	1	0	0.544951	1.739930	1.720304
20	1	0	-0.736881	3.832793	-0.820049
21	1	0	-0.716442	2.599946	-2.068696
22	1	0	-1.891441	2.504134	-0.764607
23	1	0	2.175506	-0.415600	-1.570678
24	1	0	3.600506	-2.230418	-0.709971
25	1	0	4.323941	-0.672898	-0.318931
26	1	0	3.449208	-1.573903	0.915935

Ac-Thr-NHMe β-2: M06-2X/6-31+G(d), scrf=(smd, solvent=water)

NIMG=1 (51.4036 cm^{-1})

```
 Zero-point correction=                          0.221947 (Hartree/Particle)
 Thermal correction to Energy=                   0.236051
 Thermal correction to Enthalpy=                 0.236996
 Thermal correction to Gibbs Free Energy=        0.180991
 Sum of electronic and zero-point Energies=         -609.950484
 Sum of electronic and thermal Energies=            -609.936380
 Sum of electronic and thermal Enthalpies=          -609.935435
 Sum of electronic and thermal Free Energies=       -609.991440
```

Standard orientation:

Center Number	Atomic Number	Atomic Type	Coordinates (Angstroms) X	Y	Z
1	6	0	-3.336715	-1.338694	0.459302
2	6	0	-2.359798	-0.561532	-0.364269
3	8	0	-2.730057	0.174074	-1.296743
4	1	0	-2.932034	-1.468111	1.463055
5	1	0	-4.192905	-0.673987	0.576819
6	1	0	-3.719382	-2.203671	-0.084575
7	7	0	-1.112444	-0.662076	0.077897
8	6	0	0.021547	0.124928	-0.357671
9	6	0	1.252927	-0.650124	0.136464
10	8	0	1.101657	-1.288278	1.201590
11	6	0	-0.019389	1.631323	0.121734
12	8	0	0.805200	2.391117	-0.658758
13	6	0	0.305903	1.845684	1.589492
14	1	0	-0.904650	-1.132565	0.947315
15	1	0	-0.017927	0.097708	-1.446050
16	1	0	-1.062078	1.926829	-0.000914
17	1	0	0.247813	2.846547	-1.294048
18	1	0	-0.133336	2.795306	1.894674
19	1	0	-0.247433	1.065746	2.114022
20	1	0	1.389537	1.835919	1.697399
21	7	0	2.312813	-0.580396	-0.669400
22	6	0	3.499997	-1.381182	-0.434260
23	1	0	2.310281	0.200369	-1.310772
24	1	0	3.874457	-1.283781	0.585415
25	1	0	3.227207	-2.421603	-0.610011
26	1	0	4.156642	-1.125123	-1.265272

Ac-Thr-NHMe β-1: M06-2X/6-31+G(d), scrf=(smd, solvent=water)

NIMG=1 (40.7572 cm^{-1})

```
  Zero-point correction=                           0.221819 (Hartree/Particle)
 Thermal correction to Energy=                    0.235878
 Thermal correction to Enthalpy=                  0.236823
 Thermal correction to Gibbs Free Energy=         0.180453
 Sum of electronic and zero-point Energies=          -609.949354
 Sum of electronic and thermal Energies=             -609.935294
 Sum of electronic and thermal Enthalpies=           -609.934350
 Sum of electronic and thermal Free Energies=        -609.990720
```

Standard orientation:

Center Number	Atomic Number	Atomic Type	Coordinates (Angstroms) X	Y	Z
1	6	0	-3.318494	-1.663370	0.289865
2	6	0	-2.342132	-0.612800	-0.217166
3	8	0	-2.746477	0.313499	-0.921228
4	7	0	-1.062295	-0.735658	0.144138
5	6	0	0.054981	0.119204	-0.253371
6	6	0	1.333482	-0.656015	0.126217
7	8	0	1.329776	-1.379072	1.126783
8	6	0	0.013756	1.494866	0.487821
9	8	0	-1.012997	2.292644	-0.078496
10	6	0	1.295662	2.348661	0.444385
11	7	0	2.389669	-0.515076	-0.682077

12	6	0	3.680484	-1.170597	-0.485012
13	1	0	-2.823830	-2.415500	0.904265
14	1	0	-4.097421	-1.193833	0.889865
15	1	0	-3.793110	-2.170349	-0.549888
16	1	0	-0.767820	-1.503787	0.736816
17	1	0	0.031634	0.258648	-1.336736
18	1	0	-0.239186	1.323477	1.536608
19	1	0	-1.762492	1.696652	-0.279947
20	1	0	1.136461	3.324792	0.904281
21	1	0	2.114488	1.880054	0.991450
22	1	0	1.632731	2.516836	-0.579032
23	1	0	2.306605	0.085646	-1.493393
24	1	0	4.478367	-0.427794	-0.471318
25	1	0	3.741977	-1.736756	0.445873
26	1	0	3.881126	-1.859207	-1.306179

Ac-Thr-NHMe β-2: M06-2X/6-31+G(d), scrf=(smd, solvent=chloroform)

NIMG=1 (38.2691 cm^{-1})

```
 Zero-point correction=                           0.222553 (Hartree/Particle)
 Thermal correction to Energy=                     0.236456
 Thermal correction to Enthalpy=                   0.237400
 Thermal correction to Gibbs Free Energy=          0.181284
 Sum of electronic and zero-point Energies=           -609.939699
 Sum of electronic and thermal Energies=              -609.925797
 Sum of electronic and thermal Enthalpies=            -609.924852
 Sum of electronic and thermal Free Energies=         -609.980968
Standard orientation:
```

Center Number	Atomic Number	Atomic Type	Coordinates (Angstroms) X	Y	Z
1	6	0	-3.317349	-1.658087	0.287109
2	6	0	-2.342390	-0.597659	-0.201120
3	8	0	-2.758219	0.371729	-0.831172
4	7	0	-1.050754	-0.762030	0.094581
5	6	0	0.055193	0.111301	-0.285429
6	6	0	1.328963	-0.668665	0.091717
7	8	0	1.291120	-1.463296	1.033359
8	6	0	-0.004724	1.470137	0.482158
9	8	0	-0.978951	2.287501	-0.136605
10	6	0	1.282503	2.314379	0.532628
11	7	0	2.414619	-0.457535	-0.660791
12	6	0	3.694976	-1.125355	-0.446961
13	1	0	-2.815612	-2.450937	0.841612
14	1	0	-4.064079	-1.202370	0.936946
15	1	0	-3.832850	-2.104683	-0.562796
16	1	0	-0.725043	-1.552334	0.640363
17	1	0	0.030301	0.270049	-1.365508
18	1	0	-0.327336	1.287724	1.509343
19	1	0	-1.747563	1.724120	-0.352112
20	1	0	1.093361	3.278238	1.006648
21	1	0	2.067551	1.828262	1.112604
22	1	0	1.669474	2.512285	-0.467001
23	1	0	2.358962	0.200235	-1.424654
24	1	0	3.713620	-1.740448	0.454991
25	1	0	3.926375	-1.770983	-1.294398
26	1	0	4.491156	-0.385972	-0.357838

Ac-Thr-NHMe β-1: M06-2X/6-31+G(d), scrf=(smd, solvent=chloroform)

NIMG=1 (63.1482 cm^{-1})

```
  Zero-point correction=                           0.222194 (Hartree/Particle)
 Thermal correction to Energy=                    0.236185
 Thermal correction to Enthalpy=                  0.237129
 Thermal correction to Gibbs Free Energy=         0.181687
 Sum of electronic and zero-point Energies=          -609.942130
 Sum of electronic and thermal Energies=             -609.928140
 Sum of electronic and thermal Enthalpies=           -609.927196
 Sum of electronic and thermal Free Energies=        -609.982638
```

Standard orientation:

Center Number	Atomic Number	Atomic Type	Coordinates (Angstroms) X	Y	Z
1	6	0	-3.599869	-1.153319	0.358894
2	6	0	-2.495352	-0.294017	-0.237401
3	8	0	-2.783535	0.644442	-0.975876
4	7	0	-1.234717	-0.607374	0.072704
5	6	0	-0.048968	0.062560	-0.441086
6	6	0	1.159352	-0.844560	-0.109620
7	8	0	0.962599	-1.953043	0.395972
8	6	0	0.101762	1.514089	0.126569
9	8	0	1.139674	2.228463	-0.526176
10	6	0	0.375886	1.594803	1.639617
11	7	0	2.373772	-0.374788	-0.403783
12	6	0	3.619054	-1.098536	-0.177077
13	1	0	-3.203631	-1.948811	0.989736
14	1	0	-4.264845	-0.534175	0.960501
15	1	0	-4.186227	-1.606420	-0.440058
16	1	0	-0.997074	-1.422640	0.626388
17	1	0	-0.136694	0.102035	-1.528491
18	1	0	-0.818132	2.066134	-0.072973
19	1	0	0.792655	2.565958	-1.359968
20	1	0	0.405928	2.631835	1.974752
21	1	0	-0.408660	1.093660	2.206903
22	1	0	1.327206	1.137586	1.912546
23	1	0	2.421755	0.594154	-0.704753
24	1	0	3.460004	-2.154716	0.048338
25	1	0	4.250952	-1.039994	-1.063341
26	1	0	4.162276	-0.654487	0.657236

Ac-Thr-NHMe β-3: M06-2X/6-31+G(d), scrf=(smd, solvent=chloroform)

NIMG=1 (56.0314 cm^{-1})

```
  Zero-point correction=                           0.221728 (Hartree/Particle)
 Thermal correction to Energy=                    0.235957
 Thermal correction to Enthalpy=                  0.236901
 Thermal correction to Gibbs Free Energy=         0.180657
 Sum of electronic and zero-point Energies=          -609.939597
 Sum of electronic and thermal Energies=             -609.925368
 Sum of electronic and thermal Enthalpies=           -609.924424
 Sum of electronic and thermal Free Energies=        -609.980668
```

Standard orientation:

Center Number	Atomic Number	Atomic Type	Coordinates (Angstroms) X	Y	Z

1	6	0	-3.085676	-1.753256	0.485293
2	6	0	-2.148850	-0.826198	-0.274343
3	8	0	-2.518171	-0.318034	-1.329703
4	7	0	-0.942749	-0.601171	0.254151
5	6	0	0.127390	0.160587	-0.379767
6	6	0	1.450083	-0.510520	0.045094
7	8	0	1.454796	-1.296962	0.996461
8	6	0	0.115117	1.651273	0.074893
9	8	0	0.178964	1.782637	1.483809
10	6	0	-1.108700	2.444273	-0.408483
11	7	0	2.535008	-0.222759	-0.683438
12	6	0	3.856598	-0.778629	-0.411787
13	1	0	-2.631874	-2.135090	1.399527
14	1	0	-3.999021	-1.220405	0.748587
15	1	0	-3.355586	-2.600210	-0.145020
16	1	0	-0.608026	-1.112286	1.062703
17	1	0	0.037889	0.093423	-1.466530
18	1	0	1.002821	2.142591	-0.328432
19	1	0	-0.580892	1.333449	1.864139
20	1	0	-1.021197	3.499237	-0.146896
21	1	0	-1.209178	2.382631	-1.492690
22	1	0	-2.038059	2.074537	0.024137
23	1	0	2.453146	0.412718	-1.462520
24	1	0	3.944186	-1.190150	0.595883
25	1	0	4.083051	-1.575107	-1.120992
26	1	0	4.616453	-0.004143	-0.516816

Ac-Cys-NHMe PPII-2: M06-2X/6-31+G(d), scrf=(smd, solvent=water)

NIMG=1 (49.1709 cm^{-1})
Zero-point correction= 0.188615 (Hartree/Particle)
Thermal correction to Energy= 0.201967
Thermal correction to Enthalpy= 0.202911
Thermal correction to Gibbs Free Energy= 0.147942
Sum of electronic and zero-point Energies= -893.640590
Sum of electronic and thermal Energies= -893.627238
Sum of electronic and thermal Enthalpies= -893.626294
Sum of electronic and thermal Free Energies= -893.681263
Standard orientation:

Center Number	Atomic Number	Atomic Type	Coordinates (Angstroms) X	Y	Z
1	6	0	2.253521	2.669931	0.369635
2	6	0	1.183514	1.776913	-0.241528
3	8	0	0.519411	2.184881	-1.196073
4	7	0	1.020293	0.560612	0.293359
5	6	0	0.012145	-0.415003	-0.129710
6	6	0	-1.399264	0.023991	0.311875
7	8	0	-1.552548	0.609236	1.387396
8	6	0	0.317668	-1.816494	0.439498
9	16	0	1.925926	-2.421381	-0.164461
10	7	0	-2.405456	-0.299771	-0.506969
11	6	0	-3.812127	0.009511	-0.260544
12	1	0	2.773683	2.179459	1.192197
13	1	0	2.992098	2.941022	-0.384275
14	1	0	1.803832	3.586134	0.751543
15	1	0	1.588321	0.281284	1.080208
16	1	0	0.028980	-0.467800	-1.220703
17	1	0	0.314884	-1.802896	1.530835
18	1	0	-0.457463	-2.523992	0.138056

19	1	0	1.547860	-2.595675	-1.439165
20	1	0	-2.192246	-0.787780	-1.368289
21	1	0	-4.399624	-0.908718	-0.239907
22	1	0	-3.983101	0.532367	0.681954
23	1	0	-4.203555	0.636768	-1.061758

Ac-Cys-NHMe PPII-1: M06-2X/6-31+G(d), scrf=(smd, solvent=water)

NIMG=1 (39.7167 cm^{-1})

```
  Zero-point correction=                           0.188313 (Hartree/Particle)
 Thermal correction to Energy=                     0.201788
 Thermal correction to Enthalpy=                   0.202732
 Thermal correction to Gibbs Free Energy=          0.146969
 Sum of electronic and zero-point Energies=          -893.640814
 Sum of electronic and thermal Energies=             -893.627339
 Sum of electronic and thermal Enthalpies=           -893.626395
 Sum of electronic and thermal Free Energies=        -893.682158
Standard orientation:
```

Center Number	Atomic Number	Atomic Type	Coordinates (Angstroms) X	Y	Z
1	6	0	3.846533	-0.387801	0.295514
2	6	0	2.473455	-0.125728	-0.305979
3	8	0	2.364740	0.627653	-1.274873
4	7	0	1.427660	-0.746714	0.254785
5	6	0	0.031608	-0.555374	-0.141901
6	6	0	-0.478306	0.802191	0.387840
7	8	0	-0.194289	1.151947	1.536440
8	6	0	-0.840734	-1.738928	0.342423
9	16	0	-2.539729	-1.691680	-0.314875
10	7	0	-1.232780	1.528208	-0.443693
11	6	0	-1.812845	2.829879	-0.122544
12	1	0	3.801012	-1.090383	1.127441
13	1	0	4.514772	-0.800801	-0.459781
14	1	0	4.279354	0.542858	0.661434
15	1	0	1.575594	-1.318161	1.074664
16	1	0	-0.001839	-0.540385	-1.233739
17	1	0	-0.391012	-2.683486	0.034019
18	1	0	-0.886650	-1.756470	1.432953
19	1	0	-2.950705	-0.642555	0.412496
20	1	0	-1.425004	1.158622	-1.366726
21	1	0	-1.471698	3.578339	-0.838124
22	1	0	-2.900217	2.781113	-0.185015
23	1	0	-1.553985	3.185506	0.876071

Ac-Cys-NHMe β-4: M06-2X/6-31+G(d), scrf=(smd, solvent=water)

NIMG=1 (41.5851 cm^{-1})

```
Zero-point correction=                             0.189123 (Hartree/Particle)
 Thermal correction to Energy=                     0.202391
 Thermal correction to Enthalpy=                   0.203335
 Thermal correction to Gibbs Free Energy=          0.147942
 Sum of electronic and zero-point Energies=          -893.639700
 Sum of electronic and thermal Energies=             -893.626431
 Sum of electronic and thermal Enthalpies=           -893.625487
 Sum of electronic and thermal Free Energies=        -893.680881
Standard orientation:
```

Center Number	Atomic Number	Atomic Type	Coordinates (Angstroms) X	Y	Z
1	6	0	3.804317	-0.938776	-0.173207
2	6	0	2.589065	-0.129614	0.255496
3	8	0	2.730690	0.838967	1.003308
4	7	0	1.397945	-0.517381	-0.209690
5	6	0	0.114309	0.098472	0.107012
6	6	0	-0.957346	-0.980378	-0.138022
7	8	0	-0.750957	-1.856946	-0.983329
8	6	0	-0.120990	1.386813	-0.723140
9	16	0	-1.573423	2.338356	-0.172088
10	7	0	-2.068923	-0.910902	0.600492
11	6	0	-3.189630	-1.843509	0.512010
12	1	0	3.533782	-1.760696	-0.835866
13	1	0	4.514088	-0.300080	-0.698409
14	1	0	4.303624	-1.357608	0.700075
15	1	0	1.304189	-1.343778	-0.790433
16	1	0	0.111709	0.346694	1.170778
17	1	0	0.746977	2.043339	-0.651810
18	1	0	-0.235751	1.140458	-1.779962
19	1	0	-1.021138	2.804553	0.957680
20	1	0	-2.165018	-0.140694	1.250983
21	1	0	-4.087804	-1.319226	0.184951
22	1	0	-3.014392	-2.666792	-0.182347
23	1	0	-3.394868	-2.276102	1.491421

Ac-Cys-NHMe β-3: M06-2X/6-31+G(d), scrf=(smd, solvent=water)

NIMG=1 (27.7403 cm^{-1})

```
  Zero-point correction=                           0.188841 (Hartree/Particle)
 Thermal correction to Energy=                    0.202065
 Thermal correction to Enthalpy=                  0.203009
 Thermal correction to Gibbs Free Energy=          0.147505
 Sum of electronic and zero-point Energies=          -893.640916
 Sum of electronic and thermal Energies=             -893.627691
 Sum of electronic and thermal Enthalpies=           -893.626747
 Sum of electronic and thermal Free Energies=        -893.682252
Standard orientation:
```

Center Number	Atomic Number	Atomic Type	Coordinates (Angstroms) X	Y	Z
1	6	0	3.396536	-1.118233	-0.820034
2	6	0	2.353826	-0.682684	0.198999
3	8	0	2.688552	-0.471122	1.365551
4	7	0	1.095641	-0.549556	-0.230084
5	6	0	-0.054797	-0.174485	0.586991
6	6	0	-1.327712	-0.658072	-0.132288
7	8	0	-1.265461	-1.024576	-1.310593
8	6	0	-0.068728	1.338405	0.929835
9	16	0	-0.194634	2.408874	-0.541662
10	7	0	-2.454035	-0.661653	0.588084
11	6	0	-3.760639	-1.081359	0.086923
12	1	0	2.963286	-1.264182	-1.809419
13	1	0	4.179465	-0.364583	-0.901206
14	1	0	3.856386	-2.056005	-0.509604
15	1	0	0.836673	-0.782458	-1.182520
16	1	0	0.006954	-0.735642	1.522406
17	1	0	-0.910355	1.559724	1.587692
18	1	0	0.826188	1.607999	1.492036
19	1	0	1.022047	2.120213	-1.025105
20	1	0	-2.415549	-0.349942	1.550655

21	1	0	-4.469787	-0.255910	0.153191
22	1	0	-3.740080	-1.416805	-0.951202
23	1	0	-4.147968	-1.901772	0.691439

Ac-Cys-NHMe β-2: M06-2X/6-31+G(d), scrf=(smd, solvent=chloroform)

```
NIMG=1 (26.4152 cm-1)
Zero-point correction=                           0.188595 (Hartree/Particle)
 Thermal correction to Energy=                    0.202033
 Thermal correction to Enthalpy=                  0.202977
 Thermal correction to Gibbs Free Energy=         0.146928
 Sum of electronic and zero-point Energies=          -893.633020
 Sum of electronic and thermal Energies=             -893.619582
 Sum of electronic and thermal Enthalpies=           -893.618638
 Sum of electronic and thermal Free Energies=        -893.674687
Standard orientation:
```

Center Number	Atomic Number	Atomic Type	Coordinates (Angstroms) X	Y	Z
1	6	0	-2.743572	-2.303050	0.137141
2	6	0	-1.969745	-1.052641	-0.252038
3	8	0	-2.514057	-0.189838	-0.935487
4	7	0	-0.709867	-0.949165	0.180172
5	6	0	0.230967	0.126192	-0.115180
6	6	0	1.630464	-0.468288	0.098496
7	8	0	1.761590	-1.462234	0.815996
8	6	0	0.022647	1.365066	0.788397
9	16	0	-1.254823	2.455995	0.097449
10	7	0	2.642806	0.150792	-0.516985
11	6	0	4.035708	-0.275969	-0.421054
12	1	0	-3.630806	-2.025079	0.705672
13	1	0	-3.066229	-2.830790	-0.760059
14	1	0	-2.145731	-2.984329	0.742099
15	1	0	-0.260684	-1.696433	0.698772
16	1	0	0.140234	0.401117	-1.168404
17	1	0	-0.238607	1.072564	1.806178
18	1	0	0.938744	1.953382	0.861536
19	1	0	-2.094676	1.458054	-0.247184
20	1	0	2.444876	0.958297	-1.090153
21	1	0	4.182669	-1.113169	0.264175
22	1	0	4.401633	-0.581671	-1.401542
23	1	0	4.656102	0.549460	-0.071228

Ac-Cys-NHMe β-1: M06-2X/6-31+G(d), scrf=(smd, solvent=chloroform)

```
NIMG=1 (44.8445 cm-1)
  Zero-point correction=                         0.189049 (Hartree/Particle)
 Thermal correction to Energy=                    0.202220
 Thermal correction to Enthalpy=                  0.203165
 Thermal correction to Gibbs Free Energy=         0.148660
 Sum of electronic and zero-point Energies=          -893.633890
 Sum of electronic and thermal Energies=             -893.620719
 Sum of electronic and thermal Enthalpies=           -893.619774
 Sum of electronic and thermal Free Energies=        -893.674279
Standard orientation:
```

Center Number	Atomic Number	Atomic Type	Coordinates (Angstroms) X	Y	Z
1	6	0	-3.794997	-0.980137	0.140305
2	6	0	-2.582734	-0.121419	-0.187269

3	8	0	-2.736291	0.967302	-0.734894
4	7	0	-1.379566	-0.599448	0.141921
5	6	0	-0.104793	0.045932	-0.139597
6	6	0	0.970334	-1.047786	0.014029
7	8	0	0.654957	-2.146819	0.478157
8	6	0	0.145974	1.277366	0.769445
9	16	0	1.436250	2.376540	0.102949
10	7	0	2.209566	-0.736180	-0.373271
11	6	0	3.345138	-1.649243	-0.304855
12	1	0	-3.515585	-1.916152	0.623438
13	1	0	-4.464153	-0.434699	0.805324
14	1	0	-4.339554	-1.213004	-0.774426
15	1	0	-1.248720	-1.525434	0.536622
16	1	0	-0.122145	0.358771	-1.185725
17	1	0	-0.762573	1.873599	0.862773
18	1	0	0.417011	0.964482	1.778964
19	1	0	0.668035	2.945079	-0.834957
20	1	0	2.385152	0.215975	-0.669199
21	1	0	4.038024	-1.325767	0.472308
22	1	0	3.052350	-2.678023	-0.086255
23	1	0	3.879300	-1.652231	-1.255057

Ac-Phe-NHMe PPII-2: M06-2X/6-31+G(d), scrf=(smd, solvent=water)

NIMG=1 (30.3609 cm^{-1})
Zero-point correction= 0.270924 (Hartree/Particle)
Thermal correction to Energy= 0.287314
Thermal correction to Enthalpy= 0.288258
Thermal correction to Gibbs Free Energy= 0.224900
Sum of electronic and zero-point Energies= -726.371240
Sum of electronic and thermal Energies= -726.354850
Sum of electronic and thermal Enthalpies= -726.353906
Sum of electronic and thermal Free Energies= -726.417264
Standard orientation:

Center Number	Atomic Number	Atomic Type	Coordinates (Angstroms) X	Y	Z
1	6	0	0.706640	3.619598	-0.007951
2	6	0	0.994028	2.204381	0.471710
3	8	0	1.625123	2.028896	1.515519
4	7	0	0.527878	1.197592	-0.277009
5	6	0	0.749945	-0.223965	0.000959
6	6	0	2.193181	-0.642513	-0.332475
7	8	0	2.785948	-0.109319	-1.273624
8	6	0	-0.256975	-1.076322	-0.802966
9	6	0	-1.714961	-0.868092	-0.425883
10	6	0	-2.512486	0.061507	-1.128309
11	6	0	-2.240751	-1.486218	0.728990
12	6	0	-3.815750	0.308722	-0.716952
13	6	0	-3.546579	-1.225062	1.123261
14	6	0	-4.332153	-0.335613	0.400517
15	7	0	2.715905	-1.614016	0.421764
16	6	0	4.059092	-2.165048	0.257612
17	1	0	0.122475	3.628083	-0.928038
18	1	0	0.149306	4.167450	0.751453
19	1	0	1.640458	4.149306	-0.194512
20	1	0	0.039259	1.404004	-1.135819
21	1	0	0.578563	-0.396407	1.065944

22	1	0	-0.131394	-0.899134	-1.872701
23	1	0	-0.024104	-2.133368	-0.659466
24	1	0	-2.119589	0.580792	-1.992186
25	1	0	-1.633203	-2.166896	1.310612
26	1	0	-4.429137	1.011144	-1.265274
27	1	0	-3.951080	-1.710415	2.001464
28	1	0	-5.347497	-0.135408	0.715870
29	1	0	2.153975	-2.001503	1.169844
30	1	0	4.003396	-3.237583	0.070076
31	1	0	4.615420	-1.713782	-0.565646
32	1	0	4.638723	-2.016188	1.168854

Ac-Phe-NHMe PPII-1: M06-2X/6-31+G(d), scrf=(smd, solvent=water)

NIMG=1 (27.1690 cm^{-1})
Zero-point correction= 0.270280 (Hartree/Particle)
Thermal correction to Energy= 0.286663
Thermal correction to Enthalpy= 0.287607
Thermal correction to Gibbs Free Energy= 0.224530
Sum of electronic and zero-point Energies= -726.372843
Sum of electronic and thermal Energies= -726.356461
Sum of electronic and thermal Enthalpies= -726.355517
Sum of electronic and thermal Free Energies= -726.418594
Standard orientation:

Center Number	Atomic Number	Atomic Type	Coordinates (Angstroms) X	Y	Z
1	6	0	-4.669212	-1.032865	-0.281491
2	6	0	-3.345876	-0.586573	0.322813
3	8	0	-3.334252	-0.017611	1.415704
4	7	0	-2.232800	-0.847822	-0.374510
5	6	0	-0.885932	-0.422664	0.015465
6	6	0	-0.694174	1.084974	-0.243878
7	8	0	-1.214963	1.605923	-1.233508
8	6	0	0.150102	-1.292768	-0.736331
9	6	0	1.609640	-1.025549	-0.401415
10	6	0	2.377145	-0.138976	-1.187979
11	6	0	2.164791	-1.539219	0.790181
12	6	0	3.679692	0.168210	-0.817278
13	6	0	3.469514	-1.219615	1.142663
14	6	0	4.225144	-0.374096	0.339807
15	7	0	0.064319	1.748802	0.633368
16	6	0	0.390607	3.169291	0.548061
17	1	0	-4.532302	-1.536973	-1.238056
18	1	0	-5.177519	-1.720774	0.393762
19	1	0	-5.317344	-0.171711	-0.442319
20	1	0	-2.312465	-1.279243	-1.284724
21	1	0	-0.769018	-0.607357	1.085962
22	1	0	-0.045630	-2.343577	-0.517887
23	1	0	0.015582	-1.183831	-1.813892
24	1	0	1.956545	0.306461	-2.079887
25	1	0	1.580430	-2.184684	1.432740
26	1	0	4.268978	0.839161	-1.427849
27	1	0	3.896642	-1.624939	2.050219
28	1	0	5.239737	-0.127361	0.623254
29	1	0	0.479738	1.229953	1.397075
30	1	0	1.457399	3.296369	0.362668
31	1	0	-0.150051	3.689656	-0.244120

32	1	0	0.153727	3.664598	1.489773

Ac-Phe-NHMe PPII-3: M06-2X/6-31+G(d), scrf=(smd, solvent=water)

NIMG=1 (29.5706 cm^{-1})

```
  Zero-point correction=                           0.270753 (Hartree/Particle)
 Thermal correction to Energy=                    0.286923
 Thermal correction to Enthalpy=                  0.287867
 Thermal correction to Gibbs Free Energy=         0.225423
 Sum of electronic and zero-point Energies=          -726.371661
 Sum of electronic and thermal Energies=             -726.355491
 Sum of electronic and thermal Enthalpies=           -726.354547
 Sum of electronic and thermal Free Energies=        -726.416991
```

Standard orientation:

Center Number	Atomic Number	Atomic Type	Coordinates (Angstroms) X	Y	Z
1	6	0	-1.928166	-3.253526	-0.863685
2	6	0	-2.115135	-1.939220	-0.119962
3	8	0	-3.250681	-1.548613	0.156714
4	7	0	-1.010069	-1.261941	0.212480
5	6	0	-0.991485	0.058572	0.844295
6	6	0	-1.248915	1.184490	-0.176652
7	8	0	-1.086042	0.983517	-1.382869
8	6	0	0.338025	0.239417	1.619390
9	6	0	1.627067	0.072993	0.824752
10	6	0	2.161800	1.153098	0.089364
11	6	0	2.221237	-1.201704	0.696196
12	6	0	3.290189	0.967042	-0.698673
13	6	0	3.348820	-1.366406	-0.097517
14	6	0	3.883733	-0.285930	-0.787771
15	7	0	-1.635782	2.358453	0.332547
16	6	0	-1.915681	3.556831	-0.454999
17	1	0	-0.875324	-3.483464	-1.027231
18	1	0	-2.369421	-4.072523	-0.296189
19	1	0	-2.419599	-3.207652	-1.835229
20	1	0	-0.106902	-1.597658	-0.092508
21	1	0	-1.802051	0.097704	1.575570
22	1	0	0.352644	1.233560	2.069581
23	1	0	0.355614	-0.449120	2.465453
24	1	0	1.698276	2.129846	0.135180
25	1	0	1.810381	-2.054080	1.221242
26	1	0	3.703639	1.799944	-1.251537
27	1	0	3.809687	-2.341567	-0.182494
28	1	0	4.761035	-0.423039	-1.405924
29	1	0	-1.749279	2.437690	1.335616
30	1	0	-1.292203	4.383012	-0.112468
31	1	0	-1.732940	3.426469	-1.522805
32	1	0	-2.957747	3.852120	-0.330644

Ac-Phe-NHMe β-4: M06-2X/6-31+G(d), scrf=(smd, solvent=water)

NIMG=1 (31.1151 cm^{-1})

```
 Zero-point correction=                           0.270675 (Hartree/Particle)
 Thermal correction to Energy=                    0.286874
 Thermal correction to Enthalpy=                  0.287818
 Thermal correction to Gibbs Free Energy=         0.225095
```

```
 Sum of electronic and zero-point Energies=         -726.371277
 Sum of electronic and thermal Energies=            -726.355078
 Sum of electronic and thermal Enthalpies=          -726.354134
 Sum of electronic and thermal Free Energies=       -726.416857
Standard orientation:
 ---------------------------------------------------------------------
 Center     Atomic      Atomic             Coordinates (Angstroms)
 Number     Number       Type             X           Y           Z
 ---------------------------------------------------------------------
      1          6           0        4.721009   -0.678265    0.280735
      2          6           0        3.278453   -0.863724   -0.166171
      3          8           0        2.971440   -1.836635   -0.856679
      4          7           0        2.393292    0.059949    0.220308
      5          6           0        0.976568    0.090795   -0.132222
      6          6           0        0.516811    1.554555   -0.044978
      7          8           0        1.120354    2.337006    0.695090
      8          6           0        0.182406   -0.860323    0.799496
      9          6           0       -1.307586   -0.974258    0.518194
     10          6           0       -2.236775   -0.186517    1.232029
     11          6           0       -1.761732   -1.756326   -0.565113
     12          6           0       -3.584766   -0.233167    0.901409
     13          6           0       -3.114206   -1.788928   -0.878950
     14          6           0       -4.021987   -1.034692   -0.145949
     15          7           0       -0.530552    1.899669   -0.798227
     16          6           0       -1.123526    3.233106   -0.844496
     17          1           0        5.375704   -0.598257   -0.586802
     18          1           0        4.848133    0.219770    0.885282
     19          1           0        5.043207   -1.533867    0.873668
     20          1           0        2.687145    0.876583    0.745981
     21          1           0        0.864597   -0.239455   -1.167396
     22          1           0        0.602703   -1.864405    0.728073
     23          1           0        0.319816   -0.557798    1.839016
     24          1           0       -1.906205    0.457396    2.036250
     25          1           0       -1.061288   -2.334390   -1.153730
     26          1           0       -4.296072    0.363500    1.456811
     27          1           0       -3.461015   -2.398765   -1.702547
     28          1           0       -5.073555   -1.063221   -0.398850
     29          1           0       -0.984150    1.177994   -1.345158
     30          1           0       -1.167673    3.587468   -1.874446
     31          1           0       -2.142625    3.201043   -0.458490
     32          1           0       -0.570270    3.972567   -0.263420
 ---------------------------------------------------------------------
```

Ac-Phe-NHMe β-3: M06-2X/6-31+G(d), scrf=(smd, solvent=water)

NIMG=1 (27.5898 cm^{-1})

```
   Zero-point correction=                           0.271157 (Hartree/Particle)
 Thermal correction to Energy=                      0.287131
 Thermal correction to Enthalpy=                    0.288075
 Thermal correction to Gibbs Free Energy=           0.226292
 Sum of electronic and zero-point Energies=         -726.371380
 Sum of electronic and thermal Energies=            -726.355406
 Sum of electronic and thermal Enthalpies=          -726.354462
 Sum of electronic and thermal Free Energies=       -726.416245
Standard orientation:
 ---------------------------------------------------------------------
 Center     Atomic      Atomic             Coordinates (Angstroms)
 Number     Number       Type             X           Y           Z
 ---------------------------------------------------------------------
      1          6           0       -3.487991   -1.585306   -1.100422
```

2	6	0	-2.831388	-0.755920	-0.006937
3	8	0	-3.311081	-0.747757	1.127784
4	7	0	-1.740178	-0.060257	-0.338939
5	6	0	-0.971771	0.812368	0.545588
6	6	0	-0.215302	1.824234	-0.330178
7	8	0	-0.151294	1.655121	-1.551753
8	6	0	-0.044790	-0.024511	1.469370
9	6	0	1.069508	-0.820752	0.800939
10	6	0	2.335151	-0.235941	0.576355
11	6	0	0.805234	-2.101119	0.268434
12	6	0	3.316865	-0.942995	-0.105663
13	6	0	1.800398	-2.791526	-0.411069
14	6	0	3.052331	-2.217168	-0.592304
15	7	0	0.334819	2.861444	0.307984
16	6	0	1.101910	3.923457	-0.338484
17	1	0	-2.953103	-1.507379	-2.047008
18	1	0	-3.512165	-2.635619	-0.811130
19	1	0	-4.512714	-1.251574	-1.261289
20	1	0	-1.398616	-0.044989	-1.293867
21	1	0	-1.672919	1.376021	1.164933
22	1	0	0.414545	0.643440	2.199627
23	1	0	-0.649661	-0.709315	2.065070
24	1	0	2.548548	0.762693	0.934330
25	1	0	-0.171128	-2.551291	0.389663
26	1	0	4.289080	-0.496207	-0.265151
27	1	0	1.597141	-3.777615	-0.806707
28	1	0	3.821552	-2.760239	-1.125203
29	1	0	0.222405	2.928030	1.311927
30	1	0	2.087336	4.004685	0.120726
31	1	0	1.249143	3.762048	-1.407574
32	1	0	0.596140	4.881177	-0.213676

Ac-Phe-NHMe β-1: M06-2X/6-31+G(d), scrf=(smd, solvent=chloroform)

```
NIMG=1 (23.1358 cm-1)
Zero-point correction=                           0.270400 (Hartree/Particle)
 Thermal correction to Energy=                     0.286893
 Thermal correction to Enthalpy=                   0.287838
 Thermal correction to Gibbs Free Energy=          0.223775
 Sum of electronic and zero-point Energies=          -726.370343
 Sum of electronic and thermal Energies=             -726.353849
 Sum of electronic and thermal Enthalpies=           -726.352905
 Sum of electronic and thermal Free Energies=        -726.416968
Standard orientation:
```

Center Number	Atomic Number	Atomic Type	Coordinates (Angstroms) X	Y	Z
1	6	0	4.697262	-0.733831	0.233392
2	6	0	3.234041	-0.913290	-0.141375
3	8	0	2.872148	-1.940272	-0.709784
4	7	0	2.391561	0.075583	0.171661
5	6	0	0.970553	0.114111	-0.150308
6	6	0	0.553474	1.591122	-0.088268
7	8	0	1.276034	2.398605	0.500457
8	6	0	0.179986	-0.791922	0.826734
9	6	0	-1.302382	-0.943385	0.526200
10	6	0	-1.722665	-1.773639	-0.534467
11	6	0	-2.258498	-0.150255	1.197209
12	6	0	-3.068531	-1.844237	-0.869777
13	6	0	-3.599589	-0.234978	0.845874

14	6	0	-4.003024	-1.081653	-0.179531
15	7	0	-0.590171	1.917074	-0.695114
16	6	0	-1.132903	3.269918	-0.745055
17	1	0	4.878521	0.218519	0.731058
18	1	0	5.007316	-1.537083	0.901282
19	1	0	5.315083	-0.778369	-0.663116
20	1	0	2.704920	0.947102	0.585845
21	1	0	0.837548	-0.241577	-1.174124
22	1	0	0.612133	-1.793566	0.816850
23	1	0	0.299578	-0.428801	1.848597
24	1	0	-0.999743	-2.360111	-1.085301
25	1	0	-1.952750	0.525926	1.983521
26	1	0	-3.389401	-2.492147	-1.673156
27	1	0	-4.331575	0.363567	1.369239
28	1	0	-5.048689	-1.142215	-0.446589
29	1	0	-1.149143	1.171592	-1.088297
30	1	0	-2.047677	3.325377	-0.154488
31	1	0	-0.440719	4.022697	-0.362754
32	1	0	-1.376934	3.536065	-1.773568

Ac-Phe-NHMe β-3: M06-2X/6-31+G(d), scrf=(smd, solvent=chloroform)

```
NIMG=1 (23.0340 cm-1)
  Zero-point correction=                           0.270579 (Hartree/Particle)
 Thermal correction to Energy=                    0.286890
 Thermal correction to Enthalpy=                  0.287834
 Thermal correction to Gibbs Free Energy=          0.224803
 Sum of electronic and zero-point Energies=           -726.369367
 Sum of electronic and thermal Energies=              -726.353056
 Sum of electronic and thermal Enthalpies=            -726.352112
 Sum of electronic and thermal Free Energies=         -726.415144
Standard orientation:
```

Center Number	Atomic Number	Atomic Type	Coordinates (Angstroms) X	Y	Z
1	6	0	-3.420069	-1.612283	-1.135310
2	6	0	-2.763135	-0.808898	-0.023203
3	8	0	-3.205760	-0.876366	1.120761
4	7	0	-1.711674	-0.052356	-0.348531
5	6	0	-0.976161	0.819432	0.558775
6	6	0	-0.244975	1.857293	-0.304320
7	8	0	-0.203339	1.713665	-1.528160
8	6	0	-0.036223	-0.006535	1.477689
9	6	0	1.069224	-0.807438	0.800987
10	6	0	2.314535	-0.211319	0.504101
11	6	0	0.808759	-2.112321	0.330156
12	6	0	3.282525	-0.928943	-0.186652
13	6	0	1.789666	-2.813830	-0.358658
14	6	0	3.023277	-2.226353	-0.611003
15	7	0	0.304357	2.887990	0.345487
16	6	0	1.036204	3.961810	-0.318275
17	1	0	-2.915933	-1.473537	-2.091499
18	1	0	-3.399442	-2.672568	-0.885089
19	1	0	-4.460301	-1.307436	-1.245024
20	1	0	-1.376538	0.038257	-1.301559
21	1	0	-1.700735	1.353613	1.176674
22	1	0	0.432781	0.663656	2.198576
23	1	0	-0.635516	-0.689547	2.081311
24	1	0	2.521066	0.804484	0.808788
25	1	0	-0.154475	-2.569681	0.507521

26	1	0	4.237701	-0.472510	-0.403588
27	1	0	1.589706	-3.817344	-0.705926
28	1	0	3.780427	-2.776819	-1.151072
29	1	0	0.203691	2.943071	1.347655
30	1	0	1.313409	3.715292	-1.345354
31	1	0	0.431724	4.868900	-0.343009
32	1	0	1.954680	4.180634	0.226378

Ac-Tyr-NHMe PPII-2: M06-2X/6-31+G(d), scrf=(smd, solvent=water)

NIMG=1 (32.3562 cm^{-1})

```
  Zero-point correction=                           0.275148 (Hartree/Particle)
 Thermal correction to Energy=                     0.292634
 Thermal correction to Enthalpy=                   0.293578
 Thermal correction to Gibbs Free Energy=          0.227948
 Sum of electronic and zero-point Energies=         -801.572517
 Sum of electronic and thermal Energies=            -801.555032
 Sum of electronic and thermal Enthalpies=          -801.554088
 Sum of electronic and thermal Free Energies=       -801.619718
Standard orientation:
```

Center Number	Atomic Number	Atomic Type	Coordinates (Angstroms) X	Y	Z
1	6	0	-1.028705	3.595395	0.115243
2	6	0	-1.366988	2.208489	-0.412208
3	8	0	-2.052387	2.092000	-1.429916
4	7	0	-0.882578	1.161216	0.266994
5	6	0	-1.138423	-0.243495	-0.062361
6	6	0	-2.582350	-0.650558	0.283645
7	8	0	-3.152116	-0.138081	1.249911
8	6	0	-0.132533	-1.138089	0.694383
9	6	0	1.330280	-0.842602	0.402332
10	6	0	2.135214	-0.199032	1.366506
11	6	0	1.889690	-1.211565	-0.839187
12	6	0	3.488056	0.075786	1.087018
13	6	0	3.242942	-0.936273	-1.116690
14	6	0	4.043002	-0.291804	-0.153331
15	8	0	5.353669	-0.020863	-0.411516
16	7	0	-3.130902	-1.592739	-0.489389
17	6	0	-4.478823	-2.130094	-0.318528
18	1	0	-0.491999	4.164885	-0.643193
19	1	0	-1.941944	4.134776	0.365049
20	1	0	-0.406050	3.550833	1.008668
21	1	0	-0.337318	1.320512	1.101339
22	1	0	-0.987023	-0.373810	-1.136166
23	1	0	-0.307089	-1.064552	1.769646
24	1	0	-0.319619	-2.184641	0.445587
25	1	0	1.723306	0.088302	2.324396
26	1	0	1.283315	-1.706032	-1.586042
27	1	0	4.097060	0.570171	1.831575
28	1	0	3.652814	-1.226095	-2.074751
29	1	0	5.634900	-0.315795	-1.290753
30	1	0	-2.586055	-1.967888	-1.256287
31	1	0	-4.432471	-3.198630	-0.107055
32	1	0	-5.035451	-1.655736	0.491361
33	1	0	-5.053509	-1.997040	-1.235380

Ac-Tyr-NHMe PPII-4: M06-2X/6-31+G(d), scrf=(smd, solvent=water)

```
NIMG=1 (27.4489 cm-1)
 Zero-point correction=                           0.274518 (Hartree/Particle)
 Thermal correction to Energy=                     0.291964
 Thermal correction to Enthalpy=                   0.292908
 Thermal correction to Gibbs Free Energy=          0.227699
 Sum of electronic and zero-point Energies=          -801.574105
 Sum of electronic and thermal Energies=             -801.556660
 Sum of electronic and thermal Enthalpies=           -801.555716
 Sum of electronic and thermal Free Energies=        -801.620925
Standard orientation:
 ---------------------------------------------------------------------
 Center     Atomic      Atomic             Coordinates (Angstroms)
 Number     Number       Type             X           Y           Z
 ---------------------------------------------------------------------
      1          6           0        5.004527   -1.168083    0.180591
      2          6           0        3.683890   -0.666349   -0.385089
      3          8           0        3.664479   -0.097300   -1.478164
      4          7           0        2.582114   -0.881100    0.345849
      5          6           0        1.242394   -0.403850   -0.005242
      6          6           0        1.119908    1.112247    0.246759
      7          8           0        1.703494    1.622909    1.206010
      8          6           0        0.199284   -1.215855    0.798069
      9          6           0       -1.255754   -0.920039    0.469156
     10          6           0       -2.039022   -0.132322    1.339460
     11          6           0       -1.827805   -1.430240   -0.715243
     12          6           0       -3.383592    0.144191    1.024038
     13          6           0       -3.172783   -1.152857   -1.028813
     14          6           0       -3.951459   -0.365071   -0.158862
     15          8           0       -5.253875   -0.090544   -0.453066
     16          7           0        0.345494    1.795740   -0.601152
     17          6           0        0.078537    3.228820   -0.516937
     18          1           0        5.461485   -1.879713   -0.506537
     19          1           0        5.693887   -0.335407    0.318209
     20          1           0        4.874208   -1.662582    1.143111
     21          1           0        2.670175   -1.320258    1.251256
     22          1           0        1.082499   -0.595563   -1.068693
     23          1           0        0.359818   -2.281517    0.626997
     24          1           0        0.356621   -1.062425    1.867375
     25          1           0       -1.613277    0.269124    2.249130
     26          1           0       -1.237846   -2.035727   -1.390163
     27          1           0       -3.975423    0.750760    1.695980
     28          1           0       -3.592982   -1.552429   -1.941779
     29          1           0       -5.545292   -0.488578   -1.287215
     30          1           0       -0.121246    1.286993   -1.341669
     31          1           0        0.263105    3.700896   -1.482068
     32          1           0       -0.966230    3.399756   -0.256025
     33          1           0        0.695712    3.740093    0.223659
 ---------------------------------------------------------------------
```

Ac-Tyr-NHMe β-1: M06-2X/6-31+G(d), scrf=(smd, solvent=water)

```
NIMG=1 (19.1463 cm-1)
Zero-point correction=                           0.274711 (Hartree/Particle)
 Thermal correction to Energy=                     0.292182
 Thermal correction to Enthalpy=                   0.293126
 Thermal correction to Gibbs Free Energy=          0.226806
 Sum of electronic and zero-point Energies=          -801.572664
```

```
 Sum of electronic and thermal Energies=           -801.555193
 Sum of electronic and thermal Enthalpies=          -801.554249
 Sum of electronic and thermal Free Energies=       -801.620569
Standard orientation:
```

Center Number	Atomic Number	Atomic Type	Coordinates (Angstroms) X	Y	Z
1	6	0	4.991696	-1.064799	0.234734
2	6	0	3.531658	-1.101064	-0.192549
3	8	0	3.116644	-2.039306	-0.874992
4	7	0	2.752829	-0.088685	0.202230
5	6	0	1.341794	0.084508	-0.131007
6	6	0	1.028140	1.587178	-0.054679
7	8	0	1.720404	2.317818	0.659560
8	6	0	0.468711	-0.765544	0.826182
9	6	0	-1.024617	-0.751328	0.540269
10	6	0	-1.903540	-0.017552	1.365091
11	6	0	-1.537039	-1.471772	-0.558991
12	6	0	-3.284406	-0.002933	1.088204
13	6	0	-2.918450	-1.456153	-0.834001
14	6	0	-3.793004	-0.721419	-0.010137
15	8	0	-5.131432	-0.699630	-0.267604
16	7	0	-0.001769	2.021157	-0.786153
17	6	0	-0.465559	3.404843	-0.837992
18	1	0	5.231138	-1.947262	0.827484
19	1	0	5.639400	-1.055724	-0.641580
20	1	0	5.218659	-0.182707	0.833364
21	1	0	3.139221	0.691053	0.722524
22	1	0	1.180881	-0.243570	-1.160427
23	1	0	0.792318	-1.806684	0.788185
24	1	0	0.636681	-0.444913	1.855818
25	1	0	-1.525012	0.542446	2.209544
26	1	0	-0.872615	-2.038758	-1.197264
27	1	0	-3.950138	0.564251	1.724489
28	1	0	-3.292113	-2.014190	-1.681923
29	1	0	-5.376122	-1.227101	-1.042788
30	1	0	-0.532683	1.340642	-1.315781
31	1	0	-1.471706	3.476722	-0.424265
32	1	0	0.172222	4.095782	-0.284404
33	1	0	-0.504788	3.747853	-1.871966

Ac-Tyr-NHMe β-3: M06-2X/6-31+G(d), scrf=(smd, solvent=water)

NIMG=1 (14.5463 cm^{-1})

```
 Zero-point correction=                           0.274673 (Hartree/Particle)
 Thermal correction to Energy=                     0.291947
 Thermal correction to Enthalpy=                   0.292891
 Thermal correction to Gibbs Free Energy=          0.227938
 Sum of electronic and zero-point Energies=           -801.573406
 Sum of electronic and thermal Energies=           -801.556132
 Sum of electronic and thermal Enthalpies=          -801.555188
 Sum of electronic and thermal Free Energies=       -801.620141
Standard orientation:
```

Center Number	Atomic Number	Atomic Type	Coordinates (Angstroms) X	Y	Z
1	6	0	2.243113	-3.203246	1.162779

2	6	0	2.244521	-2.134325	0.079975
3	8	0	2.724362	-2.384418	-1.027050
4	7	0	1.711313	-0.948970	0.391096
5	6	0	1.628640	0.212909	-0.490648
6	6	0	1.526786	1.474643	0.381130
7	8	0	1.283765	1.372766	1.587124
8	6	0	0.448943	0.060055	-1.487764
9	6	0	-0.946444	-0.052998	-0.886433
10	6	0	-1.734084	1.101767	-0.690882
11	6	0	-1.465443	-1.316797	-0.533182
12	6	0	-3.025611	0.992182	-0.139922
13	6	0	-2.757172	-1.425028	0.018086
14	6	0	-3.537949	-0.269652	0.215377
15	8	0	-4.789397	-0.362943	0.747789
16	7	0	1.718913	2.640412	-0.243609
17	6	0	1.654615	3.950006	0.400625
18	1	0	1.691386	-4.079720	0.823907
19	1	0	3.263944	-3.508786	1.390685
20	1	0	1.781372	-2.848080	2.084165
21	1	0	1.367975	-0.761395	1.326123
22	1	0	2.561008	0.284223	-1.055157
23	1	0	0.453540	0.908033	-2.174569
24	1	0	0.622014	-0.809315	-2.123530
25	1	0	-1.351017	2.077486	-0.957830
26	1	0	-0.873370	-2.209584	-0.681602
27	1	0	-3.619904	1.883563	0.008343
28	1	0	-3.135065	-2.402860	0.284131
29	1	0	-5.045087	-1.274293	0.955257
30	1	0	1.922217	2.631133	-1.235543
31	1	0	0.906280	4.571946	-0.091003
32	1	0	1.397686	3.900133	1.459986
33	1	0	2.616101	4.457105	0.316476

Ac-Tyr-NHMe β-2: M06-2X/6-31+G(d), scrf=(smd, solvent=chloroform)

NIMG=1 (35.0612 cm^{-1})
Zero-point correction= 0.275007 (Hartree/Particle)
Thermal correction to Energy= 0.292504
Thermal correction to Enthalpy= 0.293448
Thermal correction to Gibbs Free Energy= 0.227929
Sum of electronic and zero-point Energies= -801.563335
Sum of electronic and thermal Energies= -801.545838
Sum of electronic and thermal Enthalpies= -801.544894
Sum of electronic and thermal Free Energies= -801.610413
Standard orientation:

Center Number	Atomic Number	Atomic Type	Coordinates (Angstroms) X	Y	Z
1	6	0	-0.396652	3.488792	0.749953
2	6	0	-0.576122	2.242462	-0.103941
3	8	0	-0.297511	2.282701	-1.300539
4	7	0	-1.045403	1.146134	0.502601
5	6	0	-1.355738	-0.113088	-0.167936
6	6	0	-2.805103	-0.469759	0.177916
7	8	0	-3.258502	-0.146420	1.276341
8	6	0	-0.407397	-1.229896	0.330072
9	6	0	1.087955	-0.982820	0.173259
10	6	0	1.616480	-0.491779	-1.040747

11	6	0	1.962999	-1.263625	1.245033
12	6	0	3.001661	-0.276360	-1.178906
13	6	0	3.348139	-1.050304	1.105083
14	6	0	3.868027	-0.555856	-0.104658
15	8	0	5.209834	-0.350674	-0.224639
16	7	0	-3.488954	-1.151493	-0.745975
17	6	0	-4.873391	-1.582825	-0.578324
18	1	0	-0.697075	3.323221	1.784170
19	1	0	0.649892	3.792374	0.739027
20	1	0	-0.993415	4.304232	0.342377
21	1	0	-1.369330	1.182207	1.458624
22	1	0	-1.275557	-0.008214	-1.251303
23	1	0	-0.616360	-1.427968	1.383132
24	1	0	-0.640776	-2.159236	-0.191515
25	1	0	0.963039	-0.260009	-1.869469
26	1	0	1.580157	-1.644668	2.180655
27	1	0	3.380005	0.109430	-2.113869
28	1	0	4.013569	-1.265436	1.928321
29	1	0	5.464975	-0.000846	-1.081590
30	1	0	-3.038139	-1.372292	-1.621337
31	1	0	-5.506478	-1.104791	-1.326233
32	1	0	-4.947294	-2.662122	-0.712614
33	1	0	-5.282438	-1.342882	0.405122

Ac-Tyr-NHMe β-1: M06-2X/6-31+G(d), scrf=(smd, solvent=chloroform)

```
NIMG=1 (33.2895 cm-1)
  Zero-point correction=                         0.275587 (Hartree/Particle)
 Thermal correction to Energy=                   0.292876
 Thermal correction to Enthalpy=                 0.293820
 Thermal correction to Gibbs Free Energy=          0.229122
 Sum of electronic and zero-point Energies=          -801.565594
 Sum of electronic and thermal Energies=             -801.548305
 Sum of electronic and thermal Enthalpies=           -801.547361
 Sum of electronic and thermal Free Energies=        -801.612059
Standard orientation:
```

Center Number	Atomic Number	Atomic Type	Coordinates (Angstroms) X	Y	Z
1	6	0	4.952083	-1.150142	0.178576
2	6	0	3.470241	-1.166448	-0.165093
3	8	0	2.982363	-2.152622	-0.711728
4	7	0	2.752637	-0.083772	0.150464
5	6	0	1.338312	0.107980	-0.145173
6	6	0	1.075737	1.621247	-0.110632
7	8	0	1.901053	2.366971	0.421089
8	6	0	0.470228	-0.683341	0.864694
9	6	0	-1.018973	-0.706252	0.561350
10	6	0	-1.506128	-1.501812	-0.496805
11	6	0	-1.918034	0.067934	1.325193
12	6	0	-2.882699	-1.518535	-0.795157
13	6	0	-3.293767	0.050996	1.024661
14	6	0	-3.776724	-0.740553	-0.033373
15	8	0	-5.110288	-0.744625	-0.312217
16	7	0	-0.058725	2.044858	-0.673597
17	6	0	-0.468406	3.443246	-0.736801
18	1	0	5.183375	-1.975970	0.850752
19	1	0	5.542148	-1.273781	-0.729108

20	1	0	5.249974	-0.218992	0.660144
21	1	0	3.175804	0.748085	0.546565
22	1	0	1.144955	-0.248294	-1.159008
23	1	0	0.799709	-1.723030	0.893587
24	1	0	0.630854	-0.296947	1.872470
25	1	0	-0.823222	-2.102038	-1.082511
26	1	0	-1.558067	0.683489	2.137417
27	1	0	-3.236544	-2.134118	-1.609224
28	1	0	-3.981063	0.646218	1.608394
29	1	0	-5.350282	-1.330195	-1.034474
30	1	0	-0.704559	1.350553	-1.024276
31	1	0	-1.329040	3.608626	-0.088214
32	1	0	0.320016	4.133658	-0.430865
33	1	0	-0.757681	3.701418	-1.755513

Ac-Tyr-NHMe β-3: M06-2X/6-31+G(d), scrf=(smd, solvent=chloroform)

NIMG=1 (13.2309 cm^{-1})

```
  Zero-point correction=                          0.274408 (Hartree/Particle)
 Thermal correction to Energy=                   0.291996
 Thermal correction to Enthalpy=                 0.292940
 Thermal correction to Gibbs Free Energy=        0.226634
 Sum of electronic and zero-point Energies=          -801.565957
 Sum of electronic and thermal Energies=             -801.548369
 Sum of electronic and thermal Enthalpies=           -801.547425
 Sum of electronic and thermal Free Energies=        -801.613730
Standard orientation:
```

Center Number	Atomic Number	Atomic Type	Coordinates (Angstroms) X	Y	Z
1	6	0	-1.532329	3.433536	1.266742
2	6	0	-1.721231	2.424558	0.144050
3	8	0	-2.061269	2.811015	-0.971620
4	7	0	-1.500795	1.138245	0.429587
5	6	0	-1.656532	0.024018	-0.497840
6	6	0	-1.813730	-1.254952	0.335193
7	8	0	-1.548851	-1.237028	1.538588
8	6	0	-0.470077	-0.033742	-1.495638
9	6	0	0.927940	-0.085574	-0.892369
10	6	0	1.667235	1.104466	-0.723711
11	6	0	1.497543	-1.318203	-0.507301
12	6	0	2.960879	1.062124	-0.168884
13	6	0	2.791445	-1.359801	0.048475
14	6	0	3.522888	-0.167953	0.218078
15	8	0	4.774915	-0.193611	0.755464
16	7	0	-2.245047	-2.338064	-0.318820
17	6	0	-2.452807	-3.636292	0.314560
18	1	0	-0.774521	4.162908	0.982291
19	1	0	-2.466787	3.963996	1.447206
20	1	0	-1.220105	2.956448	2.195590
21	1	0	-1.251619	0.824330	1.360328
22	1	0	-2.584230	0.173621	-1.053997
23	1	0	-0.589478	-0.891118	-2.158840
24	1	0	-0.522426	0.835645	-2.152867
25	1	0	1.241797	2.054248	-1.015369
26	1	0	0.940569	-2.236319	-0.627400
27	1	0	3.522628	1.975539	-0.039244
28	1	0	3.206355	-2.312705	0.341814

29	1	0	5.069280	-1.075543	0.994689
30	1	0	-2.457578	-2.261940	-1.302206
31	1	0	-1.997987	-4.422890	-0.287721
32	1	0	-2.021405	-3.696884	1.315801
33	1	0	-3.519326	-3.846716	0.396971

Ac-Trp-NHMe PPII-2: M06-2X/6-31+G(d), scrf=(smd, solvent=water)

NIMG=1 (28.3450 cm^{-1})

```
  Zero-point correction=                           0.300227 (Hartree/Particle)
 Thermal correction to Energy=                    0.318589
 Thermal correction to Enthalpy=                  0.319533
 Thermal correction to Gibbs Free Energy=         0.252007
 Sum of electronic and zero-point Energies=          -857.877936
 Sum of electronic and thermal Energies=             -857.859575
 Sum of electronic and thermal Enthalpies=           -857.858630
 Sum of electronic and thermal Free Energies=        -857.926156
Standard orientation:
```

Center Number	Atomic Number	Atomic Type	Coordinates (Angstroms) X	Y	Z
1	6	0	1.808826	3.522718	0.855117
2	6	0	1.806539	2.006736	0.988563
3	8	0	2.201063	1.484647	2.033113
4	7	0	1.357050	1.301508	-0.057345
5	6	0	1.331541	-0.161699	-0.128773
6	6	0	2.740240	-0.738973	-0.355726
7	8	0	3.571037	-0.100087	-1.005516
8	6	0	0.374080	-0.608876	-1.254250
9	6	0	-1.038808	-0.107254	-1.157609
10	6	0	-1.577654	0.883288	-1.905778
11	6	0	-2.086035	-0.522861	-0.226986
12	7	0	-2.884710	1.091988	-1.513818
13	6	0	-3.254971	0.257345	-0.481518
14	6	0	-2.168888	-1.481779	0.810758
15	6	0	-4.443757	0.092751	0.250169
16	6	0	-3.355661	-1.655820	1.552960
17	6	0	-4.492361	-0.871381	1.274058
18	7	0	2.966955	-1.954383	0.151142
19	6	0	4.226433	-2.685167	0.031882
20	1	0	1.417126	3.847560	-0.108803
21	1	0	1.195638	3.969392	1.637456
22	1	0	2.823720	3.906423	0.955393
23	1	0	1.074446	1.786720	-0.895914
24	1	0	0.947506	-0.539083	0.821813
25	1	0	0.779910	-0.314754	-2.224174
26	1	0	0.332583	-1.699134	-1.277435
27	1	0	-1.053164	1.417126	-2.688906
28	1	0	-3.476185	1.793579	-1.953865
29	1	0	-1.306387	-2.092764	1.037284
30	1	0	-5.312720	0.698901	0.033583
31	1	0	-3.392930	-2.396488	2.340696
32	1	0	-5.400290	-1.008281	1.845759
33	1	0	2.220567	-2.413930	0.658423
34	1	0	4.583369	-2.978184	1.019510
35	1	0	4.078994	-3.594074	-0.551947
36	1	0	5.018817	-2.107445	-0.446689

Ac-Trp-NHMe PPII-4: M06-2X/6-31+G(d), scrf=(smd, solvent=water)

```
NIMG=1 (18.0285 cm-1)
  Zero-point correction=                           0.300154 (Hartree/Particle)
 Thermal correction to Energy=                     0.318499
 Thermal correction to Enthalpy=                   0.319443
 Thermal correction to Gibbs Free Energy=          0.251632
 Sum of electronic and zero-point Energies=           -857.879074
 Sum of electronic and thermal Energies=              -857.860729
 Sum of electronic and thermal Enthalpies=            -857.859785
 Sum of electronic and thermal Free Energies=         -857.927596
Standard orientation:
```

Center Number	Atomic Number	Atomic Type	Coordinates (Angstroms) X	Y	Z
1	6	0	-5.296525	-0.232868	-0.660018
2	6	0	-3.990593	-0.218489	0.093574
3	8	0	-3.938683	0.088270	1.294732
4	7	0	-2.890710	-0.551738	-0.598337
5	6	0	-1.566168	-0.519839	-0.011899
6	6	0	-1.076127	0.923564	0.136144
7	8	0	-1.357426	1.785793	-0.709266
8	6	0	-0.601973	-1.300433	-0.905155
9	6	0	0.782770	-1.380598	-0.337046
10	6	0	1.266122	-2.344556	0.514509
11	6	0	1.848631	-0.431700	-0.508969
12	7	0	2.565339	-2.078831	0.869596
13	6	0	2.951491	-0.908296	0.260280
14	6	0	1.976251	0.770186	-1.220003
15	6	0	4.160915	-0.216495	0.313157
16	6	0	3.177689	1.460699	-1.164162
17	6	0	4.259341	0.968604	-0.404895
18	7	0	-0.287695	1.141459	1.191908
19	6	0	0.382734	2.399488	1.456507
20	1	0	-5.182329	-0.531280	-1.705494
21	1	0	-5.980853	-0.926186	-0.164406
22	1	0	-5.731069	0.769253	-0.621614
23	1	0	-2.963942	-0.728694	-1.594749
24	1	0	-1.609148	-0.991238	0.975838
25	1	0	-1.004065	-2.307163	-1.059539
26	1	0	-0.569942	-0.815430	-1.890639
27	1	0	0.742701	-3.217026	0.883782
28	1	0	3.127960	-2.656372	1.483129
29	1	0	1.144011	1.159676	-1.804432
30	1	0	4.995790	-0.589303	0.900193
31	1	0	3.280443	2.391855	-1.712742
32	1	0	5.191657	1.523259	-0.373705
33	1	0	-0.082037	0.361156	1.807243
34	1	0	-0.074933	3.187113	0.859107
35	1	0	0.281845	2.651869	2.514141
36	1	0	1.446904	2.334166	1.205840

Ac-Trp-NHMe PPII-3: M06-2X/6-31+G(d), scrf=(smd, solvent=water)

```
NIMG=1 (30.5054 cm-1)
  Zero-point correction=                           0.300540 (Hartree/Particle)
```

```
 Thermal correction to Energy=                0.318649
 Thermal correction to Enthalpy=              0.319593
 Thermal correction to Gibbs Free Energy=      0.253165
 Sum of electronic and zero-point Energies=        -857.877314
 Sum of electronic and thermal Energies=           -857.859206
 Sum of electronic and thermal Enthalpies=         -857.858262
 Sum of electronic and thermal Free Energies=      -857.924689
Standard orientation:
```

Center Number	Atomic Number	Atomic Type	X	Y	Z
1	6	0	0.570881	3.509561	-0.918751
2	6	0	1.450292	2.508973	-0.183111
3	8	0	2.625489	2.788296	0.061315
4	7	0	0.883731	1.352081	0.178964
5	6	0	1.577753	0.238084	0.825664
6	6	0	2.424788	-0.563559	-0.182029
7	8	0	2.151518	-0.540666	-1.384404
8	6	0	0.538105	-0.655287	1.542724
9	6	0	-0.647699	-1.112958	0.737452
10	6	0	-0.719330	-2.262483	0.027380
11	6	0	-1.904051	-0.407917	0.487664
12	7	0	-1.937207	-2.324687	-0.619728
13	6	0	-2.708895	-1.208691	-0.378880
14	6	0	-2.447845	0.831106	0.904217
15	6	0	-3.985099	-0.804500	-0.807320
16	6	0	-3.725671	1.249223	0.478667
17	6	0	-4.494739	0.433853	-0.375039
18	7	0	3.424742	-1.282569	0.337148
19	6	0	4.335807	-2.123780	-0.435605
20	1	0	-0.448636	3.142596	-1.039917
21	1	0	0.529196	4.449270	-0.368682
22	1	0	0.978856	3.710486	-1.908972
23	1	0	-0.071838	1.157972	-0.087281
24	1	0	2.260536	0.637614	1.579060
25	1	0	1.031712	-1.532842	1.964373
26	1	0	0.147463	-0.111097	2.403373
27	1	0	0.068142	-3.005057	-0.017371
28	1	0	-2.197090	-3.112773	-1.208645
29	1	0	-1.874586	1.467874	1.563634
30	1	0	-4.570716	-1.433097	-1.464113
31	1	0	-4.118336	2.200735	0.811790
32	1	0	-5.474781	0.758146	-0.698043
33	1	0	3.574338	-1.250860	1.338245
34	1	0	4.143057	-2.099681	-1.509357
35	1	0	5.365704	-1.803128	-0.277847
36	1	0	4.256279	-3.160732	-0.108408

(Coordinates in Angstroms)

Ac-Trp-NHMe β-2: M06-2X/6-31+G(d), scrf=(smd, solvent=water)

NIMG=1 (26.7052 cm^{-1})

```
Zero-point correction=                        0.300326 (Hartree/Particle)
 Thermal correction to Energy=                0.318385
 Thermal correction to Enthalpy=              0.319330
 Thermal correction to Gibbs Free Energy=      0.253157
 Sum of electronic and zero-point Energies=        -857.878217
 Sum of electronic and thermal Energies=           -857.860158
 Sum of electronic and thermal Enthalpies=         -857.859214
```

Sum of electronic and thermal Free Energies= -857.925387

Standard orientation:

Center Number	Atomic Number	Atomic Type	Coordinates (Angstroms) X	Y	Z
1	6	0	-0.115457	3.259882	0.697165
2	6	0	0.484971	1.910353	1.063600
3	8	0	0.447764	1.524318	2.233363
4	7	0	1.035308	1.201464	0.071548
5	6	0	1.670887	-0.109701	0.205056
6	6	0	3.070700	-0.047221	-0.421930
7	8	0	3.309520	0.772470	-1.312385
8	6	0	0.808616	-1.182371	-0.499801
9	6	0	-0.607360	-1.328354	-0.017775
10	6	0	-1.008519	-2.121061	1.002503
11	6	0	-1.803656	-0.617806	-0.467826
12	7	0	-2.367712	-1.969985	1.192300
13	6	0	-2.912558	-1.052229	0.320565
14	6	0	-2.066533	0.354777	-1.462174
15	6	0	-4.212786	-0.553145	0.129958
16	6	0	-3.365884	0.865888	-1.661124
17	6	0	-4.438649	0.412880	-0.868005
18	7	0	3.961728	-0.931575	0.035120
19	6	0	5.339528	-1.045495	-0.437721
20	1	0	0.358837	4.053060	1.274521
21	1	0	0.010963	3.487785	-0.361265
22	1	0	-1.182503	3.265584	0.918331
23	1	0	1.114282	1.608138	-0.850899
24	1	0	1.778164	-0.372757	1.259624
25	1	0	0.771523	-0.976314	-1.570823
26	1	0	1.295797	-2.154953	-0.410132
27	1	0	-0.351493	-2.768360	1.570454
28	1	0	-2.873794	-2.482525	1.911069
29	1	0	-1.254749	0.712544	-2.079603
30	1	0	-5.032351	-0.901953	0.743146
31	1	0	-3.539357	1.609250	-2.427816
32	1	0	-5.433738	0.807071	-1.024238
33	1	0	3.674871	-1.577123	0.760806
34	1	0	6.031089	-0.956816	0.400450
35	1	0	5.498437	-2.020723	-0.898484
36	1	0	5.610685	-0.283920	-1.170704

Ac-Trp-NHMe β-1: M06-2X/6-31+G(d), scrf=(smd, solvent=water)

NIMG=1 (17.7012 cm^{-1})

Zero-point correction= 0.300003 (Hartree/Particle)
Thermal correction to Energy= 0.318422
Thermal correction to Enthalpy= 0.319366
Thermal correction to Gibbs Free Energy= 0.250769
Sum of electronic and zero-point Energies= -857.877663
Sum of electronic and thermal Energies= -857.859245
Sum of electronic and thermal Enthalpies= -857.858300
Sum of electronic and thermal Free Energies= -857.926898

Standard orientation:

Center Number	Atomic Number	Atomic Type	Coordinates (Angstroms) X	Y	Z

1	6	0	4.859126	-1.768879	0.208543
2	6	0	3.372870	-1.552929	-0.036762
3	8	0	2.669787	-2.496516	-0.402459
4	7	0	2.893136	-0.320509	0.160384
5	6	0	1.513347	0.104311	-0.057352
6	6	0	1.531582	1.606870	-0.377717
7	8	0	2.470063	2.303157	0.018924
8	6	0	0.659115	-0.238022	1.187693
9	6	0	-0.808128	0.070834	1.101534
10	6	0	-1.432574	1.112342	1.698537
11	6	0	-1.833316	-0.621144	0.324309
12	7	0	-2.770733	1.096472	1.359512
13	6	0	-3.077956	0.053122	0.513205
14	6	0	-1.838606	-1.752083	-0.526387
15	6	0	-4.266294	-0.374588	-0.103701
16	6	0	-3.024024	-2.190379	-1.152481
17	6	0	-4.236866	-1.504670	-0.941861
18	7	0	0.499173	2.078519	-1.081458
19	6	0	0.324759	3.473290	-1.476950
20	1	0	5.355088	-0.853883	0.532522
21	1	0	5.006224	-2.525051	0.979308
22	1	0	5.344352	-2.112600	-0.704615
23	1	0	3.512637	0.441866	0.410740
24	1	0	1.115721	-0.424338	-0.926881
25	1	0	0.739689	-1.304966	1.397846
26	1	0	1.067136	0.268467	2.064131
27	1	0	-0.942808	1.837318	2.337294
28	1	0	-3.424966	1.794282	1.706558
29	1	0	-0.916068	-2.289907	-0.696967
30	1	0	-5.193922	0.156913	0.058714
31	1	0	-3.001467	-3.058587	-1.797644
32	1	0	-5.143504	-1.844459	-1.423990
33	1	0	-0.241093	1.435080	-1.334020
34	1	0	1.172897	4.107002	-1.212892
35	1	0	0.184694	3.541303	-2.555932
36	1	0	-0.562671	3.888603	-0.998626

Ac-Trp-NHMe β-3: M06-2X/6-31+G(d), scrf=(smd, solvent=water)

```
NIMG=1 (22.0347 cm-1)
  Zero-point correction=                         0.300551 (Hartree/Particle)
 Thermal correction to Energy=                   0.318530
 Thermal correction to Enthalpy=                 0.319475
 Thermal correction to Gibbs Free Energy=        0.253379
 Sum of electronic and zero-point Energies=          -857.878462
 Sum of electronic and thermal Energies=             -857.860483
 Sum of electronic and thermal Enthalpies=           -857.859539
 Sum of electronic and thermal Free Energies=        -857.925635
Standard orientation:
```

Center Number	Atomic Number	Atomic Type	Coordinates (Angstroms) X	Y	Z
1	6	0	0.247764	3.470679	1.176334
2	6	0	-0.516295	2.730946	0.088136
3	8	0	-0.678770	3.259596	-1.013046
4	7	0	-0.980170	1.514148	0.387932
5	6	0	-1.740873	0.644469	-0.505258
6	6	0	-2.623524	-0.278598	0.348851

7	8	0	-2.407284	-0.392123	1.558520
8	6	0	-0.787894	-0.140844	-1.443183
9	6	0	0.304013	-0.957436	-0.805162
10	6	0	0.177615	-2.232827	-0.370690
11	6	0	1.667614	-0.552384	-0.465402
12	7	0	1.368173	-2.645183	0.193198
13	6	0	2.323141	-1.652081	0.168017
14	6	0	2.422018	0.634246	-0.628409
15	6	0	3.653720	-1.579224	0.615552
16	6	0	3.756857	0.721234	-0.181516
17	6	0	4.373468	-0.382983	0.439367
18	7	0	-3.597476	-0.925978	-0.297341
19	6	0	-4.536802	-1.854492	0.327035
20	1	0	0.316706	2.885606	2.093439
21	1	0	1.260534	3.690338	0.839150
22	1	0	-0.247406	4.412743	1.410357
23	1	0	-0.870102	1.131793	1.319774
24	1	0	-2.412602	1.256803	-1.111312
25	1	0	-1.372212	-0.800579	-2.086675
26	1	0	-0.308612	0.562705	-2.124808
27	1	0	-0.727039	-2.822342	-0.457498
28	1	0	1.488703	-3.577851	0.581788
29	1	0	1.965712	1.489571	-1.106373
30	1	0	4.122183	-2.429300	1.092213
31	1	0	4.310312	1.640684	-0.317945
32	1	0	5.397576	-0.312440	0.779972
33	1	0	-3.704165	-0.771102	-1.292530
34	1	0	-4.357062	-2.001821	1.393205
35	1	0	-5.557564	-1.489637	0.210691
36	1	0	-4.475147	-2.830097	-0.155800

Ac-Trp-NHMe β-4: M06-2X/6-31+G(d), scrf=(smd, solvent=water)

NIMG=1 (22.6326 cm^{-1})

```
  Zero-point correction=                           0.299909 (Hartree/Particle)
 Thermal correction to Energy=                    0.318297
 Thermal correction to Enthalpy=                  0.319241
 Thermal correction to Gibbs Free Energy=          0.251271
 Sum of electronic and zero-point Energies=           -857.878036
 Sum of electronic and thermal Energies=              -857.859648
 Sum of electronic and thermal Enthalpies=            -857.858703
 Sum of electronic and thermal Free Energies=         -857.926674
Standard orientation:
```

Center Number	Atomic Number	Atomic Type	Coordinates (Angstroms) X	Y	Z
1	6	0	-5.334572	-0.272277	-0.793880
2	6	0	-4.007971	-0.634976	-0.142631
3	8	0	-3.917900	-1.662530	0.531599
4	7	0	-2.981763	0.199541	-0.338554
5	6	0	-1.643702	0.057705	0.228165
6	6	0	-1.039683	1.466427	0.339959
7	8	0	-1.424712	2.355926	-0.423971
8	6	0	-0.801813	-0.891559	-0.660086
9	6	0	0.584375	-1.218336	-0.181768
10	6	0	0.895074	-2.196674	0.699378
11	6	0	1.841967	-0.536832	-0.485242
12	7	0	2.253848	-2.178390	0.942115

13	6	0	2.891080	-1.175933	0.243265
14	6	0	2.205161	0.562159	-1.300164
15	6	0	4.229026	-0.752601	0.163344
16	6	0	3.543618	0.998418	-1.386782
17	6	0	4.555379	0.342380	-0.658051
18	7	0	-0.106457	1.642979	1.278586
19	6	0	0.612042	2.891758	1.514170
20	1	0	-5.269245	0.657125	-1.359680
21	1	0	-5.646265	-1.062825	-1.475992
22	1	0	-6.106102	-0.152952	-0.033586
23	1	0	-3.107605	1.061323	-0.857447
24	1	0	-1.729210	-0.361974	1.233209
25	1	0	-1.329631	-1.838022	-0.784320
26	1	0	-0.714506	-0.469191	-1.662303
27	1	0	0.175568	-2.878491	1.136626
28	1	0	2.699176	-2.838456	1.575806
29	1	0	1.440478	1.078460	-1.863462
30	1	0	5.001378	-1.257322	0.727368
31	1	0	3.794057	1.841661	-2.016421
32	1	0	5.580564	0.680065	-0.727691
33	1	0	0.171258	0.844090	1.835155
34	1	0	0.601224	3.135005	2.576672
35	1	0	1.652685	2.786469	1.205737
36	1	0	0.187785	3.741018	0.976214

Ac-Trp-NHMe PPII-3: M06-2X/6-31+G(d), scrf=(smd, solvent=chloroform)

NIMG=1 (29.2544 cm^{-1})

```
  Zero-point correction=                          0.300421 (Hartree/Particle)
 Thermal correction to Energy=                    0.318725
 Thermal correction to Enthalpy=                  0.319669
 Thermal correction to Gibbs Free Energy=         0.252281
 Sum of electronic and zero-point Energies=          -857.871553
 Sum of electronic and thermal Energies=             -857.853249
 Sum of electronic and thermal Enthalpies=           -857.852305
 Sum of electronic and thermal Free Energies=        -857.919693
```

Standard orientation:

Center Number	Atomic Number	Atomic Type	Coordinates (Angstroms) X	Y	Z
1	6	0	0.771392	3.331491	-1.264213
2	6	0	1.509155	2.412329	-0.302328
3	8	0	2.586112	2.767575	0.172093
4	7	0	0.932197	1.241969	-0.005992
5	6	0	1.558587	0.198726	0.800602
6	6	0	2.496907	-0.654256	-0.068632
7	8	0	2.162270	-0.955332	-1.213984
8	6	0	0.462426	-0.663649	1.467214
9	6	0	-0.713329	-1.053685	0.613343
10	6	0	-0.733579	-2.064475	-0.285069
11	6	0	-2.013750	-0.395494	0.504608
12	7	0	-1.961049	-2.093636	-0.915261
13	6	0	-2.792297	-1.088589	-0.471409
14	6	0	-2.617622	0.720861	1.130730
15	6	0	-4.102274	-0.701490	-0.803230
16	6	0	-3.930666	1.121235	0.805740
17	6	0	-4.673752	0.411079	-0.158232
18	7	0	3.631160	-1.063483	0.508178

19	6	0	4.631063	-1.887828	-0.162188
20	1	0	-0.185363	2.912087	-1.575169
21	1	0	0.588523	4.294244	-0.788100
22	1	0	1.380309	3.498375	-2.152228
23	1	0	0.078779	0.956533	-0.464133
24	1	0	2.161158	0.656814	1.587897
25	1	0	0.901683	-1.566138	1.895710
26	1	0	0.059829	-0.110791	2.316068
27	1	0	0.102896	-2.723745	-0.477951
28	1	0	-2.186062	-2.772408	-1.628508
29	1	0	-2.061293	1.273516	1.873439
30	1	0	-4.664474	-1.247767	-1.546026
31	1	0	-4.369271	1.975904	1.300855
32	1	0	-5.679687	0.720300	-0.403571
33	1	0	3.836306	-0.753785	1.446031
34	1	0	4.289541	-2.278744	-1.122665
35	1	0	5.534716	-1.305053	-0.342130
36	1	0	4.895639	-2.738776	0.465529

Ac-Trp-NHMe β-1: M06-2X/6-31+G(d), scrf=(smd, solvent=chloroform)

```
NIMG=1 (23.9936 cm-1)
Zero-point correction=                           0.300139 (Hartree/Particle)
 Thermal correction to Energy=                     0.318496
 Thermal correction to Enthalpy=                   0.319440
 Thermal correction to Gibbs Free Energy=          0.252027
 Sum of electronic and zero-point Energies=            -857.875365
 Sum of electronic and thermal Energies=               -857.857008
 Sum of electronic and thermal Enthalpies=             -857.856064
 Sum of electronic and thermal Free Energies=          -857.923477
Standard orientation:
```

Center Number	Atomic Number	Atomic Type	Coordinates (Angstroms) X	Y	Z
1	6	0	5.340180	-0.362900	0.718008
2	6	0	3.969627	-0.701896	0.151271
3	8	0	3.789131	-1.786019	-0.398262
4	7	0	3.006252	0.216067	0.280254
5	6	0	1.650795	0.100856	-0.243472
6	6	0	1.099576	1.529995	-0.367970
7	8	0	1.685804	2.458261	0.193563
8	6	0	0.802112	-0.804678	0.682327
9	6	0	-0.566990	-1.171061	0.187334
10	6	0	-0.833569	-2.154873	-0.701246
11	6	0	-1.849144	-0.531615	0.475730
12	7	0	-2.186537	-2.173321	-0.971861
13	6	0	-2.865461	-1.197562	-0.274167
14	6	0	-2.259530	0.549111	1.292040
15	6	0	-4.217456	-0.817962	-0.212345
16	6	0	-3.612283	0.942265	1.361206
17	6	0	-4.591343	0.259450	0.612316
18	7	0	-0.008984	1.680042	-1.096184
19	6	0	-0.680571	2.955538	-1.315151
20	1	0	5.358747	0.624337	1.179156
21	1	0	5.620707	-1.100463	1.469393
22	1	0	6.084087	-0.386470	-0.077835
23	1	0	3.184474	1.132487	0.675459
24	1	0	1.707225	-0.339233	-1.241237
25	1	0	1.330615	-1.741857	0.861579
26	1	0	0.695066	-0.334113	1.660481
27	1	0	-0.084049	-2.811125	-1.124635
28	1	0	-2.604547	-2.839844	-1.605773

29	1	0	-1.518420	1.082864	1.868950
30	1	0	-4.962549	-1.343631	-0.791026
31	1	0	-3.898533	1.770605	1.993782
32	1	0	-5.627052	0.562327	0.669572
33	1	0	-0.464812	0.846890	-1.443760
34	1	0	-0.121156	3.806994	-0.923357
35	1	0	-0.832093	3.119108	-2.382089
36	1	0	-1.657969	2.945666	-0.831828

Ac-Trp-NHMe β-3: M06-2X/6-31+G(d), scrf=(smd, solvent=chloroform)

NIMG=1 (25.3731 cm^{-1})

```
  Zero-point correction=                           0.300789 (Hartree/Particle)
 Thermal correction to Energy=                     0.318708
 Thermal correction to Enthalpy=                   0.319652
 Thermal correction to Gibbs Free Energy=          0.254094
 Sum of electronic and zero-point Energies=           -857.874210
 Sum of electronic and thermal Energies=              -857.856291
 Sum of electronic and thermal Enthalpies=            -857.855347
 Sum of electronic and thermal Free Energies=         -857.920905
```

Standard orientation:

Center Number	Atomic Number	Atomic Type	Coordinates (Angstroms) X	Y	Z
1	6	0	0.334206	3.303136	1.338575
2	6	0	-0.394582	2.623518	0.189397
3	8	0	-0.409024	3.152097	-0.920249
4	7	0	-0.994838	1.458362	0.447525
5	6	0	-1.743962	0.662004	-0.515745
6	6	0	-2.718416	-0.227406	0.265798
7	8	0	-2.537888	-0.420759	1.468987
8	6	0	-0.782760	-0.159184	-1.410430
9	6	0	0.295595	-0.951001	-0.721642
10	6	0	0.134997	-2.158133	-0.132873
11	6	0	1.686323	-0.571882	-0.481482
12	7	0	1.330838	-2.560287	0.426671
13	6	0	2.323936	-1.622024	0.246063
14	6	0	2.477648	0.556124	-0.804125
15	6	0	3.675635	-1.560448	0.627614
16	6	0	3.834369	0.631969	-0.425609
17	6	0	4.434463	-0.424860	0.287803
18	7	0	-3.723269	-0.761127	-0.435280
19	6	0	-4.743411	-1.631558	0.140732
20	1	0	0.236422	2.744780	2.269369
21	1	0	1.393748	3.388992	1.099081
22	1	0	-0.066405	4.304774	1.490693
23	1	0	-0.999286	1.046924	1.372827
24	1	0	-2.341722	1.328058	-1.141496
25	1	0	-1.350610	-0.834414	-2.051339
26	1	0	-0.280999	0.527142	-2.093449
27	1	0	-0.799075	-2.703033	-0.106859
28	1	0	1.432086	-3.435438	0.920207
29	1	0	2.027846	1.373612	-1.349597
30	1	0	4.127925	-2.372487	1.177349
31	1	0	4.415100	1.505389	-0.685858
32	1	0	5.474094	-0.363640	0.575251
33	1	0	-3.797890	-0.544944	-1.418134
34	1	0	-4.532443	-1.913973	1.174087
35	1	0	-5.712965	-1.133417	0.123355

36	1	0	-4.823887	-2.549179	-0.442257

Ac-GPA-NH_2: M06-2X/6-31+G(d), scrf=(smd, solvent=water)

```
NIMG=1 (19.7077 cm-1)
Zero-point correction=                           0.337617 (Hartree/Particle)
 Thermal correction to Energy=                     0.358850
 Thermal correction to Enthalpy=                   0.359794
 Thermal correction to Gibbs Free Energy=          0.285272
 Sum of electronic and zero-point Energies=           -988.636474
 Sum of electronic and thermal Energies=              -988.615241
 Sum of electronic and thermal Enthalpies=            -988.614297
 Sum of electronic and thermal Free Energies=         -988.688819
Standard orientation:
```

Center Number	Atomic Number	Atomic Type	Coordinates (Angstroms) X	Y	Z
1	6	0	-5.622319	-2.204079	0.082569
2	6	0	-4.946451	-0.835756	0.029990
3	8	0	-5.249736	-0.020037	-0.848764
4	1	0	-6.233275	-2.322517	-0.812305
5	1	0	-4.875059	-2.996087	0.140658
6	1	0	-6.263839	-2.252769	0.962071
7	7	0	-3.926010	-0.666930	0.860268
8	6	0	-3.072408	0.512847	0.719339
9	6	0	-1.752677	0.127542	0.040400
10	8	0	-1.518720	-1.046311	-0.271509
11	1	0	-3.743682	-1.349902	1.581909
12	1	0	-2.865235	0.931190	1.704092
13	1	0	-3.585925	1.262305	0.117096
14	7	0	-0.900515	1.100764	-0.186244
15	6	0	0.414985	0.898255	-0.839868
16	6	0	1.335172	-0.077188	-0.098067
17	8	0	1.290188	-0.192676	1.130971
18	6	0	1.043461	2.282217	-0.868437
19	6	0	0.314564	3.064542	0.210444
20	6	0	-1.095240	2.504847	0.210870
21	1	0	0.262218	0.547857	-1.860363
22	1	0	0.856534	2.743819	-1.838474
23	1	0	2.099409	2.205098	-0.607963
24	1	0	0.284603	4.118536	-0.065897
25	1	0	0.780715	2.869043	1.175581
26	1	0	-1.496068	2.539486	1.223466
27	1	0	-1.685053	3.014993	-0.550401
28	7	0	2.236173	-0.669507	-0.848803
29	6	0	3.344546	-1.427809	-0.250248
30	6	0	4.283769	-0.494830	0.514625
31	8	0	4.541453	0.624716	0.061055
32	6	0	4.159385	-2.054546	-1.382871
33	1	0	2.174759	-0.608638	-1.854733
34	1	0	2.959176	-2.202450	0.411690
35	1	0	4.988758	-2.623055	-0.963214
36	1	0	3.521773	-2.719838	-1.965589
37	1	0	4.548930	-1.267851	-2.028800
38	7	0	4.785880	-0.954340	1.644816
39	1	0	4.524761	-1.871772	1.977703
40	1	0	5.431610	-0.389140	2.176725

Ac-GPA-NH_2: M06-2X/6-31+G(d), scrf=(smd, solvent=chloroform)

```
NIMG=1 (21.9221 cm-1)
Zero-point correction=                           0.337784 (Hartree/Particle)
 Thermal correction to Energy=                    0.359274
 Thermal correction to Enthalpy=                  0.360218
 Thermal correction to Gibbs Free Energy=         0.284532
 Sum of electronic and zero-point Energies=          -988.616254
 Sum of electronic and thermal Energies=             -988.594764
 Sum of electronic and thermal Enthalpies=           -988.593820
 Sum of electronic and thermal Free Energies=        -988.669506
Standard orientation:
```

Center Number	Atomic Number	Atomic Type	Coordinates (Angstroms) X	Y	Z
1	6	0	-5.622319	-2.204079	0.082569
2	6	0	-4.946451	-0.835756	0.029990
3	8	0	-5.249736	-0.020037	-0.848764
4	1	0	-6.233275	-2.322517	-0.812305
5	1	0	-4.875059	-2.996087	0.140658
6	1	0	-6.263839	-2.252769	0.962071
7	7	0	-3.926010	-0.666930	0.860268
8	6	0	-3.072408	0.512847	0.719339
9	6	0	-1.752677	0.127542	0.040400
10	8	0	-1.518720	-1.046311	-0.271509
11	1	0	-3.743682	-1.349902	1.581909
12	1	0	-2.865235	0.931190	1.704092
13	1	0	-3.585925	1.262305	0.117096
14	7	0	-0.900515	1.100764	-0.186244
15	6	0	0.414985	0.898255	-0.839868
16	6	0	1.335172	-0.077188	-0.098067
17	8	0	1.290188	-0.192676	1.130971
18	6	0	1.043461	2.282217	-0.868437
19	6	0	0.314564	3.064542	0.210444
20	6	0	-1.095240	2.504847	0.210870
21	1	0	0.262218	0.547857	-1.860363
22	1	0	0.856534	2.743819	-1.838474
23	1	0	2.099409	2.205098	-0.607963
24	1	0	0.284603	4.118536	-0.065897
25	1	0	0.780715	2.869043	1.175581
26	1	0	-1.496068	2.539486	1.223466
27	1	0	-1.685053	3.014993	-0.550401
28	7	0	2.236173	-0.669507	-0.848803
29	6	0	3.344546	-1.427809	-0.250248
30	6	0	4.283769	-0.494830	0.514625
31	8	0	4.541453	0.624716	0.061055
32	6	0	4.159385	-2.054546	-1.382871
33	1	0	2.174759	-0.608638	-1.854733
34	1	0	2.959176	-2.202450	0.411690
35	1	0	4.988758	-2.623055	-0.963214
36	1	0	3.521773	-2.719838	-1.965589
37	1	0	4.548930	-1.267851	-2.028800
38	7	0	4.785880	-0.954340	1.644816
39	1	0	4.524761	-1.871772	1.977703
40	1	0	5.431610	-0.389140	2.176725

Ac-GPS-NH_2: M06-2X/6-31+G(d), scrf=(smd, solvent=water)

```
NIMG=1 (18.0255 cm-1)
Zero-point correction=                           0.342739 (Hartree/Particle)
 Thermal correction to Energy=                    0.365233
```

Thermal correction to Enthalpy= 0.366178
Thermal correction to Gibbs Free Energy= 0.287884
Sum of electronic and zero-point Energies= -1063.826318
Sum of electronic and thermal Energies= -1063.803824
Sum of electronic and thermal Enthalpies= -1063.802879
Sum of electronic and thermal Free Energies= -1063.881173

Standard orientation:

Center Number	Atomic Number	Atomic Type	Coordinates (Angstroms) X	Y	Z
1	6	0	-5.990926	1.817004	0.582905
2	6	0	-4.933940	0.716621	0.380504
3	8	0	-4.678316	-0.088346	1.261486
4	1	0	-6.119184	2.346984	-0.360419
5	1	0	-5.660489	2.510364	1.355934
6	1	0	-6.937090	1.363906	0.878861
7	7	0	-4.310908	0.705362	-0.794768
8	6	0	-3.312582	-0.308543	-1.117117
9	6	0	-2.016773	0.108723	-0.476089
10	8	0	-1.823284	1.293098	-0.205598
11	1	0	-4.525765	1.408789	-1.486853
12	1	0	-3.182504	-0.358948	-2.198692
13	1	0	-3.626098	-1.268861	-0.709096
14	7	0	-1.127242	-0.859838	-0.234933
15	6	0	0.066354	-0.662311	0.558897
16	6	0	1.239201	-0.260588	-0.282990
17	8	0	1.406081	-0.805423	-1.366377
18	6	0	0.348782	-2.059591	1.108387
19	6	0	-0.343801	-3.034574	0.172123
20	6	0	-1.230186	-2.246505	-0.742329
21	1	0	-0.107292	0.052111	1.363774
22	1	0	-0.079709	-2.149711	2.106373
23	1	0	1.422646	-2.244372	1.093874
24	1	0	-0.949063	-3.728918	0.754028
25	1	0	0.404379	-3.565845	-0.417010
26	1	0	-0.832528	-2.290309	-1.756178
27	1	0	-2.258038	-2.594362	-0.636090
28	7	0	2.019443	0.707009	0.207356
29	6	0	3.391466	0.874337	-0.187417
30	6	0	4.229720	-0.343896	0.262402
31	8	0	3.901479	-0.976255	1.234896
32	6	0	3.921248	2.163253	0.433380
33	8	0	3.493204	3.304938	-0.311693
34	1	0	1.650111	1.359465	0.884806
35	1	0	3.440252	0.957549	-1.273464
36	1	0	5.010555	2.133320	0.457075
37	1	0	3.564253	2.245260	1.459963
38	1	0	3.838272	4.101837	0.097316
39	7	0	5.275634	-0.698352	-0.483269
40	1	0	5.467779	-0.211872	-1.348023
41	1	0	5.876337	-1.453334	-0.184531

Ac-GPS-NH_2: M06-2X/6-31+G(d), scrf=(smd, solvent=chloroform)

NIMG=1 (15.7307 cm^{-1})
Zero-point correction= 0.342631 (Hartree/Particle)
Thermal correction to Energy= 0.365260
Thermal correction to Enthalpy= 0.366204

```
 Thermal correction to Gibbs Free Energy=        0.287108
 Sum of electronic and zero-point Energies=        -1063.801186
 Sum of electronic and thermal Energies=           -1063.778558
 Sum of electronic and thermal Enthalpies=         -1063.777614
 Sum of electronic and thermal Free Energies=      -1063.856710
Standard orientation:
```

Center Number	Atomic Number	Atomic Type	Coordinates (Angstroms) X	Y	Z
1	6	0	-5.990926	1.817004	0.582905
2	6	0	-4.933940	0.716621	0.380504
3	8	0	-4.678316	-0.088346	1.261486
4	1	0	-6.119184	2.346984	-0.360419
5	1	0	-5.660489	2.510364	1.355934
6	1	0	-6.937090	1.363906	0.878861
7	7	0	-4.310908	0.705362	-0.794768
8	6	0	-3.312582	-0.308543	-1.117117
9	6	0	-2.016773	0.108723	-0.476089
10	8	0	-1.823284	1.293098	-0.205598
11	1	0	-4.525765	1.408789	-1.486853
12	1	0	-3.182504	-0.358948	-2.198692
13	1	0	-3.626098	-1.268861	-0.709096
14	7	0	-1.127242	-0.859838	-0.234933
15	6	0	0.066354	-0.662311	0.558897
16	6	0	1.239201	-0.260588	-0.282990
17	8	0	1.406081	-0.805423	-1.366377
18	6	0	0.348782	-2.059591	1.108387
19	6	0	-0.343801	-3.034574	0.172123
20	6	0	-1.230186	-2.246505	-0.742329
21	1	0	-0.107292	0.052111	1.363774
22	1	0	-0.079709	-2.149711	2.106373
23	1	0	1.422646	-2.244372	1.093874
24	1	0	-0.949063	-3.728918	0.754028
25	1	0	0.404379	-3.565845	-0.417010
26	1	0	-0.832528	-2.290309	-1.756178
27	1	0	-2.258038	-2.594362	-0.636090
28	7	0	2.019443	0.707009	0.207356
29	6	0	3.391466	0.874337	-0.187417
30	6	0	4.229720	-0.343896	0.262402
31	8	0	3.901479	-0.976255	1.234896
32	6	0	3.921248	2.163253	0.433380
33	8	0	3.493204	3.304938	-0.311693
34	1	0	1.650111	1.359465	0.884806
35	1	0	3.440252	0.957549	-1.273464
36	1	0	5.010555	2.133320	0.457075
37	1	0	3.564253	2.245260	1.459963
38	1	0	3.838272	4.101837	0.097316
39	7	0	5.275634	-0.698352	-0.483269
40	1	0	5.467779	-0.211872	-1.348023
41	1	0	5.876337	-1.453334	-0.184531

Ac-LVF-NH_2: M06-2X/6-31+G(d), scrf=(smd, solvent=water)

NIMG=1 (11.9529 cm^{-1})

```
Zero-point correction=                          0.554063 (Hartree/Particle)
 Thermal correction to Energy=                    0.586909
 Thermal correction to Enthalpy=                  0.587853
 Thermal correction to Gibbs Free Energy=         0.486271
```

Sum of electronic and zero-point Energies= -1377.758871
Sum of electronic and thermal Energies= -1377.726025
Sum of electronic and thermal Enthalpies= -1377.725081
Sum of electronic and thermal Free Energies= -1377.826663

Standard orientation:

Center Number	Atomic Number	Atomic Type	Coordinates (Angstroms) X	Y	Z
1	6	0	-6.146960	-2.001546	0.789628
2	6	0	-5.072302	-1.378831	-0.087299
3	8	0	-5.213268	-1.375535	-1.306545
4	7	0	-4.006865	-0.853940	0.527390
5	6	0	-2.823843	-0.337075	-0.150241
6	6	0	-1.627443	-1.037135	0.497863
7	8	0	-1.664973	-1.304240	1.701065
8	6	0	-2.701342	1.198206	0.024212
9	6	0	-3.785357	2.033461	-0.702710
10	6	0	-5.113619	2.099796	0.086963
11	6	0	-3.260276	3.441391	-1.062432
12	1	0	-3.866334	-0.974685	1.521369
13	7	0	-0.591830	-1.300578	-0.299931
14	6	0	0.690433	-1.831564	0.137555
15	6	0	1.741974	-0.971552	-0.575438
16	8	0	1.532523	-0.607442	-1.735141
17	6	0	0.875772	-3.321281	-0.284207
18	6	0	2.261700	-3.907138	0.065055
19	6	0	-0.222262	-4.220059	0.317669
20	1	0	-0.580802	-0.973714	-1.257607
21	7	0	2.833990	-0.657349	0.123501
22	6	0	3.942227	0.142089	-0.381454
23	6	0	5.209853	-0.677190	-0.130327
24	8	0	5.291449	-1.405193	0.850295
25	6	0	4.010009	1.479875	0.392535
26	6	0	2.746077	2.331931	0.397013
27	6	0	1.994022	2.534372	-0.781763
28	6	0	2.389090	3.047023	1.560851
29	6	0	0.883376	3.367632	-0.765643
30	6	0	1.278109	3.879260	1.554842
31	6	0	0.525228	4.035189	0.398139
32	1	0	3.007338	-1.084896	1.022180
33	7	0	6.211054	-0.553551	-0.981214
34	1	0	6.134745	0.014172	-1.806883
35	1	0	-5.994888	-1.777938	1.845398
36	1	0	-2.841404	-0.587581	-1.213576
37	1	0	-1.728860	1.476326	-0.387875
38	1	0	-2.644716	1.469741	1.079751
39	1	0	-4.001353	1.532975	-1.648751
40	1	0	-5.457705	3.117073	0.270387
41	1	0	-5.908168	1.589754	-0.459161
42	1	0	-5.036561	1.620116	1.062543
43	1	0	-3.976270	4.234850	-0.849498
44	1	0	-2.347184	3.689748	-0.522342
45	1	0	-3.032516	3.504748	-2.126991
46	1	0	0.802963	-1.722769	1.218713
47	1	0	0.775087	-3.380537	-1.370311
48	1	0	2.316131	-4.966920	-0.186537
49	1	0	3.069303	-3.419455	-0.482210
50	1	0	2.476946	-3.813192	1.130149
51	1	0	-0.072146	-5.267580	0.055331
52	1	0	-0.241303	-4.146852	1.405780

53	1	0	-1.212137	-3.942418	-0.045565
54	1	0	3.839887	0.326947	-1.452258
55	1	0	4.813376	2.093498	-0.017160
56	1	0	4.296973	1.276847	1.426408
57	1	0	2.257073	2.027283	-1.698858
58	1	0	2.970245	2.946400	2.465946
59	1	0	0.299090	3.499142	-1.665156
60	1	0	1.000454	4.412450	2.452674
61	1	0	-0.335824	4.687987	0.402414
62	1	0	-6.147859	-3.083632	0.657875
63	1	0	-7.124572	-1.621735	0.493409
64	1	0	7.033907	-1.100060	-0.790060

Ac-LVF-NH_2: M06-2X/6-31+G(d), scrf=(smd, solvent=chloroform)

```
NIMG=1 (17.3775 cm-1)
Zero-point correction=                           0.554518 (Hartree/Particle)
 Thermal correction to Energy=                    0.587503
 Thermal correction to Enthalpy=                  0.588447
 Thermal correction to Gibbs Free Energy=         0.486423
 Sum of electronic and zero-point Energies=          -1377.752610
 Sum of electronic and thermal Energies=             -1377.719625
 Sum of electronic and thermal Enthalpies=           -1377.718681
 Sum of electronic and thermal Free Energies=        -1377.820704
Standard orientation:
```

Center Number	Atomic Number	Atomic Type	Coordinates (Angstroms) X	Y	Z
1	6	0	-6.146960	-2.001546	0.789628
2	6	0	-5.072302	-1.378831	-0.087299
3	8	0	-5.213268	-1.375535	-1.306545
4	7	0	-4.006865	-0.853940	0.527390
5	6	0	-2.823843	-0.337075	-0.150241
6	6	0	-1.627443	-1.037135	0.497863
7	8	0	-1.664973	-1.304240	1.701065
8	6	0	-2.701342	1.198206	0.024212
9	6	0	-3.785357	2.033461	-0.702710
10	6	0	-5.113619	2.099796	0.086963
11	6	0	-3.260276	3.441391	-1.062432
12	1	0	-3.866334	-0.974685	1.521369
13	7	0	-0.591830	-1.300578	-0.299931
14	6	0	0.690433	-1.831564	0.137555
15	6	0	1.741974	-0.971552	-0.575438
16	8	0	1.532523	-0.607442	-1.735141
17	6	0	0.875772	-3.321281	-0.284207
18	6	0	2.261700	-3.907138	0.065055
19	6	0	-0.222262	-4.220059	0.317669
20	1	0	-0.580802	-0.973714	-1.257607
21	7	0	2.833990	-0.657349	0.123501
22	6	0	3.942227	0.142089	-0.381454
23	6	0	5.209853	-0.677190	-0.130327
24	8	0	5.291449	-1.405193	0.850295
25	6	0	4.010009	1.479875	0.392535
26	6	0	2.746077	2.331931	0.397013
27	6	0	1.994022	2.534372	-0.781763
28	6	0	2.389090	3.047023	1.560851
29	6	0	0.883376	3.367632	-0.765643
30	6	0	1.278109	3.879260	1.554842
31	6	0	0.525228	4.035189	0.398139
32	1	0	3.007338	-1.084896	1.022180
33	7	0	6.211054	-0.553551	-0.981214
34	1	0	6.134745	0.014172	-1.806883
35	1	0	-5.994888	-1.777938	1.845398

36	1	0	-2.841404	-0.587581	-1.213576
37	1	0	-1.728860	1.476326	-0.387875
38	1	0	-2.644716	1.469741	1.079751
39	1	0	-4.001353	1.532975	-1.648751
40	1	0	-5.457705	3.117073	0.270387
41	1	0	-5.908168	1.589754	-0.459161
42	1	0	-5.036561	1.620116	1.062543
43	1	0	-3.976270	4.234850	-0.849498
44	1	0	-2.347184	3.689748	-0.522342
45	1	0	-3.032516	3.504748	-2.126991
46	1	0	0.802963	-1.722769	1.218713
47	1	0	0.775087	-3.380537	-1.370311
48	1	0	2.316131	-4.966920	-0.186537
49	1	0	3.069303	-3.419455	-0.482210
50	1	0	2.476946	-3.813192	1.130149
51	1	0	-0.072146	-5.267580	0.055331
52	1	0	-0.241303	-4.146852	1.405780
53	1	0	-1.212137	-3.942418	-0.045565
54	1	0	3.839887	0.326947	-1.452258
55	1	0	4.813376	2.093498	-0.017160
56	1	0	4.296973	1.276847	1.426408
57	1	0	2.257073	2.027283	-1.698858
58	1	0	2.970245	2.946400	2.465946
59	1	0	0.299090	3.499142	-1.665156
60	1	0	1.000454	4.412450	2.452674
61	1	0	-0.335824	4.687987	0.402414
62	1	0	-6.147859	-3.083632	0.657875
63	1	0	-7.124572	-1.621735	0.493409
64	1	0	7.033907	-1.100060	-0.790060

Ac-ITY-NH_2: M06-2X/6-31+G(d), scrf=(smd, solvent=chloroform)

NIMG=1 (18.2191 cm^{-1})
Zero-point correction= 0.534772 (Hartree/Particle)
Thermal correction to Energy= 0.568445
Thermal correction to Enthalpy= 0.569390
Thermal correction to Gibbs Free Energy= 0.467450
Sum of electronic and zero-point Energies= -1488.874951
Sum of electronic and thermal Energies= -1488.841278
Sum of electronic and thermal Enthalpies= -1488.840333
Sum of electronic and thermal Free Energies= -1488.942273
Standard orientation:

Center Number	Atomic Number	Atomic Type	Coordinates (Angstroms) X	Y	Z
1	6	0	5.733924	-1.636127	-1.985835
2	6	0	4.883998	-1.350862	-0.757976
3	8	0	5.242280	-1.782420	0.333889
4	7	0	3.767625	-0.634331	-0.931638
5	6	0	2.789826	-0.359950	0.114017
6	6	0	1.446852	-0.861337	-0.433807
7	8	0	1.072385	-0.485239	-1.547889
8	6	0	2.658454	1.168303	0.404708
9	6	0	4.006203	1.838712	0.764224
10	6	0	1.591734	1.502620	1.471868
11	6	0	4.631178	1.375964	2.089918
12	7	0	0.762597	-1.700799	0.345011
13	6	0	-0.580024	-2.175415	0.042818
14	6	0	-1.603495	-1.150061	0.558780
15	8	0	-1.501474	-0.725188	1.710835
16	6	0	-0.855571	-3.531588	0.744223

17	8	0	0.168658	-4.441713	0.382486
18	6	0	-2.208791	-4.176340	0.389842
19	7	0	-2.580403	-0.805278	-0.282373
20	6	0	-3.708223	0.051505	0.059134
21	6	0	-4.975897	-0.774468	-0.166499
22	8	0	-5.013937	-1.622806	-1.047006
23	6	0	-3.706129	1.299626	-0.848267
24	6	0	-2.518041	2.226999	-0.653902
25	6	0	-1.356040	2.069280	-1.438784
26	6	0	-2.567907	3.249897	0.316072
27	6	0	-0.262021	2.936449	-1.263440
28	6	0	-1.469843	4.113957	0.493692
29	6	0	-0.316443	3.957598	-0.298655
30	8	0	0.755105	4.783354	-0.134223
31	7	0	-6.022643	-0.529250	0.600009
32	1	0	5.302484	-1.216063	-2.893397
33	1	0	3.448765	-0.371469	-1.852857
34	1	0	3.027014	-0.895698	1.035524
35	1	0	2.322750	1.650692	-0.516032
36	1	0	3.862667	2.919399	0.803297
37	1	0	4.727196	1.681517	-0.038299
38	1	0	1.564678	2.573571	1.674012
39	1	0	0.586189	1.225299	1.151395
40	1	0	1.788317	0.990638	2.413673
41	1	0	5.644499	1.765051	2.190884
42	1	0	4.058363	1.729801	2.946964
43	1	0	4.698025	0.289841	2.152034
44	1	0	1.136394	-1.988117	1.233908
45	1	0	-0.684559	-2.298337	-1.037198
46	1	0	-0.809149	-3.395376	1.828112
47	1	0	0.022874	-5.283650	0.821733
48	1	0	-2.325238	-5.151174	0.863073
49	1	0	-3.049323	-3.564274	0.720029
50	1	0	-2.309721	-4.316047	-0.687009
51	1	0	-2.667880	-1.270113	-1.173936
52	1	0	-3.661339	0.361869	1.105424
53	1	0	-4.613747	1.878253	-0.669617
54	1	0	-3.760531	1.003173	-1.897244
55	1	0	-1.284995	1.275998	-2.169198
56	1	0	-3.445501	3.374600	0.932980
57	1	0	0.625757	2.805187	-1.864130
58	1	0	-1.525872	4.888666	1.243447
59	1	0	0.624710	5.448120	0.544578
60	1	0	-5.978856	0.132310	1.354706
61	1	0	-6.845123	-1.082670	0.429754
62	1	0	6.729423	-1.216606	-1.845684
63	1	0	5.834891	-2.713249	-2.115512

Ac-VVV-NH_2: M06-2X/6-31+G(d), scrf=(smd, solvent=water)

NIMG=1 (21.2372 cm^{-1})

```
Zero-point correction=                           0.500298 (Hartree/Particle)
 Thermal correction to Energy=                     0.529869
 Thermal correction to Enthalpy=                   0.530813
 Thermal correction to Gibbs Free Energy=          0.438584
 Sum of electronic and zero-point Energies=          -1186.145615
 Sum of electronic and thermal Energies=             -1186.116044
 Sum of electronic and thermal Enthalpies=           -1186.115100
 Sum of electronic and thermal Free Energies=        -1186.207329
```

Standard orientation:

Center Number	Atomic Number	Atomic Type	Coordinates (Angstroms) X	Y	Z
1	6	0	6.447702	0.940249	1.054983
2	6	0	5.405123	0.398709	0.089776
3	8	0	5.645174	0.376014	-1.113659
4	7	0	4.255209	-0.036717	0.613234
5	6	0	3.096252	-0.476335	-0.154480
6	6	0	1.889737	0.249795	0.459624
7	8	0	1.875956	0.474371	1.672169
8	6	0	2.884160	-2.018500	-0.038859
9	6	0	1.587895	-2.541837	-0.698257
10	6	0	4.088806	-2.792646	-0.608878
11	7	0	0.913647	0.597708	-0.380897
12	6	0	-0.359727	1.185341	0.010145
13	6	0	-1.442497	0.282669	-0.601668
14	8	0	-1.218747	-0.286889	-1.672084
15	6	0	-0.508928	2.629198	-0.559875
16	6	0	-1.877987	3.289164	-0.283845
17	6	0	0.612171	3.551936	-0.042184
18	7	0	-2.580406	0.166843	0.083726
19	6	0	-3.759056	-0.561361	-0.360255
20	6	0	-4.973925	0.333170	-0.071114
21	8	0	-4.904074	1.228187	0.762332
22	6	0	-3.914898	-1.913278	0.399660
23	6	0	-2.796307	-2.902210	0.023780
24	6	0	-4.029001	-1.788946	1.935924
25	7	0	-6.088205	0.105987	-0.741215
26	1	0	6.508679	2.024119	0.963588
27	1	0	6.213919	0.688631	2.088863
28	1	0	7.423648	0.522115	0.810807
29	1	0	-6.868250	0.706504	-0.534105
30	1	0	-6.144868	-0.612137	-1.441724
31	1	0	4.043550	0.093446	1.593820
32	1	0	3.193052	-0.185257	-1.203032
33	1	0	2.816988	-2.270628	1.021529
34	1	0	1.539780	-3.630728	-0.665535
35	1	0	0.690815	-2.180944	-0.194059
36	1	0	1.524030	-2.243731	-1.745517
37	1	0	3.937009	-3.870792	-0.552767
38	1	0	4.265549	-2.534151	-1.653768
39	1	0	5.004716	-2.571959	-0.060566
40	1	0	0.931304	0.273187	-1.338390
41	1	0	-0.469162	1.192653	1.096983
42	1	0	-0.401548	2.576633	-1.645498
43	1	0	-1.891972	4.324309	-0.626349
44	1	0	-2.694040	2.784920	-0.802146
45	1	0	-2.111727	3.294829	0.781448
46	1	0	0.499649	4.569013	-0.418757
47	1	0	0.615326	3.598337	1.047704
48	1	0	1.597285	3.205844	-0.357118
49	1	0	-2.756460	0.750269	0.891031
50	1	0	-3.710888	-0.737255	-1.437589
51	1	0	-4.846499	-2.366679	0.056391
52	1	0	-2.968611	-3.884504	0.463865
53	1	0	-2.730685	-3.032651	-1.057093
54	1	0	-1.822170	-2.554549	0.368946
55	1	0	-4.127994	-2.769594	2.401734
56	1	0	-3.146637	-1.313812	2.365767

57	1	0	-4.899098	-1.206352	2.240697

Ac-VVV-NH_2: M06-2X/6-31+G(d), scrf=(smd, solvent=chloroform)

```
NIMG=1 (19.4145 cm-1)
Zero-point correction=                           0.500236 (Hartree/Particle)
 Thermal correction to Energy=                    0.530105
 Thermal correction to Enthalpy=                  0.531049
 Thermal correction to Gibbs Free Energy=         0.437280
 Sum of electronic and zero-point Energies=          -1186.137308
 Sum of electronic and thermal Energies=             -1186.107439
 Sum of electronic and thermal Enthalpies=           -1186.106494
 Sum of electronic and thermal Free Energies=        -1186.200263
Standard orientation:
```

Center Number	Atomic Number	Atomic Type	Coordinates (Angstroms) X	Y	Z
1	6	0	6.447702	0.940249	1.054983
2	6	0	5.405123	0.398709	0.089776
3	8	0	5.645174	0.376014	-1.113659
4	7	0	4.255209	-0.036717	0.613234
5	6	0	3.096252	-0.476335	-0.154480
6	6	0	1.889737	0.249795	0.459624
7	8	0	1.875956	0.474371	1.672169
8	6	0	2.884160	-2.018500	-0.038859
9	6	0	1.587895	-2.541837	-0.698257
10	6	0	4.088806	-2.792646	-0.608878
11	7	0	0.913647	0.597708	-0.380897
12	6	0	-0.359727	1.185341	0.010145
13	6	0	-1.442497	0.282669	-0.601668
14	8	0	-1.218747	-0.286889	-1.672084
15	6	0	-0.508928	2.629198	-0.559875
16	6	0	-1.877987	3.289164	-0.283845
17	6	0	0.612171	3.551936	-0.042184
18	7	0	-2.580406	0.166843	0.083726
19	6	0	-3.759056	-0.561361	-0.360255
20	6	0	-4.973925	0.333170	-0.071114
21	8	0	-4.904074	1.228187	0.762332
22	6	0	-3.914898	-1.913278	0.399660
23	6	0	-2.796307	-2.902210	0.023780
24	6	0	-4.029001	-1.788946	1.935924
25	7	0	-6.088205	0.105987	-0.741215
26	1	0	6.508679	2.024119	0.963588
27	1	0	6.213919	0.688631	2.088863
28	1	0	7.423648	0.522115	0.810807
29	1	0	-6.868250	0.706504	-0.534105
30	1	0	-6.144868	-0.612137	-1.441724
31	1	0	4.043550	0.093446	1.593820
32	1	0	3.193052	-0.185257	-1.203032
33	1	0	2.816988	-2.270628	1.021529
34	1	0	1.539780	-3.630728	-0.665535
35	1	0	0.690815	-2.180944	-0.194059
36	1	0	1.524030	-2.243731	-1.745517
37	1	0	3.937009	-3.870792	-0.552767
38	1	0	4.265549	-2.534151	-1.653768
39	1	0	5.004716	-2.571959	-0.060566
40	1	0	0.931304	0.273187	-1.338390
41	1	0	-0.469162	1.192653	1.096983
42	1	0	-0.401548	2.576633	-1.645498
43	1	0	-1.891972	4.324309	-0.626349
44	1	0	-2.694040	2.784920	-0.802146
45	1	0	-2.111727	3.294829	0.781448
46	1	0	0.499649	4.569013	-0.418757
47	1	0	0.615326	3.598337	1.047704
48	1	0	1.597285	3.205844	-0.357118

49	1	0	-2.756460	0.750269	0.891031
50	1	0	-3.710888	-0.737255	-1.437589
51	1	0	-4.846499	-2.366679	0.056391
52	1	0	-2.968611	-3.884504	0.463865
53	1	0	-2.730685	-3.032651	-1.057093
54	1	0	-1.822170	-2.554549	0.368946
55	1	0	-4.127994	-2.769594	2.401734
56	1	0	-3.146637	-1.313812	2.365767
57	1	0	-4.899098	-1.206352	2.240697

Ac-LVI-NH_2: M06-2X/6-31+G(d), scrf=(smd, solvent=water)

```
NIMG=1 (25.3039 cm-1)
Zero-point correction=                           0.557288 (Hartree/Particle)
 Thermal correction to Energy=                     0.589729
 Thermal correction to Enthalpy=                   0.590673
 Thermal correction to Gibbs Free Energy=          0.492382
 Sum of electronic and zero-point Energies=          -1264.672810
 Sum of electronic and thermal Energies=             -1264.640368
 Sum of electronic and thermal Enthalpies=           -1264.639424
 Sum of electronic and thermal Free Energies=        -1264.737715
Standard orientation:
```

Center Number	Atomic Number	Atomic Type	Coordinates (Angstroms) X	Y	Z
1	6	0	-6.436700	0.921665	-1.062774
2	6	0	-5.287060	0.471542	-0.174841
3	8	0	-5.509344	0.167371	0.993119
4	7	0	-4.067646	0.428162	-0.721178
5	6	0	-2.838580	0.110514	-0.004051
6	6	0	-1.756248	1.056015	-0.524647
7	8	0	-1.810241	1.463912	-1.686125
8	6	0	-2.416073	-1.354865	-0.281059
9	6	0	-3.215707	-2.423025	0.494926
10	6	0	-2.884229	-3.842170	-0.015040
11	6	0	-3.024835	-2.292967	2.021689
12	7	0	-0.765689	1.317864	0.327405
13	6	0	0.529867	1.870406	-0.033582
14	6	0	1.529647	0.882604	0.584885
15	8	0	1.300863	0.428765	1.707994
16	6	0	0.753633	3.284201	0.578536
17	6	0	2.171206	3.850112	0.343652
18	6	0	-0.292369	4.287665	0.056242
19	7	0	2.575324	0.529010	-0.162940
20	6	0	3.598359	-0.424885	0.241012
21	6	0	4.928355	0.228511	-0.161155
22	8	0	5.013247	0.858382	-1.207926
23	6	0	3.445162	-1.791969	-0.504843
24	6	0	2.041058	-2.413511	-0.282880
25	6	0	4.539148	-2.815930	-0.119635
26	6	0	1.767558	-3.683961	-1.102112
27	7	0	5.960596	0.105039	0.653465
28	1	0	-6.112793	1.131166	-2.081878
29	1	0	-3.894494	0.774729	-1.655781
30	1	0	-2.964402	0.288255	1.066078
31	1	0	-1.359311	-1.487217	-0.038889
32	1	0	-2.487830	-1.538873	-1.354622
33	1	0	-4.273531	-2.257694	0.284186
34	1	0	-3.797042	-4.387397	-0.257982

35	1	0	-2.273855	-3.824625	-0.918545
36	1	0	-2.338382	-4.436780	0.717564
37	1	0	-2.864699	-3.252572	2.512968
38	1	0	-2.176237	-1.658043	2.278314
39	1	0	-3.910286	-1.847810	2.478372
40	1	0	-0.756859	0.876481	1.238680
41	1	0	0.654157	1.897361	-1.118629
42	1	0	0.617240	3.212814	1.659942
43	1	0	2.258506	4.865520	0.731877
44	1	0	2.940413	3.260477	0.843868
45	1	0	2.415372	3.884106	-0.718803
46	1	0	-0.131404	5.284676	0.467244
47	1	0	-0.257989	4.364948	-1.031194
48	1	0	-1.305072	3.989894	0.329726
49	1	0	2.780984	0.997553	-1.035655
50	1	0	3.581517	-0.579933	1.321770
51	1	0	3.553169	-1.597139	-1.574567
52	1	0	1.260854	-1.696394	-0.536296
53	1	0	1.903124	-2.629378	0.777622
54	1	0	4.423241	-3.752931	-0.664074
55	1	0	5.542285	-2.458799	-0.350939
56	1	0	4.502876	-3.049417	0.944647
57	1	0	0.698594	-3.806987	-1.278913
58	1	0	2.258704	-3.650544	-2.074645
59	1	0	2.115349	-4.573729	-0.577426
60	1	0	5.880884	-0.386042	1.526865
61	1	0	-6.889728	1.822663	-0.649279
62	1	0	-7.197670	0.142247	-1.093518
63	1	0	6.816864	0.551734	0.372206

Ac-LVI-NH$_2$: M06-2X/6-31+G(d), scrf=(smd, solvent=chloroform)

```
NIMG=1 (12.2437 cm-1)
Zero-point correction=                           0.557435 (Hartree/Particle)
 Thermal correction to Energy=                     0.590332
 Thermal correction to Enthalpy=                   0.591276
 Thermal correction to Gibbs Free Energy=          0.488951
 Sum of electronic and zero-point Energies=          -1264.666340
 Sum of electronic and thermal Energies=             -1264.633443
 Sum of electronic and thermal Enthalpies=           -1264.632499
 Sum of electronic and thermal Free Energies=        -1264.734824
Standard orientation:
```

Center Number	Atomic Number	Atomic Type	Coordinates (Angstroms) X	Y	Z
1	6	0	-6.436700	0.921665	-1.062774
2	6	0	-5.287060	0.471542	-0.174841
3	8	0	-5.509344	0.167371	0.993119
4	7	0	-4.067646	0.428162	-0.721178
5	6	0	-2.838580	0.110514	-0.004051
6	6	0	-1.756248	1.056015	-0.524647
7	8	0	-1.810241	1.463912	-1.686125
8	6	0	-2.416073	-1.354865	-0.281059
9	6	0	-3.215707	-2.423025	0.494926
10	6	0	-2.884229	-3.842170	-0.015040
11	6	0	-3.024835	-2.292967	2.021689
12	7	0	-0.765689	1.317864	0.327405
13	6	0	0.529867	1.870406	-0.033582

14	6	0	1.529647	0.882604	0.584885
15	8	0	1.300863	0.428765	1.707994
16	6	0	0.753633	3.284201	0.578536
17	6	0	2.171206	3.850112	0.343652
18	6	0	-0.292369	4.287665	0.056242
19	7	0	2.575324	0.529010	-0.162940
20	6	0	3.598359	-0.424885	0.241012
21	6	0	4.928355	0.228511	-0.161155
22	8	0	5.013247	0.858382	-1.207926
23	6	0	3.445162	-1.791969	-0.504843
24	6	0	2.041058	-2.413511	-0.282880
25	6	0	4.539148	-2.815930	-0.119635
26	6	0	1.767558	-3.683961	-1.102112
27	7	0	5.960596	0.105039	0.653465
28	1	0	-6.112793	1.131166	-2.081878
29	1	0	-3.894494	0.774729	-1.655781
30	1	0	-2.964402	0.288255	1.066078
31	1	0	-1.359311	-1.487217	-0.038889
32	1	0	-2.487830	-1.538873	-1.354622
33	1	0	-4.273531	-2.257694	0.284186
34	1	0	-3.797042	-4.387397	-0.257982
35	1	0	-2.273855	-3.824625	-0.918545
36	1	0	-2.338382	-4.436780	0.717564
37	1	0	-2.864699	-3.252572	2.512968
38	1	0	-2.176237	-1.658043	2.278314
39	1	0	-3.910286	-1.847810	2.478372
40	1	0	-0.756859	0.876481	1.238680
41	1	0	0.654157	1.897361	-1.118629
42	1	0	0.617240	3.212814	1.659942
43	1	0	2.258506	4.865520	0.731877
44	1	0	2.940413	3.260477	0.843868
45	1	0	2.415372	3.884106	-0.718803
46	1	0	-0.131404	5.284676	0.467244
47	1	0	-0.257989	4.364948	-1.031194
48	1	0	-1.305072	3.989894	0.329726
49	1	0	2.780984	0.997553	-1.035655
50	1	0	3.581517	-0.579933	1.321770
51	1	0	3.553169	-1.597139	-1.574567
52	1	0	1.260854	-1.696394	-0.536296
53	1	0	1.903124	-2.629378	0.777622
54	1	0	4.423241	-3.752931	-0.664074
55	1	0	5.542285	-2.458799	-0.350939
56	1	0	4.502876	-3.049417	0.944647
57	1	0	0.698594	-3.806987	-1.278913
58	1	0	2.258704	-3.650544	-2.074645
59	1	0	2.115349	-4.573729	-0.577426
60	1	0	5.880884	-0.386042	1.526865
61	1	0	-6.889728	1.822663	-0.649279
62	1	0	-7.197670	0.142247	-1.093518
63	1	0	6.816864	0.551734	0.372206

Supplementary References

1. Grdadolnik, J., Mohacek-Grosev, V., Baldwin, R. L. & Avbelj, F. Populations of the Three Major Backbone Conformations in 19 Amino Acid Dipeptides. *Proc. Natl. Acad. Sci.* **108**, 1794–1798 (2011).
2. Sawyer, L., Shotton, D. M., Campbell, J. W., Wendell, P. L., Muirhead, H., Watson, H. C., Diamond, R. & Ladner, R. C. The Atomic Structure of Crystalline Porcine Pancreatic Elastase at 2.5 Å Resolution: Comparisons with the Structure of α-Chymotrypsin. *J. Mol. Biol.* **118**, 137–208 (1978).
3. M. J. Frisch, G. W. Trucks, H. B. Schlegel, G. E. Scuseria, M. A. Robb, J. R. Cheeseman, G. Scalmani, V. Barone, B. Mennucci, G. A. Petersson, H. Nakatsuji, M. Caricato, X. Li, H. P. Hratchian, A. F. Izmaylov, J. Bloino, G. Zheng, J. L. Sonnenberg, M. Hada, M. Ehara, K. Toyota, R. Fukuda, J. Hasegawa, M. Ishida, T. Nakajima, Y. Honda, O. Kitao, H. Nakai, T. Vreven, J. A., Jr. Montgomery, J. E. Peralta, F. Ogliaro, M. Bearpark, J. J. Heyd, E. Brothers, K. N. Kudin, V. N. Staroverov, R. Kobayashi, J. Normand, K. Raghavachari, A. Rendell, J. C. Burant, S. S. Iyengar, J. Tomasi, M. Cossi, N. Rega, J. M. Millam, M. Klene, J. E. Knox, J. B. Cross, V. Bakken, C. Adamo, J. Jaramillo, R. Gomperts, R. E. Stratmann, O. Yazyev, A. J.Austin, R. Cammi, C. Pomelli, J. W. Ochterski, R. L. Martin, K. Morokuma, V. G. Zakrzewski, G. A. Voth, P. Salvador, J. J. Dannenberg, S. Dapprich, A. D. Daniels, O. Farkas, J. B. Foresman, J. V. Ortiz, J. Cioslowski, D. J. Fox, Gaussian 09, revision D.01, Gaussian, Inc., Wallingford, CT, 2013.
